# TMSBr-Promoted Cascade Cyclization of *ortho*-Propynol Phenyl Azides for the Synthesis of 4-Bromo Quinolines and Its Applications

**DOI:** 10.3390/molecules24213999

**Published:** 2019-11-05

**Authors:** Fengyan Jin, Tao Yang, Xian-Rong Song, Jiang Bai, Ruchun Yang, Haixin Ding, Qiang Xiao

**Affiliations:** Institute of Organic Chemistry, Jiangxi Science & Technology Normal University, Key Laboratory of Organic Chemistry, Jiangxi Province, Nanchang 330013, China; jinfengyancg@163.com (F.J.); tao_yang2019@yeah.net (T.Y.); songxr2015@163.com (X.-R.S.); mtbaijiang@yeah.net (J.B.); ouyangruchun@yeah.net (R.Y.); dinghaixin0204@yeah.net (H.D.)

**Keywords:** TMSBr, propargylic alcohols, azides, cascade cyclization, 4-bromo quinolines

## Abstract

Difficult-to-access 4-bromo quinolines are constructed directly from easily prepared *ortho*-propynol phenyl azides using TMSBr as acid-promoter. The cascade transformation performs smoothly to generate desired products in moderate to excellent yields with good functional groups compatibility. Notably, TMSBr not only acted as an acid-promoter to initiate the reaction, and also as a nucleophile. In addition, 4-bromo quinolines as key intermediates could further undergo the coupling reactions or nucleophilic reactions to provide a variety of functionalized compounds with molecular diversity at C4 position of quinolines.

## 1. Introduction

Quinolines are distinctive and significant frameworks which are widely existed in numerous pharmaceuticals, pesticide molecules, bioactive molecules, and natural products [1,2,3,4,5,6,7,8]. Moreover, such compounds using as ligands play crucial role in synthetic and catalysis chemistry [9,10,11,12,13]. Consequently, developing general and flexible approach towards these heterocycles has attracted much attention among synthetic chemists. Until now, despite significant achievements having been made in the construction of functionalized quinolines [14,15,16,17,18,19,20,21,22], methods for the direct synthesis of 4-halo quinolines are still limited [23,24,25,26]. 4-halo quinolines have been widely used as key synthetic intermediates for the construction of various bioactive molecules or drugs [27,28,29]. Therefore, the development of an efficient and versatile strategy towards 4-halo quinolines is highly desirable, especially through a cascade cyclization, because of the merits of efficiency and atomic economy.

**Scheme 1:**
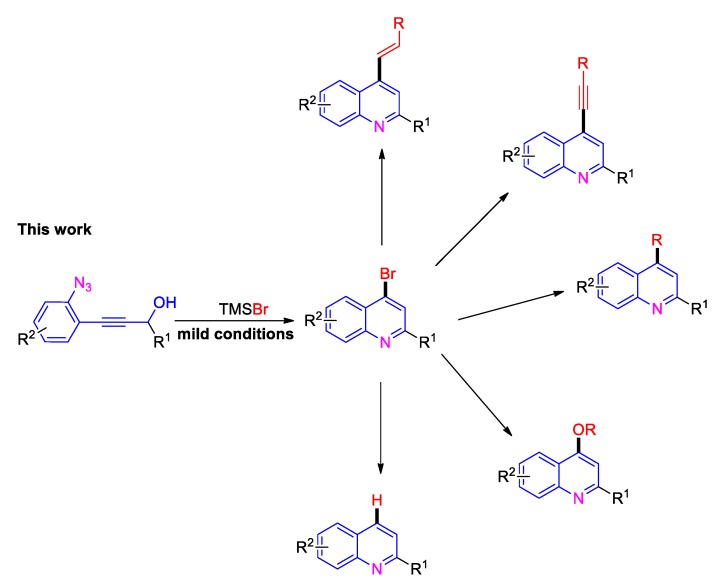
Our strategy for the construction of 4-bromo quinolines and its applications.

Based on its distinctive bifunctional group characteristics, the cascade reaction of propynols is an important tactic in organic synthesis, which exerts a significant role in the construction of functionalized carbo- or heterocyclic compounds [30,31,32,33,34]. In the past few years, our group had developed various efficient methods to construct functionalized heterocyclics through the cascade cyclization of propargylic alcohols in the presence of acid-promoter [35,36,37,38,39,40,41,42,43,44]. For example, we recently reported an efficient approach for the construction of 4-chrolo quinolines via the cyclization of *ortho*-propynol phenyl azides with TMSCl as acid-promoter [45]. Taking into consideration that the coupling reaction of chloro-substituted compounds is more difficult than bromo- or iodo-substituted compounds, the further development of universal approach for the construction of 4-bromo quinolines is still desirable and necessary. Herein, we report a general TMSBr-promoted the cascade cyclization of *ortho*-propynol phenyl azides for constructing 4-bromo quinolines, which can further undergo the coupling reactions or nucleophilic reactions to provide a variety of functionalized compounds with molecular diversity at C4 position of quinolines (Scheme 1). Compared to the Shvartsberg’s method [26], our developed strategy has the merits of good functional groups compatibility, easy preparation of the starting material, and simple operation.

## 2. Results and Discussion

Initially, the reaction conditions were optimized for cascade cyclization of *ortho*-propynol phenyl azides **1a** in the presence of TMSBr. Various solvents, temperatures, and TMSBr loading were investigated, and all cases were shown in Table 1. To our delight, with 2.5 equiv of TMSBr in different solvents—such as MeCN, CH_3_NO_2_, DCE, 1,4-dioxane, HOAc, and DCM—all reactions proceeded smoothly and cleanly to produce expected product 4-bromo-2-(4-methoxyphenyl)quinoline **2a** (Table 1, entries 1–5); CH_3_NO_2_ as solvent was most suitable for this transformation (73% yield). Encouraged by this preliminary result, further efforts were then directed toward improving the yield of desired product **2a** while suppressing the classical Meyer–Schuster rearrangement side reaction. Our studies on the loading of TMSBr with CH_3_NO_2_ as solvent showed that 3.5 equiv of TMSBr was the most efficient for this cascade transformation and could improve the yield of product **2a** to 81% (Table 1, entries 6–8). Subsequently, the examination of the reaction temperature indicated that the choice of reaction temperature was also an important in this transformation (entries 9, 10). Furthermore, no better yield was obtained when hydrobromic acid (HBr, 48 wt % in H_2_O) was used instead of TMSBr as the acid promoter (entry 11). Therefore, we establish the reaction conditions as optimum: 0.2 mmol of 2-propynol phenyl azides, 3.5 equiv of TMSBr in CH_3_NO_2_ were stirred at 60 °C.

**Figure 1:**
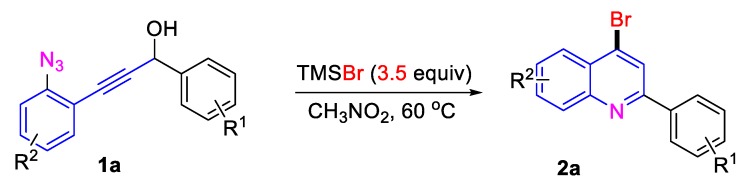

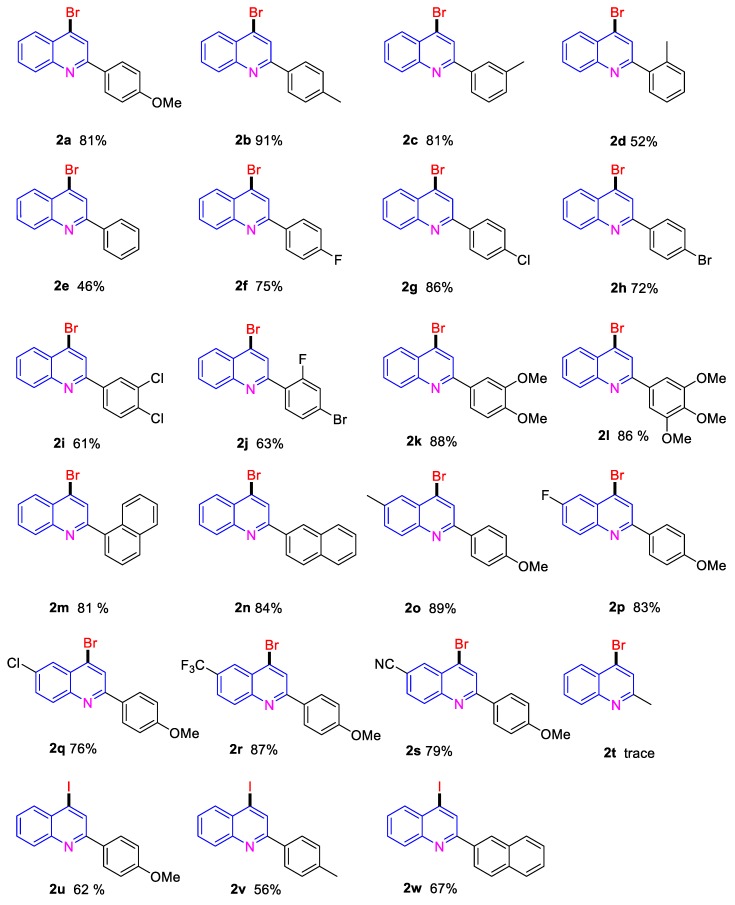
Transformation of *ortho*-propynol phenyl azides **1** to 4-bromo quinolines **2** ^a^. ^a^ Unless otherwise noted, all reactions were performed with **1** (0.2 mmol) in CH_3_NO_2_ (2.0 mL) at 60 °C for 1 h. Isolated yield.

Then, we investigated the generality of the reaction with diverse substituted propynols **1** using TMSBr as acid-promoter and nucleophile, and the results are presented in Figure 1. Various substituents R^1^ and R^2^ on the aryl ring were well-tolerated under the optimal conditions, efficiently generating the corresponding products 4-bromo quinolines in favorable yields (up to 91% yield). Firstly, we investigated the influence of substituent electronic effects on this reaction, and the results indicated that substrates containing electron-donor groups (OMe, Me) gave better transformation than those containing electron-poor groups (F, Cl, Br). This might due to the fact that the reaction involved the carbocation intermediate (Intermediate **B**, see Scheme 4); and the electron-rich groups were good for the stabilization of carbocation intermediate. The corresponding products 4-bromo quinolines give the better yields compared to the synthesis of 4-chrolo quinolines bearing the electron-withdrawing groups. Substrates bearing *ortho*-position substituent provided slightly lower yields (**2j**–**2k**), indicating that the steric effect showed clear influence on this reaction. Importantly, the functionalities of halogen atoms such as fluorine, chlorine, and bromine were also tolerated for this transformation producing the target products. Such halogenated products could be converted into a variety of functionalized quinolines through cross-coupling reactions. Substrates containing two or three substituents attached to the benzene ring smoothly, and the target compounds were generated in good to excellent yields. Notably, the substrates with naphthyl or styryl group (**1m** and **1o**) were also compatible to generate the target products in good yields (**2k**–**2m**). Then we examined the effect of a substituent (R^2^) on another aromatic ring on this transformation. Both electron-rich and electron-poor substituents were performed smoothly to produce the target compounds in 76–89% yields (**2m**–**2s**). It was noteworthy that the strong electron-deficient groups (CN and CF_3_) in R^2^ also proceeded well in this reaction and provided the target products in good yields. Unfortunately, no target product **2t** was generated when alkyl-substituted substrate **1t** was performed under the optimal conditions. Having successfully accomplished the direct formation of 4-bromo-quinolines, this cascade reaction was further extended to the construction of 4-iodo quinolines by using 2-propynol phenyl azides as starting materials with TMSI in CH_3_NO_2_ at 60 °C for 1.0 h under these circumstances. Some selected substrates (**1a**, **1b**, **1n**) were tolerated smoothly to the corresponding 4-iodo quinolines in moderate yields. 

**Scheme 2:**
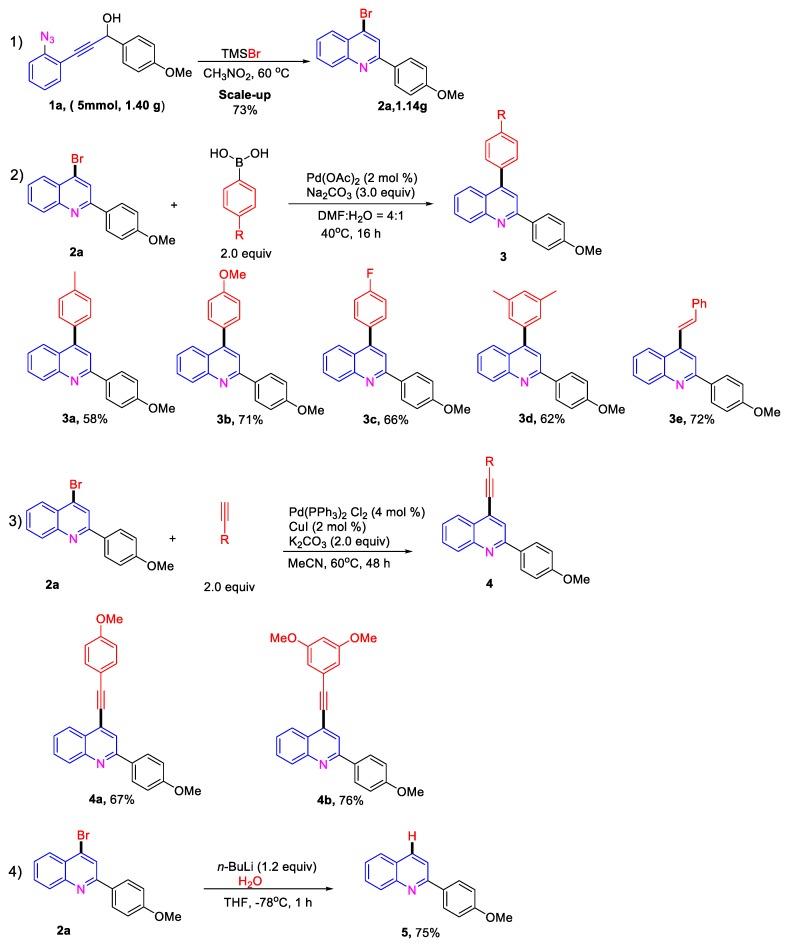
Functionality elaboration of 4-bromo-quinolines.

**Scheme 3:**
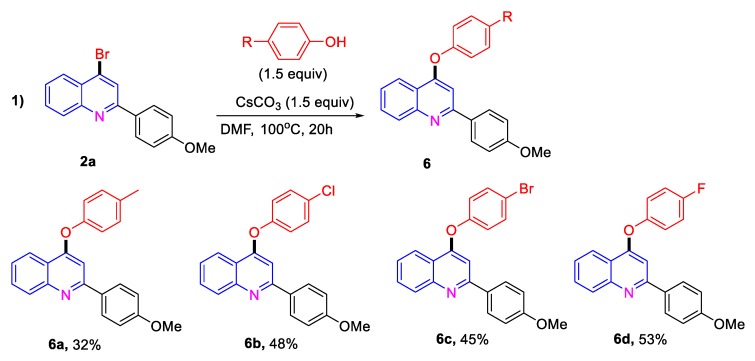
Transformation of 4-bromo quinoline **2a** to 4-aryloxy quinolines **6**.

**Scheme 4:**
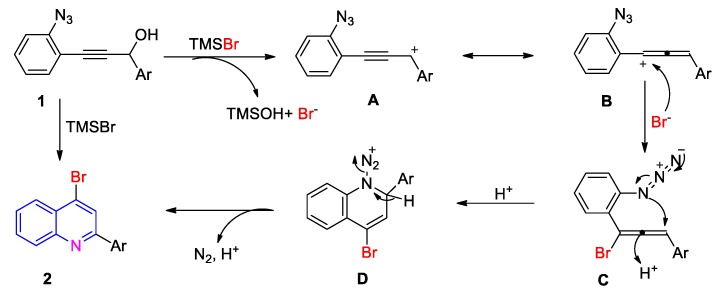
Proposed reaction mechanism.

Furthermore, the synthetic utility of this TMSBr-promoted reaction of *ortho*-propynol azides was demonstrated by a gram-scale synthesis (Scheme 2-1). The yield of product **2a** was not obvious affected when a gram-scale (5 mmol, 1.40g) experiment of **1a** was performed under similar reaction conditions. Importantly, a bromine atom at the 4-position of obtained product quinolines moiety is useful and easily substituted by various functional hydrocarbon and heteroatomic groups, which persuades us to exploit synthetic transformation of 4-bromo quinolones [46,47,48]. As representative examples, the Suzuki coupling reaction of **2a** with arylboronic acids to 4-aryl quinolines **3a**–**3d** in good yield was achieved (Scheme 2-2) [46]. Notably, the corresponding product 4-vinyl quinoline **3e** was also generated when the reaction of **2a** with *E*-phenylethenylboronic acid. Furthermore, the Sonogashira coupling of **2a** with arylacetylene could smoothly proceed to produce the target products **4a**–**4b** in good yields (Scheme 2-3) [47]. More importantly, the classical reduction reaction of **2a** to the corresponding quinoline **5** was also investigated (Scheme 2-4). These results clearly demonstrate the usefulness of our obtained product 4-bromo quinolines as synthetic intermediates. 

As we all known, 4-aryloxy quinolines are significant structure frameworks which are existed widely in various bioactive molecules and natural products [49,50,51,52]. In this context, the synthesis of 4-aryloxy quinolines from 4-bromo quinolines is attractive because of the clean conversion and the mild reaction conditions. Therefore, the scope of the reactions was also investigated by varying the phenols. Some representative substituted 4-aryllkoxy quinolines **6a**–**6d** were generated in acceptable yields by choosing the appropriate nucleophilic reagents (Scheme 3). 

On the basis of the above experimental results and literature reports [45,53,54], we propose a plausible reaction mechanism for this reaction (Scheme 4). Firstly, a proargylic carbocation intermediate **A** was formed through the TMSBr-promoted the dehydration of propargylic alcohols **1**. Intermediate **A** could easily undergo tautomerization to generate allenic carbocation intermediate **B**, which could be attracted by nucleophile halide anion (Br^−^) to produce intermediate **C**. Subsequently, the 6-endo-trig cyclization of intermediate **C** in the presence of proton forms intermediate **D**. Finally, the target product **2** was generated through the aromatization of the intermediate **D** with the generation of a nitrogen gas and a proton. 

## 3. Materials and Methods

### 3.1. General Remarks

^1^H-NMR spectra were recorded on 400 MHz in CDCl_3_ and ^13^C-NMR spectra were recorded on 100 MHz in CDCl_3_. Chemical shifts (ppm) were recorded with tetramethylsilane (TMS) as the internal reference standard. Multiplicities are given as: s (singlet), d (doublet), t (triplet), dd (doublet of doublets), q (quartet), or m (multiplet). High-resolution mass spectrometry (HRMS) was performed on a TOF/Q–TOF mass spectrometer. Copies of the ^1^H-NMR and ^13^C-NMR spectra are provided in the Supporting Information. Commercially available reagents were used without further purification. All solvents were dried under standard method.

### 3.2. General Procedure for the Construction of 4-Bromo Quinolines **2**

To a seal tube was added *ortho*-propynol phenyl azides (**1**) (0.2 mmol), TMSBr (0.7 mmol), in CH_3_NO_2_ at 60 °C. After 1.0 h, as monitored by TLC, the reaction mixture was concentrated in vacuum and purified by column chromatography to generate 4-bromo quinolines **2**.

4-Bromo-2-(4-Methoxyphenyl)quinoline (**2a**)

The title compound was prepared according to the 0.5, 130.8, 134.5, 148.7, 156.7, 161.1. general procedure and purified by column chromatography (silica gel, petroleum ether/ethyl acetate) to give a product **2a** (81%) [45]. ^1^H-NMR (400 MHz, CDCl_3_): δ 3. 79 (s, 3 H), 6.95 (dd, *J* = 2.0, 6.8 Hz, 2 H), 7.47–7.51 (m, 1 H), 7.63–7.67 (m, 1 H), 8.01–8.07 (m, 5 H). ^13^C-NMR (100 MHz, CDCl_3_): δ 55.4, 122.4, 126.3, 126.5, 127.0, 128.9, 129.8, 13.

4-Bromo-2-(p-tolyl)quinoline (**2b**)

The title compound was prepared according to the general procedure and purified by column chromatography (silica gel, petroleum ether/ethyl acetate) to give a product **2b** (91%). ^1^H-NMR (400 MHz, CDCl_3_): δ 2.34 (s, 3 H), 7.23 (d, *J* = 8.4 Hz, 2 H), 7.48–7.52 (m, 1 H), 7.64–7.68 (m, 1 H), 7.95 (d, *J* = 8.0 Hz, 2 H), 8.04–8.08 (m, 3 H). ^13^C-NMR (100 MHz, CDCl_3_): δ 21.3, 122.7, 126.5, 127.2, 127.4, 129.6, 130.0, 130.4, 134.5, 135.5, 139.9, 148.7, 157.1. HRMS (ESI, *m*/*z*): calcd for C_16_H_12_BrN: M + H = 298.0226; found: 298.0229.

4-Bromo-2-(m-tolyl)quinoline (**2c**)

The title compound was prepared according to the general procedure and purified by column chromatography (silica gel, petroleum ether/ethyl acetate) to give a product **2c** (81%). ^1^H-NMR (400 MHz, CDCl_3_): δ 2.39 (s, 3 H), 7.21 (d, *J* = 7.2 Hz, 1 H), 7.33 (t, *J* = 7.6 Hz, 1 H), 7.52 (t, *J* = 7.2 Hz, 1 H), 7.68 (t, *J* = 7.6 Hz, 1 H), 7.81 (d, *J* = 8.0 Hz, 1 H), 7.89 (s, 1 H), 8.07–8.10 (m, 3 H). ^13^C-NMR (100 MHz, CDCl_3_): δ 21.5, 123.0, 124.6, 126.5, 126.6, 127.4, 128.2, 128.8, 130.0, 130.5, 130.6, 134.5, 138.3, 138.6, 148.7, 157.4. HRMS (ESI, *m*/*z*): calcd for C_16_H_12_BrN: M + H = 298.0226; found: 298.0229.

4-Bromo-2-(o-tolyl)quinoline (**2d**)

The title compound was prepared according to the general procedure and purified by column chromatography (silica gel, petroleum ether/ethyl acetate) to give a product **2d** (52%). ^1^H-NMR (400 MHz, CDCl_3_): δ 2.35 (s, 3 H), 7.20–7.29 (m, 3 H), 7.41 (d, *J* = 6.8 Hz, 1 H), 7.58 (t, *J* = 7.6 Hz, 1 H), 7.70 (t, *J* = 7.6 Hz, 1 H), 7.78 (s, 1 H), 8.06–8.16 (m, 2 H). ^13^C-NMR (100 MHz, CDCl_3_): δ 20.3, 126.1, 126.1, 126.3, 126.6, 127.6, 128.9, 129.6, 130.0, 130.5, 131.1, 133.9, 136.1, 139.4, 148.4, 160.0. HRMS (ESI, *m*/*z*): calcd for C_16_H_12_BrN: M + H = 298.0226; found: 298.0227. 

4-Bromo-2-phenylquinoline (**2e**)

The title compound was prepared according to the general procedure and purified by column chromatography (silica gel, petroleum ether/ethyl acetate) to give a product **2e** (46%). ^1^H-NMR (400 MHz, CDCl_3_): δ 7.40–7.48 (m, 3 H), 7.54–7.56 (m, 1 H), 7.67–7.71 (m, 1 H), 8.05–8.11 (m, 5 H). ^13^C-NMR (100 MHz, CDCl_3_): δ 122.9, 126.5, 126.7, 127.5, 127.5, 128.9, 129.8, 130.1, 130.5, 134.6, 138.4, 148.8, 157.2. HRMS (ESI, *m*/*z*): calcd for C_15_H_10_BrN: M + H = 284.0069; found: 284.0071. 

4-Bromo-2-(4-fluorophenyl)quinoline (**2f**)

The title compound was prepared according to the general procedure and purified by column chromatography (silica gel, petroleum ether/ethyl acetate) to give a product **2f** (75%). ^1^H-NMR (400 MHz, CDCl_3_): δ 7.11–7.18 (m, 2 H), 7.52–7.56 (m, 1 H), 7.67–7.71 (m, 1 H), 8.05–8.11 (m, 5 H). ^13^C-NMR (100 MHz, CDCl_3_): δ 115.8, 116.0, 122.5, 126.6, 127.5, 129.4, 129.5, 130.0, 130.7, 134.5, 134.8, 148.7, 156.0, 162.8, 165.3. HRMS (ESI, *m*/*z*): calcd for C_15_H_9_BrFN: M + H = 301.9975; found: 301.9973.

4-Bromo-2-(4-chlorophenyl)quinoline (**2g**)

The title compound was prepared according to the general procedure and purified by column chromatography (silica gel, petroleum ether/ethyl acetate) to give a product **2g** (86%). ^1^H-NMR (400 MHz, CDCl_3_): δ 7.41 (d, *J* = 8.4 Hz, 2 H), 7.52–7.56 (m, 1 H), 7.67–7.71 (m, 1 H), 7.99–8.10 (m, 5 H). ^13^C-NMR (100 MHz, CDCl_3_): δ 122.5, 126.6, 126.7, 127.7, 128.7, 129.1, 130.0, 130.7, 134.8, 136.0, 136.7, 148.7, 155.8. HRMS (ESI, *m*/*z*): calcd for C_15_H_9_BrClN: M + H = 317.9680; found: 317.9682. 

4-Bromo-2-(4-bromophenyl)quinoline (**2h**)

The title compound was prepared according to the general procedure and purified by column chromatography (silica gel, petroleum ether/ethyl acetate) to give a product **2h** (72%). ^1^H-NMR (400 MHz, CDCl_3_): δ 7.54–7.57 (m, 3 H), 7.68 (t, *J* = 8.4 Hz, 1 H), 7.94 (d, *J* = 8.4 Hz, 2 H), 8.04–8.10 (m, 3 H). ^13^C-NMR (100 MHz, CDCl_3_): δ 122.4, 124.5, 126.6, 126.7, 127.7, 129.0, 130.1, 130.7, 132.0, 134.8, 137.2, 148.7, 155.9. HRMS (ESI, *m*/*z*): calcd for C_15_H_9_Br_2_N: M + H = 361.9175; found: 361.9179. 

4-Bromo-2-(3,4-dichlorophenyl)quinoline (**2i**)

The title compound was prepared according to the general procedure and purified by column chromatography (silica gel, petroleum ether/ethyl acetate) to give a product **2i** (61%). ^1^H-NMR (400 MHz, CDCl_3_): δ 7.50 (d, *J* = 8.4 Hz, 1 H), 7.54–7.58 (m, 1 H), 7.69–7.73 (m, 1 H), 7.87–7.90 (m, 1 H), 8.02–8.11 (m, 3 H), 8.21 (d, *J* = 2.0 Hz, 1 H). ^13^C-NMR (100 MHz, CDCl_3_): δ 122.2, 126.4, 126.6, 126.8, 128.0, 129.3, 130.1, 130.8, 130.9, 133.3, 134.1, 135.0, 138.1, 148.6, 154.4. HRMS (ESI, *m*/*z*): calcd for C_15_H_8_BrCl_2_N: M + H = 351.9290; found: 351.9291.

4-Bromo-2-(4-bromo-2-fluorophenyl)quinoline (**2j**)

The title compound was prepared according to the general procedure and purified by column chromatography (silica gel, petroleum ether/ethyl acetate) to give a product **2j** (63%). ^1^H-NMR (400 MHz, CDCl_3_): δ 7.34 (dd, *J* = 1.6, 6.8 Hz, 1 H), 7.40 (dd, *J* = 1.6, 8.4 Hz, 1 H) 7.59 (t, *J* = 7.2 Hz, 1 H), 7.72 (t, *J* = 7.2 Hz, 1 H), 7.97 (t, *J* = 7.6 Hz, 1 H), 8.07–8.14 (m, 3 H). ^13^C-NMR (100 MHz, CDCl_3_): δ 119.8, 120.0, 124.1, 124.2, 125.8, 125.9, 126.7, 126.9, 128.1, 128.2, 128.2, 130.0, 130.7, 132.5, 132.6, 134.4, 148.6, 152.6, 159.1, 161.8. HRMS (ESI, *m*/*z*): calcd for C_15_H_8_Br_2_FN: M + H = 379.9080; found: 379.9084.

4-Bromo-2-(3,4-dimethoxyphenyl)quinoline (**2k**)

The title compound was prepared according to the general procedure and purified by column chromatography (silica gel, petroleum ether/ethyl acetate) to give a product **2k** (88%). ^1^H-NMR (400 MHz, CDCl_3_): δ 3.87 (s, 3 H), 3.97 (s, 3 H), 6.89 (d, *J* = 8.4 Hz, 1 H), 7.48–7.56 (m, 2 H), 7.64–7.68 (m, 1 H), 7.75 (d, *J* = 2.0 Hz, 1 H), 8.05–8.08 (m, 3 H). ^13^C-NMR (100 MHz, CDCl_3_): δ 55.9, 56.0, 110.2, 111.0, 120.3, 122.5, 126.4, 126.5, 127.1, 129.8, 130.5, 131.0, 134.5, 148.6, 149.4, 150.7, 156.6. HRMS (ESI, *m*/*z*): calcd for C_17_H_14_BrNO_2_: M + H = 344.0281; found: 344.0283. 

4-Bromo-2-(3,4,5-trimethoxyphenyl)quinoline (**2l**)

The title compound was prepared according to the general procedure and purified by column chromatography (silica gel, petroleum ether/ethyl acetate) to give a product **2l** (86%). ^1^H-NMR (400 MHz, CDCl_3_): δ 3.85 (s, 3 H), 3.93 (s, 6 H), 7.29 (s, 2 H), 7.53 (t, *J* = 7.2 Hz, 1 H), 7.69 (t, *J* = 7.6 Hz, 1 H), 8.04 (s, 1 H), 8.07–8.10 (m, 2 H). ^13^C-NMR (100 MHz, CDCl_3_): δ 56.3, 60.9, 104.8, 122.7, 126.5, 127.5, 129.9, 130.6, 133.8, 134.6, 139.8, 148.5, 153.6, 156.7. HRMS (ESI, *m*/*z*): calcd for C_18_H_16_BrNO_3_: M + H = 374.0386; found: 374.0382. 

4-Bromo-2-(naphthalen-1-yl)quinoline (**2m**)

The title compound was prepared according to the general procedure and purified by column chromatography (silica gel, petroleum ether/ethyl acetate) to give a product **2m** (81%). ^1^H-NMR (400 MHz, CDCl_3_): δ 7.41–7.48 (m, 2 H), 7.51–7.55 (m, 1 H), 7.61–7.66 (m, 2 H), 7.56 (t, *J* = 7.2 Hz, 1 H), 7.86–7.91 (m, 2 H), 7.96 (s, 1 H), 8.05 (d, *J* = 8.0 Hz, 1 H), 8.19 (dd, *J* = 8.8 Hz, 2 H). ^13^C-NMR (100 MHz, CDCl_3_): δ 125.3, 126.1, 126.6, 126.7, 126.9, 127.0, 127.9, 128.0, 128.5, 129.6, 129.9, 130.8, 131.0, 133.9, 134.4, 137.1, 148.4, 159.1. HRMS (ESI, *m*/*z*): calcd for C_19_H_12_BrN: M + H = 334.0226; found: 334.0227. 

4-Bromo-2-(naphthalen-2-yl)quinoline (**2n**)

The title compound was prepared according to the general procedure and purified by column chromatography (silica gel, petroleum ether/ethyl acetate) to give a product **2n** (84%). ^1^H-NMR (400 MHz, CDCl_3_): δ 7.42–7.46 (m, 2 H), 7.50–7.54 (m, 1 H), 7.66–7.70 (m, 1 H), 7.78–7.81 (m, 1 H), 7.88 (dd, *J* = 2.8, 5.6 Hz, 2 H), 8.08–8.11 (m, 2 H), 8.22–8.24 (m, 2 H), 8.47 (d, *J* = 0.8 Hz, 1 H). ^13^C-NMR (100 MHz, CDCl_3_): δ 123.0, 124.7, 126.4, 126.6, 126.7, 126.9, 127.3, 127.5, 127.7, 128.7, 128.8, 130.1, 130.6, 133.3, 134.0, 134.6, 135.6, 148.8, 156.9. HRMS (ESI, *m*/*z*): calcd for C_19_H_12_BrN: M + H = 334.0226; found: 334.0227.

4-Bromo-2-(4-methoxyphenyl)-6-methylquinoline (**2o**)

The title compound was prepared according to the general procedure and purified by column chromatography (silica gel, petroleum ether/ethyl acetate) to give a product **2o** (89%). ^1^H-NMR (400 MHz, CDCl_3_): δ 2.48 (s, 3 H), 3.79 (s, 3 H), 6.94 (d, *J* = 8.8 Hz, 2 H), 7.47 (dd, *J* = 1.6, 8.4 Hz, 1 H), 7.80 (s, 1 H), 7.91 (d, *J* = 8.8 Hz, 1 H), 7.98–8.00 (m, 3 H). ^13^C-NMR (100 MHz, CDCl_3_): δ 21.7, 55.3, 114.2, 122.4, 125.3, 126.2, 128.7, 129.6, 131.1, 132.6, 133.7, 137.2, 147.3, 155.8, 160.9. HRMS (ESI, *m*/*z*): calcd for C_17_H_14_BrNO: M + H = 328.0332; found: 328.0331.

4-Bromo-6-fluoro-2-(4-methoxyphenyl)quinoline (**2p**)

The title compound was prepared according to the general procedure and purified by column chromatography (silica gel, petroleum ether/ethyl acetate) to give a product **2p** (83%). ^1^H-NMR (400 MHz, CDCl_3_): δ 3.81 (s, 3 H), 6.95 (d, *J* = 8.4 Hz, 2 H), 7.39–7.44 (m, 1 H), 7.70 (dd, *J* = 2.8, 9.6 Hz, 1 H), 7.99–8.05 (m, 4 H). ^13^C-NMR (100 MHz, CDCl_3_): δ 55.4, 110.2, 110.4, 114.3, 120.5, 120.7, 123.0, 127.2, 127.3, 128.8, 130.6, 132.4, 132.5, 133.3, 133.4, 145.8, 156.1, 156.2, 159.7, 161.2, 162.2. HRMS (ESI, *m*/*z*): calcd for C_16_H_11_BrFNO: M + H = 332.0081; found: 332.0081. 

4-Bromo-6-chloro-2-(4-methoxyphenyl)quinoline (**2q**)

The title compound was prepared according to the general procedure and purified by column chromatography (silica gel, petroleum ether/ethyl acetate) to give a product **2q** (76%). ^1^H-NMR (400 MHz, CDCl_3_): δ 3.80 (s, 3 H), 6.95 (d, *J* = 8.8 Hz, 2 H), 7.57 (dd, *J* = 2.4, 8.8 Hz, 1 H), 7.94–8.05 (m, 5 H). ^13^C-NMR (100 MHz, CDCl_3_): δ 55.4, 114.3, 123.1, 125.5, 127.0, 128.8, 130.4, 131.4, 131.5, 133.0, 133.1, 147.1, 156.9, 161.3. HRMS (ESI, *m*/*z*): calcd for C_16_H_11_BrClNO: M + H = 347.9785; found: 347.9787.

4-Bromo-2-(4-methoxyphenyl)-6-(trifluoromethyl)quinoline (**2**r)

The title compound was prepared according to the general procedure and purified by column chromatography (silica gel, petroleum ether/ethyl acetate) to give a product **2r** (87%). ^1^H-NMR (400 MHz, CDCl_3_): δ 3.80 (s, 3 H), 6.94 (d, *J* = 8.8 Hz, 2 H), 7.79 (d, *J* = 8.8 Hz, 1 H), 8.03 (d, *J* = 8.4 Hz, 2 H), 8.10 (d, *J* = 8.4 Hz, 2 H), 8.34 (s, 1 H). ^13^C-NMR (100 MHz, CDCl_3_): δ 55.4, 114.4, 123.4, 124.6, 124.7, 125.5, 126.1, 126.1, 129.1, 130.0, 131.0, 135.0, 149.7, 158.6, 161.7. HRMS (ESI, *m*/*z*): calcd for C_17_H_11_BrF_3_NO: M + H = 382.0049; found: 382.0045.

4-Bromo-2-(4-methoxyphenyl)quinoline-6-carbonitrile (**2s**)

The title compound was prepared according to the general procedure and purified by column chromatography (silica gel, petroleum ether/ethyl acetate) to give a product **2s** (79%). ^1^H-NMR (400 MHz, CDCl_3_): δ 3.83 (s, 3 H), 6.98 (d, *J* = 8.8 Hz, 2 H), 7.79 (dd, *J* = 1.6, 8.4 Hz, 1 H), 8.08 (dd, *J* = 5.6, 8.8 Hz, 3 H), 8.16 (s, 1 H), 8.47 (d, *J* = 1.2 Hz, 1 H). ^13^C-NMR (100 MHz, CDCl_3_): δ 55.5, 110.4, 114.5, 118.5, 123.8, 126.0, 129.3, 129.7, 131.2, 131.3, 132.9, 134.5, 149.9, 159.4, 162.0. HRMS (ESI, *m*/*z*): calcd for C_17_H_11_BrN_2_O: M + H = 339.0128; found: 339.0128.

4-Iodo-2-(4-methoxyphenyl)quinoline (**2u**) 

The title compound was prepared according to the general procedure and purified by column chromatography (silica gel, petroleum ether/ethyl acetate) to give a product **2u** (62%) [45]. ^1^H-NMR (400 MHz, CDCl_3_): δ 3.89 (s, 3 H), 7.03–7.05 (m, 2 H), 7.54–7.58 (m, 1 H), 7.70–7.74 (m, 1 H), 7.98 (d, *J* = 8.4 Hz, 1 H), 8.05 (d, *J* = 8.4 Hz, 1 H), 8.09–8.12 (m, 2 H), 8.42 (s, 1 H). ^13^C-NMR (100 MHz, CDCl_3_): δ 66.4, 112.5, 114.3, 127.4, 128.9, 128.9, 130.1, 130.1, 130.5, 130.6, 131.4, 147.8, 156.7, 161.1. 

4-Iodo-2-(*p*-tolyl)quinoline (**2v**)

The title compound was prepared according to the general procedure and purified by column chromatography (silica gel, petroleum ether/ethyl acetate) to give a product **2v** (56%). ^1^H-NMR (400 MHz, CDCl_3_): 2.44 (s, 3 H), 7.33 (d, *J* = 8.0 Hz, 2 H), 7.60 (t, *J* = 7.2 Hz, 1 H), 7.72–7.76 (m, 1 H), 7.99–8.08 (m, 4 H), 8.45 (s, 1 H). ^13^C-NMR (100 MHz, CDCl_3_): δ 21.3, 112.5, 127.4, 127.6, 129.0, 129.7, 130.2, 130.4, 130.5, 131.4, 135.2, 139.9, 147.8, 157.0. HRMS (ESI, *m*/*z*): calcd for C_16_H_12_IN: M + H = 346.0087; found: 346.0092. 

4-Iodo-2-(naphthalen-2-yl)quinoline (**2w**)

The title compound was prepared according to the general procedure and purified by column chromatography (silica gel, petroleum ether/ethyl acetate) to give a product **2w** (67%). ^1^H-NMR (400 MHz, CDCl_3_): δ 7.54–7.57 (m, 2 H), 7.60–7.64 (m, 1 H), 7.75–7.80 (m, 1 H), 7.90–7.92 (m, 1 H), 7.99–8.05 (m, 3 H), 8.14 (d, *J* = 8.4 Hz, 1 H), 8.32–8.35 (m, 1 H), 8.59 (s, 1 H), 8.63 (s, 1 H). ^13^C-NMR (100 MHz, CDCl_3_): δ 112.6, 124.8, 126.5, 126.9, 127.3, 127.7, 127.9, 128.7, 128.8, 129.2, 130.3, 130.6, 130.7, 131.5, 133.4, 134.0, 135.3, 147.9, 156.9. HRMS (ESI, *m*/*z*): calcd for C_19_H_12_IN: M + H = 382.0087; found: 382.0089.

2-(4-Methoxyphenyl)-4-(p-tolyl)quinoline (**3a**) 

The title compound was purified by column chromatography (silica gel, petroleum ether/ethyl acetate) to give a product **3a** (58%) [55]. ^1^H-NMR (400 MHz, CDCl_3_): δ 2.38 (s, 3 H), 3.78 (s, 3 H), 6.95 (d, *J* = 8.4 Hz, 2 H), 7.26 (d, *J* = 8.0 Hz, 2 H), 7.32–7.37 (m, 3 H), 7.59–7.63 (m, 1 H), 7.66 (s, 1 H), 7.81 (d, *J* = 8.4 Hz, 1 H), 8.05–8.12 (m, 3 H). ^13^C-NMR (100 MHz, CDCl_3_): δ 21.3, 55.3, 114.2, 118.8, 125.6, 125.7, 125.8, 128.9, 129.2, 129.3, 129.4, 129.8, 132.3, 135.6, 138.2, 148.8, 149.0, 156.4, 160.8.

2,4-bis(4-Methoxyphenyl)quinoline (**3b**) 

The title compound was purified by column chromatography (silica gel, petroleum ether/ethyl acetate) to give a product **3b** (71%) [55]. ^1^H-NMR (400 MHz, CDCl_3_): δ 3.79 (s, 3 H), 3.81 (s, 3 H), 6.97 (dd, *J* = 8.8, 13.2 Hz, 4 H), 7.33–7.37 (m, 1 H), 7.41 (d, *J* = 8.8 Hz, 2 H), 7.59–7.63 (m, 1 H), 7.66 (s, 1 H), 7.82 (d, *J* = 8.4 Hz, 1 H), 8.06–8.12 (m, 3 H). ^13^C-NMR (100 MHz, CDCl_3_): δ 55.3, 55.4, 114.0, 114.2, 118.8, 125.6, 125.7, 125.8, 128.9, 129.3, 129.8, 130.7, 132.3, 148.6, 148.9, 156.4, 159.8, 160.8.

4-(4-Fluorophenyl)-2-(4-methoxyphenyl)quinoline (**3c**)

The title compound was purified by column chromatography (silica gel, petroleum ether/ethyl acetate) to give a product **3c** (66%) [55]. ^1^H-NMR (400 MHz, CDCl_3_): δ 3.80 (s, 3 H), 6.96 (d, *J* = 8.8 Hz, 2 H), 7.13–7.18 (m, 2 H), 7.35–7.39 (m, 1 H), 7.43–7.46 (m, 2 H), 7.61–7.65 (m, 2 H), 7.73 (d, *J* = 8.4 Hz, 1 H), 8.06–8.13 (m, 3 H). ^13^C-NMR (100 MHz, CDCl_3_): δ 55.4, 114.2, 115.5, 115.7, 118.9, 125.3, 125.5, 126.0, 128.9, 128.5, 130.0, 131.2, 131.3, 132.0, 134.4, 134.5, 147.9, 148.8, 156.4, 160.9, 161.6, 164.1.

4-(3,5-Dimethylphenyl)-2-(4-methoxyphenyl)quinoline (**3d**)

The title compound was purified by column chromatography (silica gel, petroleum ether/ethyl acetate) to give a product **3d** (62%). ^1^H-NMR (400 MHz, CDCl_3_): δ 2.34 (s, 6 H), 3.79 (s, 3 H), 6.95 (d, *J* = 8.8 Hz, 2 H), 7.05–7.08 (m, 3 H), 7.33–7.37 (m, 1 H), 7.59–7.63 (m, 1 H), 7.67 (s, 1 H), 7.81 (d, *J* = 8.4 Hz, 1 H), 8.06–8.12 (m, 3 H). ^13^C-NMR (100 MHz, CDCl_3_): δ 21.3, 55.3, 114.2, 118.7, 125.6, 125.7, 125.8, 127.3, 128.9, 129.3, 129.8, 129.9, 132.2, 138.1, 138.4, 148.7, 149.3, 156.4, 160.8. HRMS (ESI, *m*/*z*): calcd for: C_24_H_21_NO: M + H = 340.1696; found: M + H = 340.1692.

(*E*)-2-(4-Methoxyphenyl)-4-styrylquinoline (**3e**)

The title compound was purified by column chromatography (silica gel, petroleum ether/ethyl acetate) to give a product **3e** (72%) [56]. ^1^H-NMR (400 MHz, CDCl_3_): δ 3.83 (s, 3 H), 6.99 (d, *J* = 8.4 Hz, 2 H), 7.27–7.39 (m, 4 H), 7.44–7.49 (m, 1 H), 7.58 (d, *J* = 7.2 Hz, 2 H), 7.63–7.67 (m, 1 H), 7.77 (d, *J* = 16.0 Hz, 1 H), 7.95 (s, 1 H), 8.08–8.12 (m, 4 H). ^13^C-NMR (100 MHz, CDCl_3_): δ 55.4, 114.2, 114.7, 123.3, 123.6, 125.2, 125.9, 127.1, 128.7, 128.8, 128.9, 129.4, 130.2, 132.4, 134.9, 136.7, 143.5, 148.8, 156.8, 160.8. 

2-(4-Methoxyphenyl)-4-((4-methoxyphenyl)ethynyl)quinoline (**4a**)

The title compound was purified by column chromatography (silica gel, petroleum ether/ethyl acetate) to give a product **4a** (67%) [45]. ^1^H-NMR (400 MHz, CDCl_3_): δ 3.86 (s, 3 H), 3.89 (s, 3 H), 6.95 (d, *J* = 8.8 Hz, 2 H), 7.05 (d, *J* = 8.8 Hz, 2 H), 7.55–7.59 (m, 1 H), 7.63 (d, *J* = 8.8 Hz, 2 H), 7.71–7.75 (m, 1 H), 8.00 (s, 1 H), 8.13–8.16 (m, 3 H), 8.33 (d, *J* = 7.6 Hz, 1 H). ^13^C-NMR (100 MHz, CDCl_3_): δ 55.4, 84.5, 98.2, 114.2, 114.2, 114.4, 120.9, 125.7, 126.3, 126.3, 128.8, 129.8, 129.9, 130.5, 131.8, 133.5, 148.2, 156.4, 160.4, 160.9.

4-((3,5-Dimethoxyphenyl)ethynyl)-2-(4-methoxyphenyl)quinoline (**4b**)

The title compound was purified by column chromatography (silica gel, petroleum ether/ethyl acetate) to give a product **4b** (76%). ^1^H-NMR (400 MHz, CDCl_3_): δ 3.78 (s, 6 H), 3.82 (s, 3 H), 6.48 (t, *J* = 2.0 Hz, 1 H), 6.76 (d, *J* = 2.4 Hz, 2 H), 6.98 (d, *J* = 8.8 Hz, 2 H), 7.51 (t, *J* = 7.6 Hz, 1 H), 7.67 (t, *J* = 8.0 Hz, 1 H), 7.96 (s, 1 H), 8.07–8.10 (m, 3 H), 8.25 (d, *J* = 8.0 Hz, 1 H). ^13^C-NMR (100 MHz, CDCl_3_): δ 55.5, 85.0, 97.8, 102.6, 109.7, 114.3, 121.3, 123.6, 125.6, 126.3, 126.5, 128.8, 129.9, 130.0, 130.0, 131.7, 148.2, 156.4, 160.7, 161.0. HRMS (ESI, *m*/*z*): calcd for: C_26_H_21_NO_3_: M + H = 396.1594; found: 396.1596.

2-(4-Methoxyphenyl)quinoline (**5**) 

The title compound was purified by column chromatography (silica gel, petroleum ether/ethyl acetate) to give a product **5** (75%) [57]. ^1^H-NMR (400 MHz, CDCl_3_): δ 3.79 (s, 3 H), 6.96 (d, *J* = 8.8 Hz, 2 H), 7.38–7.42 (m, 1 H), 7.59–7.64 (m, 1 H), 7.70–7.75 (m, 2 H), 8.04–8.09 (m, 4 H). ^13^C-NMR (100 MHz, CDCl_3_): δ 55.4, 114.2, 118.5, 125.8, 126.9, 127.4, 128.9, 129.5, 129.5, 132.2, 136.6, 148.3, 156.9, 160.8. 

2-(4-Methoxyphenyl)-4-(p-tolyloxy)quinoline (**6a**)

The title compound was purified by column chromatography (silica gel, petroleum ether/ethyl acetate) to give a product **6a** (32%). ^1^H-NMR (400 MHz, CDCl_3_): δ 2.35 (s, 3 H), 3.77 (s, 3 H), 6.88–6.90 (m, 3 H), 7.04 (d, *J* = 8.4 Hz, 2 H), 7.18–7.21 (m, 2 H), 7.44 (t, *J* = 7.6 Hz, 1 H), 7.66 (t, *J* = 7.6 Hz, 1 H), 7.84 (d, *J* = 8.8 Hz, 2 H), 8.04 (d, *J* = 8.8 Hz, 1 H), 8.25 (d, *J* = 8.0 Hz, 1 H). ^13^C-NMR (100 MHz, CDCl_3_): δ 20.9, 55.4, 101.7, 114.0, 120.4, 120.7, 121.7, 125.4, 128.8, 129.1, 130.2, 130.7, 132.5, 135.1, 149.8, 152.3, 158.1, 160.7, 162.5. HRMS (ESI, *m*/*z*): calcd for: C_23_H_19_NO_2_: M + H = 342.1489; found: 342.1495.

4-(4-Chlorophenoxy)-2-(4-methoxyphenyl)quinoline (**6b**)

The title compound was purified by column chromatography (silica gel, petroleum ether/ethyl acetate) to give a product **6b** (48%). ^1^H-NMR (400 MHz, CDCl_3_): δ 3.77 (s, 3 H), 6.89–6.92 (m, 3 H), 7.09 (d, *J* = 8.8 Hz, 2 H), 7.36 (d, *J* = 9.2 Hz, 2 H), 7.45 (t, *J* = 7.2 Hz, 1 H), 7.65–7.70 (m, 1 H), 7.86 (d, *J* = 8.8 Hz, 2 H), 8.05 (d, *J* = 8.4 Hz, 1 H), 8.19 (d, *J* = 7.6 Hz, 1 H). ^13^C-NMR (100 MHz, CDCl_3_): δ 55.5, 110.4, 114.5, 118.5, 123.8, 126.0, 129.3, 129.7, 131.2, 131.3, 132.9, 134.5, 149.9, 159.4, 162.0. HRMS (ESI, *m*/*z*): calcd for: C_22_H_16_ClNO_2_: M + H = 362.0942; found: 362.0948.

4-(4-Bromophenoxy)-2-(4-methoxyphenyl)quinoline (**6c**)

The title compound was purified by column chromatography (silica gel, petroleum ether/ethyl acetate) to give a product **6c** (45%). ^1^H-NMR (400 MHz, CDCl_3_): δ 3.77 (s, 3 H), 6.89–6.93 (m, 3 H), 7.04 (d, *J* = 8.8 Hz, 2 H), 7.45 (t, *J* = 7.2 Hz, 1 H), 7.51 (d, *J* = 8.8 Hz, 2 H), 7.67 (t, *J* = 8.4 Hz, 1 H), 7.86 (d, *J* = 8.4 Hz, 2 H), 8.06 (d, *J* = 8.4 Hz, 1 H), 8.18 (d, *J* = 8.0Hz, 1 H). ^13^C-NMR (100 MHz, CDCl_3_): δ 55.4, 102.3, 114.1, 118.2, 120.2, 121.5, 122.5, 125.7, 128.8, 129.2, 130.4, 132.1, 133.3, 149.8, 153.9, 158.1, 160.9, 161.7. HRMS (ESI, *m*/*z*): calcd for: C_22_H_16_BrNO_2_: M + H = 406.0437; found: 406.0431.

4-(4-Fluorophenoxy)-2-(4-methoxyphenyl)quinoline (**6d**)

The title compound was purified by column chromatography (silica gel, petroleum ether/ethyl acetate) to give a product **6d** (53%). ^1^H-NMR (400 MHz, CDCl_3_): δ 3.77 (s, 3 H), 6.89–6.93 (m, 3 H), 7.04 (d, *J* = 8.8 Hz, 2 H), 7.45 (t, *J* = 7.2 Hz, 1 H), 7.51 (d, *J* = 8.8 Hz, 2 H), 7.67 (t, *J* = 8.4 Hz, 1 H), 7.86 (d, *J* = 8.4 Hz, 2 H), 8.06 (d, *J* = 8.4 Hz, 1 H), 8.18 (d, *J* = 8.0Hz, 1 H). ^13^C-NMR (100 MHz, CDCl_3_): δ 55.4, 102.3, 114.1, 118.2, 120.2, 121.5, 122.5, 125.7, 128.8, 129.2, 130.4, 132.1, 133.3, 149.8, 153.9, 158.1, 160.9, 161.7. HRMS (ESI, *m*/*z*): calcd for: C_22_H_16_FNO_2_: M + H = 346.1238; found: 346.1234.

## 4. Conclusions

In summary, we have developed an efficient and general approach for the synthesis of 4-bromo or 4-iodo quinolines through the TMSBr promoted the cascade cyclization of *ortho*-propynol phenyl azides. It is noteworthy that the obtained products 4-halo quinolines could be used as key intermediate for the construction of various bioactive molecules, natural products, and drugs. A variety of 4-halo quinolines were obtained in moderate to excellent yields under mild conditions. This process does not require the use of metal catalysts, additional oxidants; water and nitrogen gas are generated as the only side products. 

## Figures and Tables

**Table 1:**
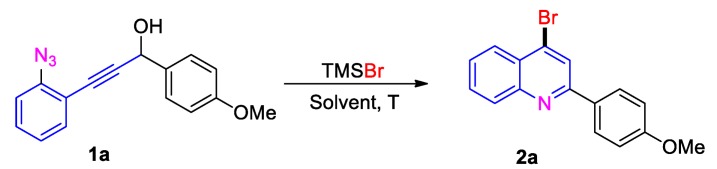
Optimization of the reaction for the synthesis of **2a** ^a^.

Entry	Solvent	TMSBr (x Equiv)	*T* [°C]	Yield [%]
1	DCE	2.5	60	45
2	MeCN	2.5	60	39
3	CH_2_Cl_2_	2.5	40	15
4	MeNO_2_	2.5	60	73
5	HOAc	2.5	60	36
6	MeNO_2_	3.5	60	81
7	MeNO_2_	3.0	60	78
8	MeNO_2_	2.0	60	67
9	MeNO_2_	3.5	80	82
10	MeNO_2_	3.5	rt	69
11 ^b^	MeNO_2_	3.5	60	75

^a^ Unless otherwise noted, all reactions were performed with 0.2 mmol of **1a** in solvent (2.0 mL) for 1.0 h. ^b^ hydrobromic acid instead of TMSBr was used.

## Supplementary Materials

The following are available online at https://www.mdpi.com/1420-3049/24/21/3999/s1.

## References

[1] Joule J.A., Mills K. (2000). Heterocylic Chemistry.

[2] Balasubraimanan M., Keay J.G., Katritzky A.R., Rees C.W., Scriven E.F.V. (1996). Comprehensive Heterocyclic Chemistry II.

[3] Michael J.P. (2008). Quinoline, quinazoline and acridone alkaloids. Nat. Prod. Rep..

[4] Boa A.N., Canavan S.P., Hirst P.R., Ramsey C., Stead A.M.W., McConkey G.A. (2005). Synthesis of brequinar analogue inhibitors of malaria parasite dihydroorotate dehydrogenase. Bioorg. Med. Chem..

[5] Diao Y., Lu W., Jin H., Zhu J., Han L., Xu M., Gao R., Shen X., Zhao Z., Liu X. (2012). Discovery of Diverse Human Dihydroorotate Dehydrogenase Inhibitors as Immunosuppressive Agents by Structure-Based Virtual Screening. J. Med. Chem..

[6] Munier-Lehmann H., Lucas-Hourani M., Guillou S., Helynck O., Zanghi G., Noel A., Tangy F., Vidalain P., Janin Y.L. (2015). Original 2-(3-Alkoxy-1H-pyrazol-1-yl)pyrimidine Derivatives as Inhibitors of Human Dihydroorotate Dehydrogenase (DHODH). J. Med. Chem..

[7] Das P., Deng X., Zhang L., Roth M.G., Fontoura B.M.A., Phillips M.A., De Brabander J.K. (2013). SAR-Based Optimization of a 4-Quinoline Carboxylic Acid Analogue with Potent Antiviral Activity. ACS Med. Chem. Lett..

[8] Strekowski L., Say M., Zegrocka O., Tanious F.A., Wilson W.D., Manzel L., Macfarlane D.E. (2003). Bis-4-aminoquinolines: Novel triple-helix DNA intercalators and antagonists of immunostimulatory CpG-oligodeoxynucleotides. Bioorg. Med. Chem..

[9] Gogoi S., Zhao C.-G. (2009). Organocatalyzed enantioselective synthesis of 6-amino-5-cyanodihydropyrano[2,3-*c*]pyrazoles. Tetrahedron Lett..

[10] Tan B., Shi Z., Chua J.P., Zhong G. (2008). Control of Four Stereocenters in an Organocatalytic Domino Double Michael Reaction: Efficient Synthesis of Multisubstituted Cyclopentanes. Org. Lett..

[11] Wang B., Wu F., Wang Y., Liu X., Deng L. (2007). Control of Diastereoselectivity in Tandem Asymmetric Reactions Generating Nonadjacent Stereocenters with Bifunctional Catalysis by Cinchona Alkaloids. J. Am. Chem. Soc..

[12] Biddle M.M., Lin M., Scheidt K.A. (2007). Catalytic Enantioselective Synthesis of Flavanones and Chromanones. J. Am. Chem. Soc..

[13] Enders D., Grondal C., Hüttl M.R.M. (2007). Asymmetric Organocatalytic Domino Reactions. Angew. Chem. Int. Ed..

[14] Bharate J.B., Vishwakarma R.A., Bharate S.B. (2015). Metal-free domino one-pot protocols for quinoline synthesis. RSC Adv..

[15] Prajapati S.M., Patel K.D., Vekariya R.H., Panchal S.N., Patel H.D. (2014). Recent advances in the synthesis of quinolines: A review. RSC Adv..

[16] Hassanin H.M., Ibrahim M.A., Gabr Y.A., Alnamer Y.A. (2012). Synthesis and Chemical Reactivity of Pyrano[3,2-c]quinolinones. J. Heterocycl. Chem..

[17] Wang Y., Peng C., Liu L., Zhao J., Su L., Zhu Q. (2009). Sulfuric acid promoted condensation cyclization of 2-(2-(trimethylsilyl) ethynyl)anilines with arylaldehydes in alcoholic solvents: An efficient one-pot synthesis of 4-alkoxy-2-arylquinolines. Tetrahedron Lett..

[18] Sridharan V., Avendano C., Menendez J.C. (2007). CAN-catalyzed three-component reaction between anilines and alkyl vinyl ethers: Stereoselective synthesis of 2-methyl-1,2,3,4-tetrahydroquinolines and studies on their aromatization. Tetrahedron.

[19] Gharpure S.J., Nanda S.K., Adate P.A., Shelke Y.G. (2017). Lewis Acid Promoted Oxonium Ion Driven Carboamination of Alkynes for the Synthesis of 4-Alkoxy Quinolines. J. Org. Chem..

[20] Mao X.-F., Zhu X.-P., Li D.-Y., Liu P.-N. (2017). Cu-Catalyzed Cascade Annulation of Alkynols with 2-Azidobenzaldehydes: Access to 6H-Isochromeno [4,3-c]quinoline. J. Org. Chem..

[21] Liu Y., Zhang X., Xi C. (2018). MeOTf-induced annulation of arylisocyanates and arylalkynes leading to 4-methoxyl-2,3-diarylquinolines. Tetrahedron Lett..

[22] Yang T., Ding H., Li R., Jin F., Song X.-R., Chen X., Bai J., Xiao Q., Liang Y.-M. (2019). para-TsOH-Promoted Cascade Reaction of ortho-Propynol Phenyl Azides for the Synthesis of 4-Methoxy Quinolines and Propargyl Methyl Ethers: Insight on Mechanism of Propargylic Alcohols. Asian J. Org. Chem..

[23] Abbiati G., Arcadi A., Marinelli F., Rossi E. (2003). Domino [3 + 2] Cycloaddition/Annulation Reactions of β-(2-Aminophenyl)-α,β-ynones with Nitrile Oxides: Synthesis of Isoxazolo[4,5-c]quinolines. Eur. J. Org. Chem..

[24] Strekowski L., Zegrocka O., Windham C., Czarny A. (1997). Practical Synthesis of 4-Chloro-2-(2-naphthyl)quinoline, a Precursor to Triple-Helix DNA Intercalators. Org. Process Res. Dev..

[25] Akila S., Selvi S., Balasubramanian K. (2001). The Vilsmeier cyclization of 2′-azido and 2′-aminochalcones—A mild one pot synthesis of 2-aryl-4-chloroquinoline and its *N*-formyl-1,2-dihydro derivatives. Tetrahedron.

[26] Shvartsberg M.S., Kolodina E.A. (2008). Synthesis of 4-haloquinolines and their fused polycyclic derivatives. Mendeleev Commun..

[27] O’Neill P.M., Bray P.G., Hawley S.R., Ward S.A., Park B.K. (1998). 4-Aminoquinolines—Past, present, and future; A chemical perspective. Pharmacol. Ther..

[28] Blauer G., Akkawi M., Fleischhacker W., Hiessboeck R. (1998). Synthesis and optical properties of the chloroquine enantiomers and their complexes with ferriprotoporphyrin IX in aqueous solution. Chirality.

[29] Egan T.J., Hunter R., Kaschula C.H., Marques H.M., Misplon A., Walden J. (2000). Structure−Function Relationships in Aminoquinolines: Effect of Amino and Chloro Groups on Quinoline−Hematin Complex Formation, Inhibition of β-Hematin Formation, and Antiplasmodial Activity. J. Med. Chem..

[30] Muzart J. (2008). Gold-catalysed reactions of alcohols: Isomerisation, inter- and intramolecular reactions leading to C–C and C–heteroatom bonds. Tetrahedron.

[31] Kabalka G.W., Yao M.-L. (2008). Direct Propargylic Substitution of Hydroxyl Group in Propargylic Alcohols. Curr. Org. Synth..

[32] Zhang L., Fang G., Kumar R.K., Bi X. (2015). Coinage-Metal-Catalyzed Reactions of Propargylic Alcohols. Synthesis.

[33] Zhu Y., Sun L., Lu P., Wang Y. (2014). Recent Advances on the Lewis Acid-Catalyzed Cascade Rearrangements of Propargylic Alcohols and Their Derivatives. ACS Catal..

[34] Song X.-R., Qiu Y.-F., Liu X.-Y., Liang Y.-M. (2016). Recent advances in the tandem reaction of azides with alkynes or alkynols. Org. Biomol. Chem..

[35] Han Y.-P., Li X.-S., Zhu X.-Y., Li M., Zhou L., Song X.-R., Liang Y.-M. (2017). Lewis Acid Catalyzed Dehydrogenative Coupling of Tertiary Propargylic Alcohols with Quinoline N-Oxides. J. Org. Chem..

[36] Han Y.-P., Song X.-R., Qiu Y.-F., Li X.-S., Zhang H.-R., Zhu X.-Y., Liu X.-Y., Liang Y.-M. (2016). Lewis Acid Catalyzed Cyclization of Propargylic Alcohols with 2-Vinylphenol. Org. Lett..

[37] Han Y.-P., Song X.-R., Qiu Y.-F., Zhang H.-R., Li L.-H., Jin D.-P., Sun X.-Q., Liu X.-Y., Liang Y.-M. (2016). Lewis Acid Catalyzed [4 + 3] Cycloaddition of Propargylic Alcohols with Azides. Org. Lett..

[38] Qiu Y.-F., Song X.-R., Li M., Zhu X.-Y., Wang A.-Q., Yang F., Han Y.-P., Zhang H.-R., Jin D.-P., Li Y.-X. (2016). BF_3_·OEt_2_-AgSCF_3_ Mediated Trifluoromethylthiolation/Cascade Cyclization of Propynols: Synthesis of 4-((Trifluoromethyl)thio)-2*H*-chromene and 4-((Trifluoromethyl)thio)-1,2-dihydroquinoline Derivatives. Org. Lett..

[39] Li R., Jin F., Song X.-R., Yang T., Ding H., Yang R., Xiao Q., Liang Y.-M. (2019). Acid-promoted cyclization of 2-propynolphenols leading to 4-tosyloxy-2*H*-chromenes. Tetrahedron Lett..

[40] Yang T., Song X.-R., Li R., Jin F., Zhang Y., Bai J., Yang R., Ding H., Xiao Q. (2019). Metal-free and efficient approach to 4-thiocyanated 2*H*-chromenes via TFA-mediated cascade cyclization of 2-propynolphenols. Tetrahedron Lett..

[41] Li R., Song X.-R., Chen X., Ding H., Xiao Q., Liang Y.-M. (2017). Copper-Catalyzed Cascade Cyclization of 2-Propynolphenols: Access to 4-Phosphorylated 2*H*-Chromenes. Adv. Synth. Catal..

[42] Song X.-R., Li R., Yang T., Chen X., Ding H., Xiao Q., Liang Y.-M. (2018). Novel and Efficient Access to Flavones under Mild Conditions: Aqueous HI-Mediated Cascade Cyclization/Oxidative Radical Reaction of 2-Propynolphenols. Eur. J. Org. Chem..

[43] Yang T., Kou P., Jin F., Song X.-R., Bai J., Ding H., Xiao Q., Liang Y.-M. (2019). TFA-Promoted Sulfonation/Cascade Cyclization of 2-Propynolphenols with Sodium Sulfinates to 4-Sulfonyl 2*H*-Chromenes under Metal-free Conditions. Org. Chem. Front..

[44] Song X.-R., Li R., Ding H., Yang R., Xiao Q., Liang Y.-M. (2016). Highly efficient access to 4-chloro-2H-chromenes and 1,2-dihydroquinolines under mild conditions: TMSCl-mediated cyclization of 2-propynolphenols/anilines. Tetrahedron Lett..

[45] Song X.-R., Li R., Ding H., Chen X., Yang T., Jiang B., Xiao Q., Liang Y.-M. (2018). An efficient approach to 4-chloro quinolines via TMSCl-mediated cascade cyclization of ortho-propynol phenyl azides. Org. Chem. Front..

[46] Wada T., Iwasaki M., Kondoh A., Yorimitsu H., Hideki Yorimitsu Oshima K. (2010). Palladium-Catalyzed Addition of Silyl-Substituted Chloroalkynes to Terminal Alkynes. Chem. Eur. J..

[47] Gopinath V.S., Pinjari J., Dere R.T., Verma A., Vishwakarma P., Shivahare R., Moger M., Goud P.S.K., Ramanathan V., Bose P. (2013). Design, synthesis and biological evaluation of 2-substituted quinolines as potential antileishmanial agents. Eur. J. Med. Chem..

[48] Gopinath V.S., Rao M., Shivahare R., Vishwakarma P., Ghose S., Pradhan A., Hindupur R., Sarma K.D., Gupta S., Puri S.K. (2014). Design, synthesis, ADME characterization and antileishmanial evaluation of novel substituted quinoline analogs. Bioorg. Med. Chem. Lett..

[49] Michael J.P. (2001). Quinoline, quinazoline and acridone alkaloids. Nat. Prod. Rep..

[50] Michael J.P. (2003). Quinoline, quinazoline and acridone alkaloids. Nat. Prod. Rep..

[51] Musiol R., Serda M., Bielowka S.H., Polanski J. (2010). Quinoline-Based Antifungals. Curr. Med. Chem..

[52] Solomon V.R., Lee H. (2011). Quinoline as a Privileged Scaffold in Cancer Drug Discovery. Curr. Med. Chem..

[53] Zhang H., Tanimoto H., Morimoto T., Nishiyama Y., Kakiuchi K. (2013). Regioselective Rapid Synthesis of Fully Substituted 1,2,3-Triazoles Mediated by Propargyl Cations. Org. Lett..

[54] Zhu Y., Yin G., Hong D., Lu P., Wang Y. (2011). Tandem Reaction of Propargylic Alcohol, Sulfonamide, and N-Iodosuccinimide: Synthesis of N-(2-Iodoinden-1-yl)arenesulfonamide. Org. Lett..

[55] Adeloye A.O., Mphahlele M.J. (2011). 2,4-Diarylquinolines: Synthesis, absorption and emission properties. J. Chem. Res..

[56] Abbitati G., Arcadi A., Marinelli F., Rossi E., Verdecchia M. (2006). Rh-Catalyzed Sequential Hydroarylation/Hydrovinylation–Heterocyclization of b-(2-Aminophenyl)-a,b-ynones with Organoboron Derivatives: A New Approach to Functionalized Quinolines. Synlett.

[57] Azizi K., Akrami S., Madsen R. (2019). Manganese(III) Porphyrin-Catalyzed Dehydrogenation of Alcohols to form Imines, Tertiary Amines and Quinolines. Chem. Eur. J..

