# Supplementary figures and images for: TMSBr-Promoted Cascade Cyclization of ortho-Propynol Phenyl Azides for the Synthesis of 4-Bromo Quinolines and Its Applications
Source: Molecules. 2019 Nov 5;24(21):3999. doi: 10.3390/molecules24213999 (PMC6864654; doi:10.3390/molecules24213999)

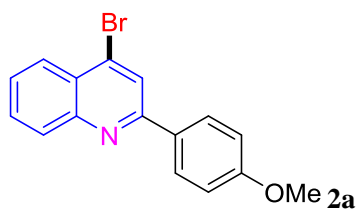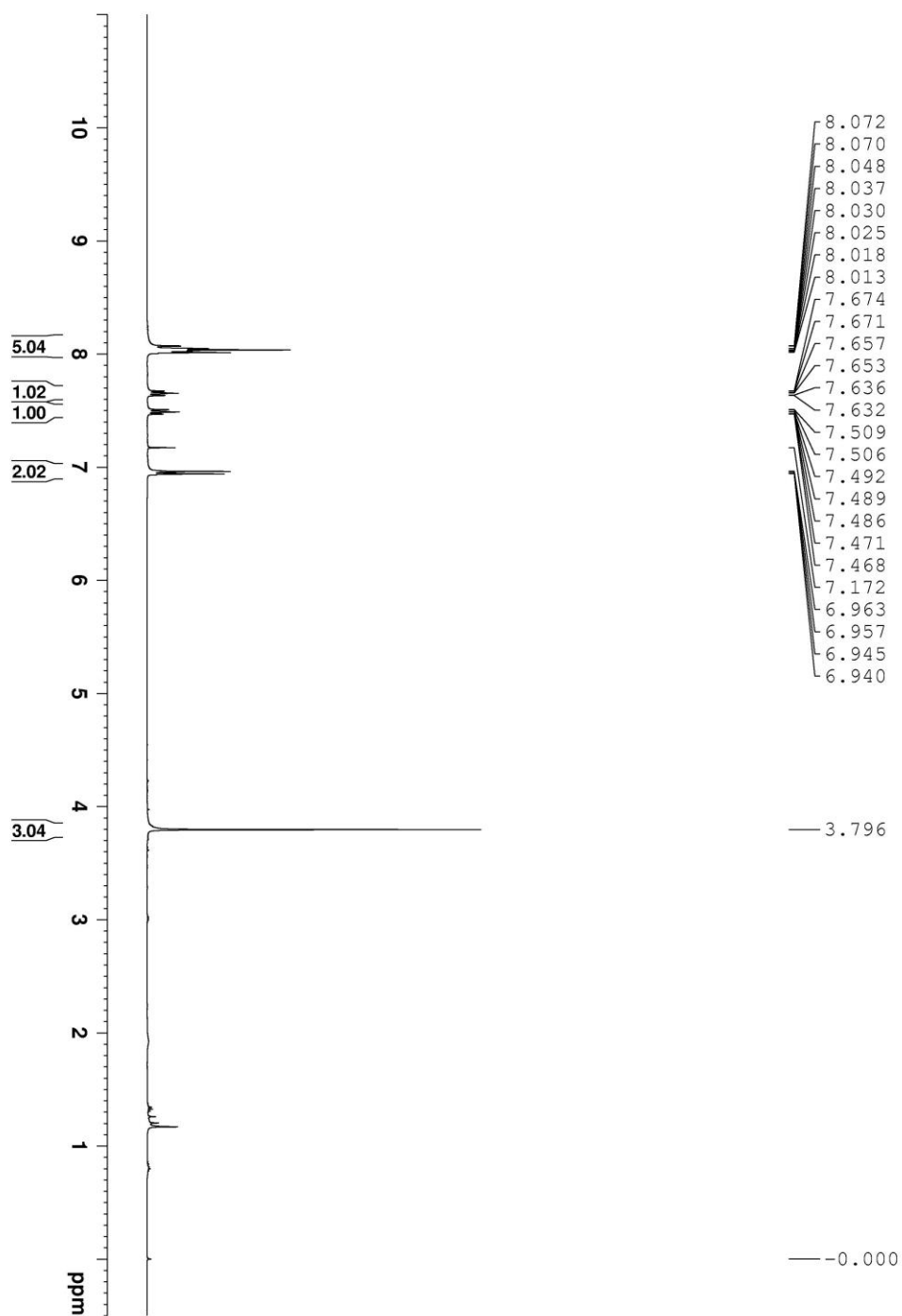

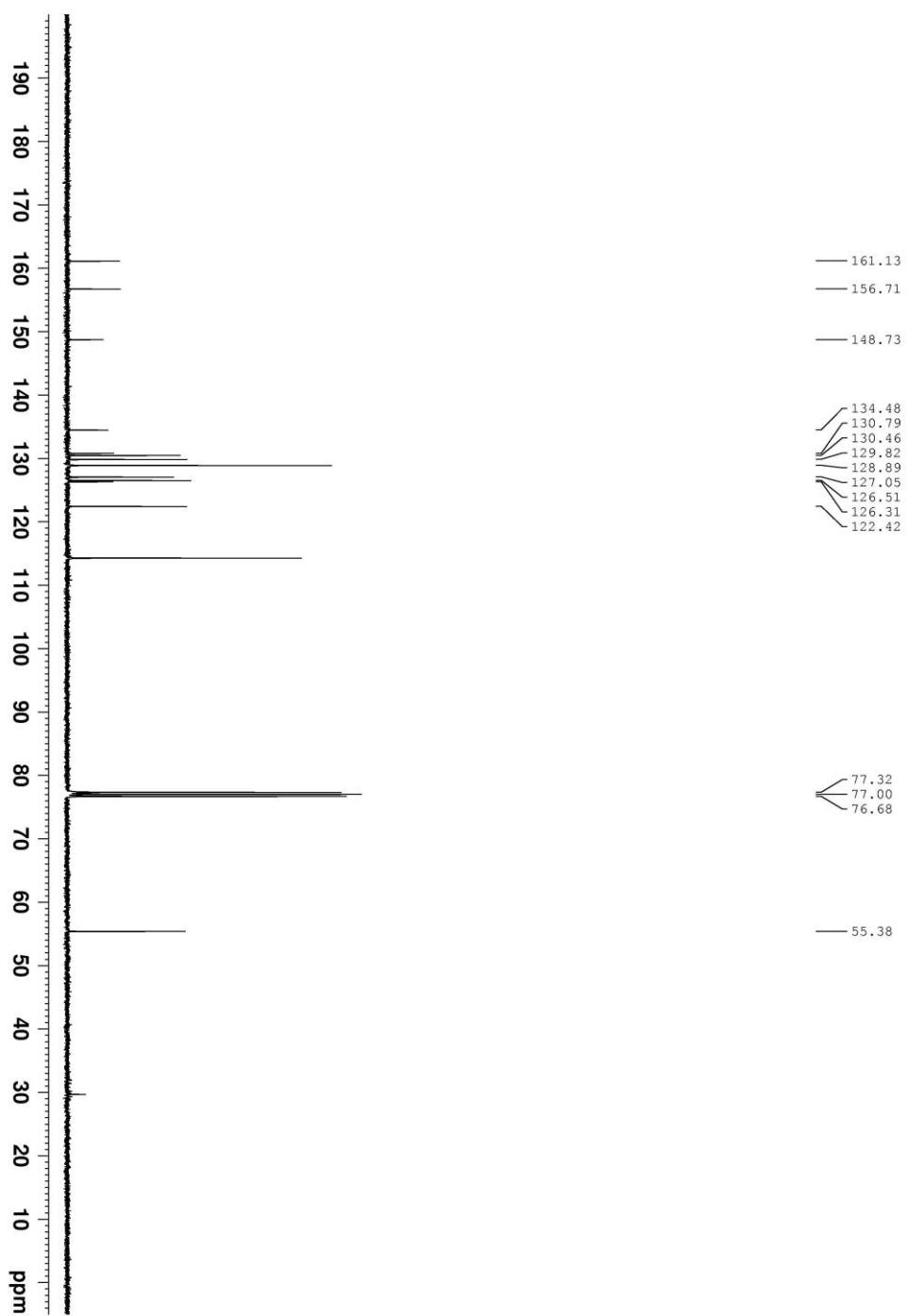

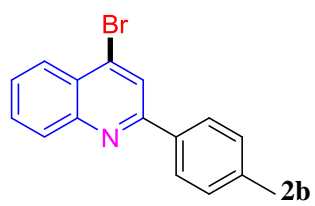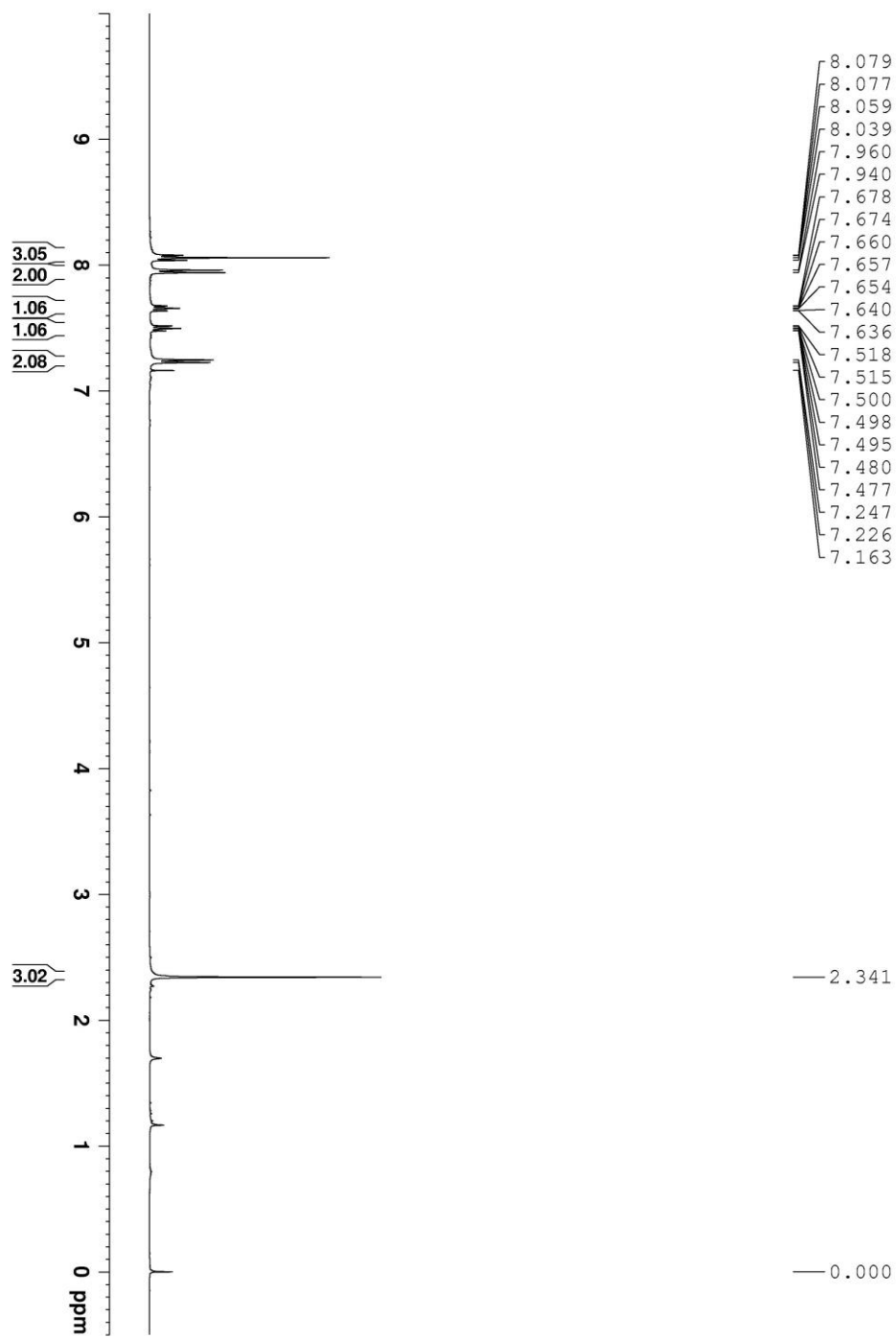

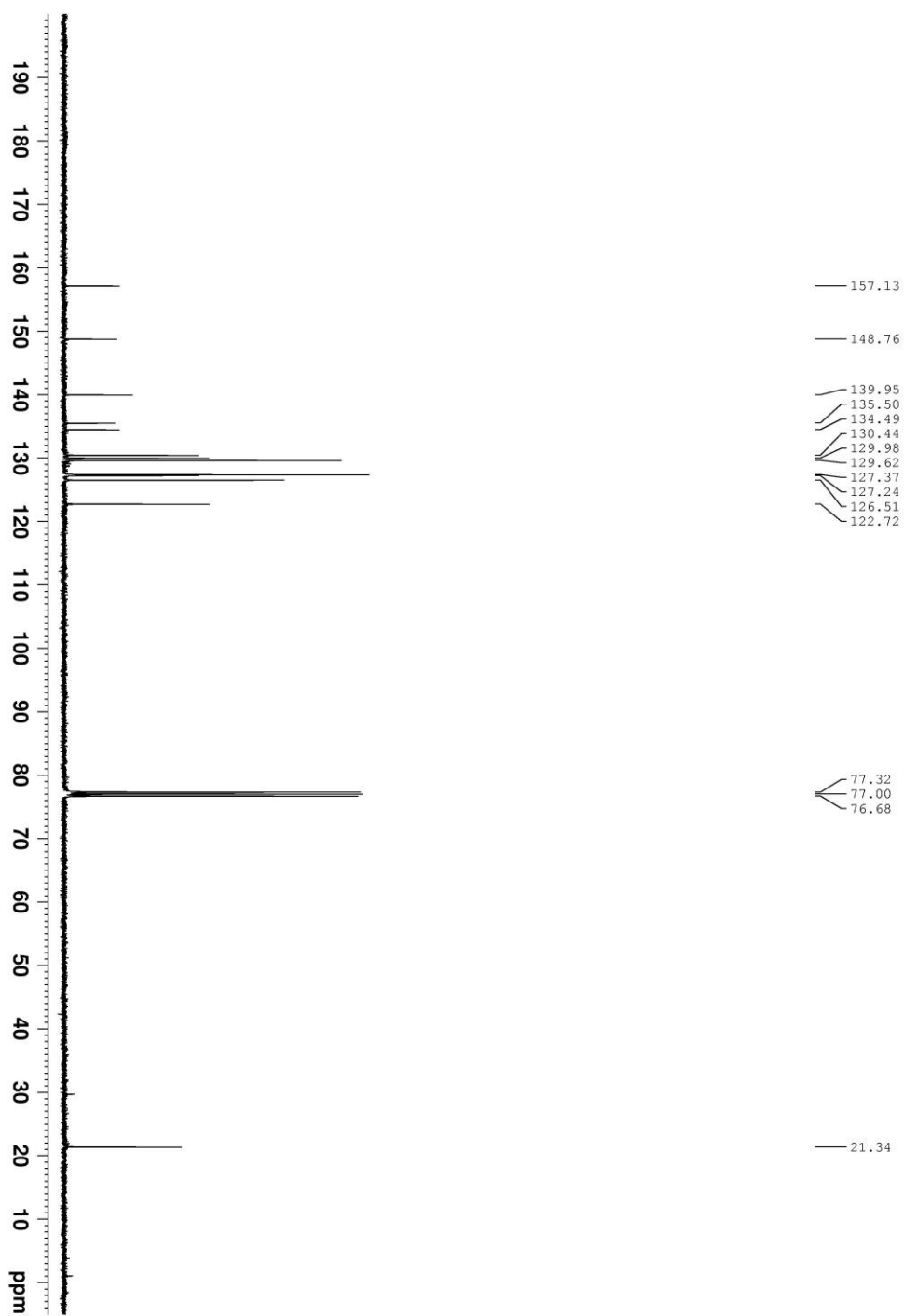

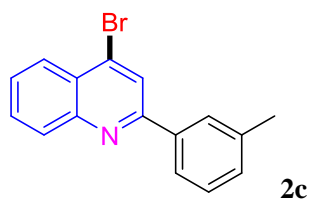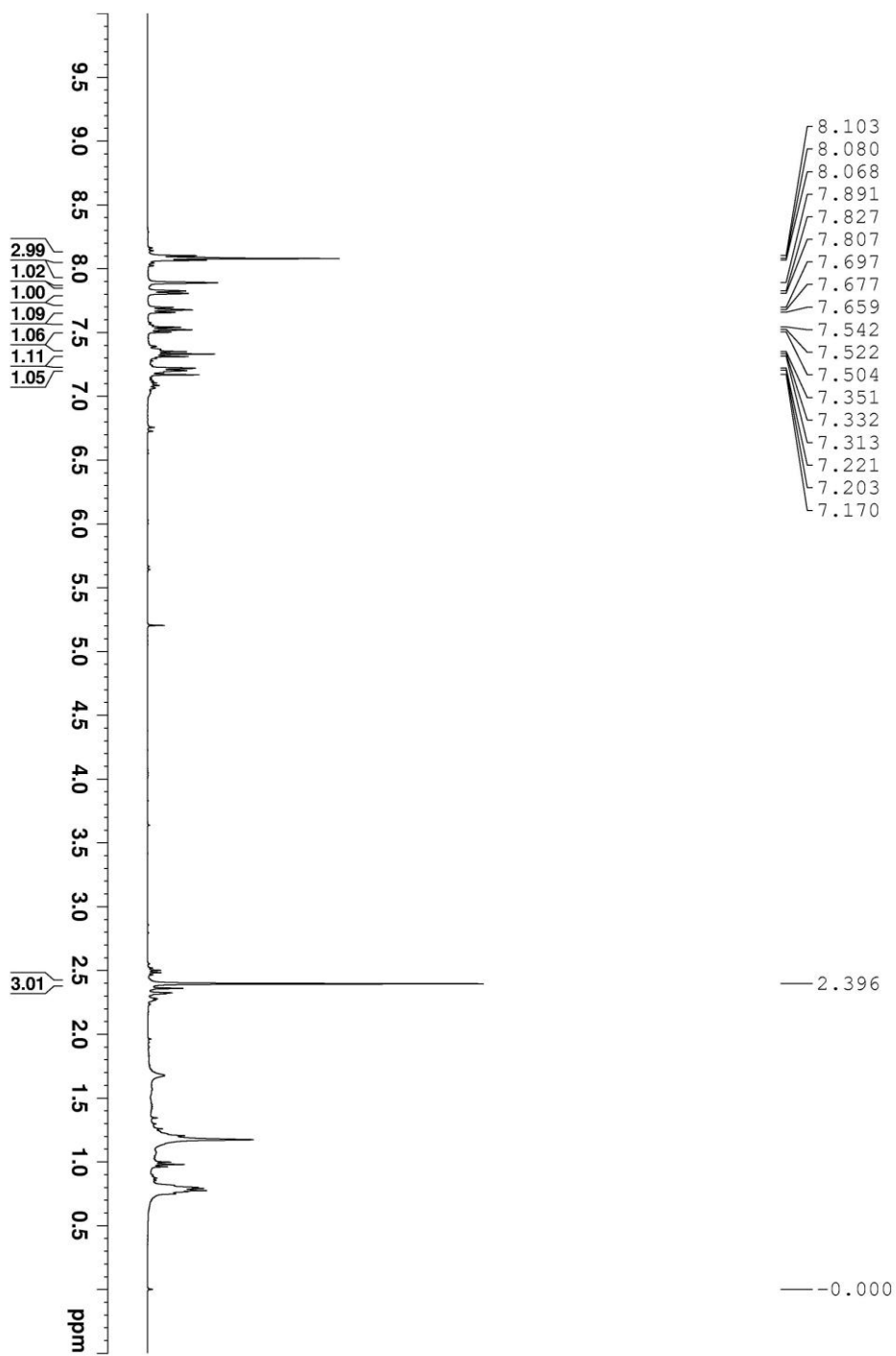

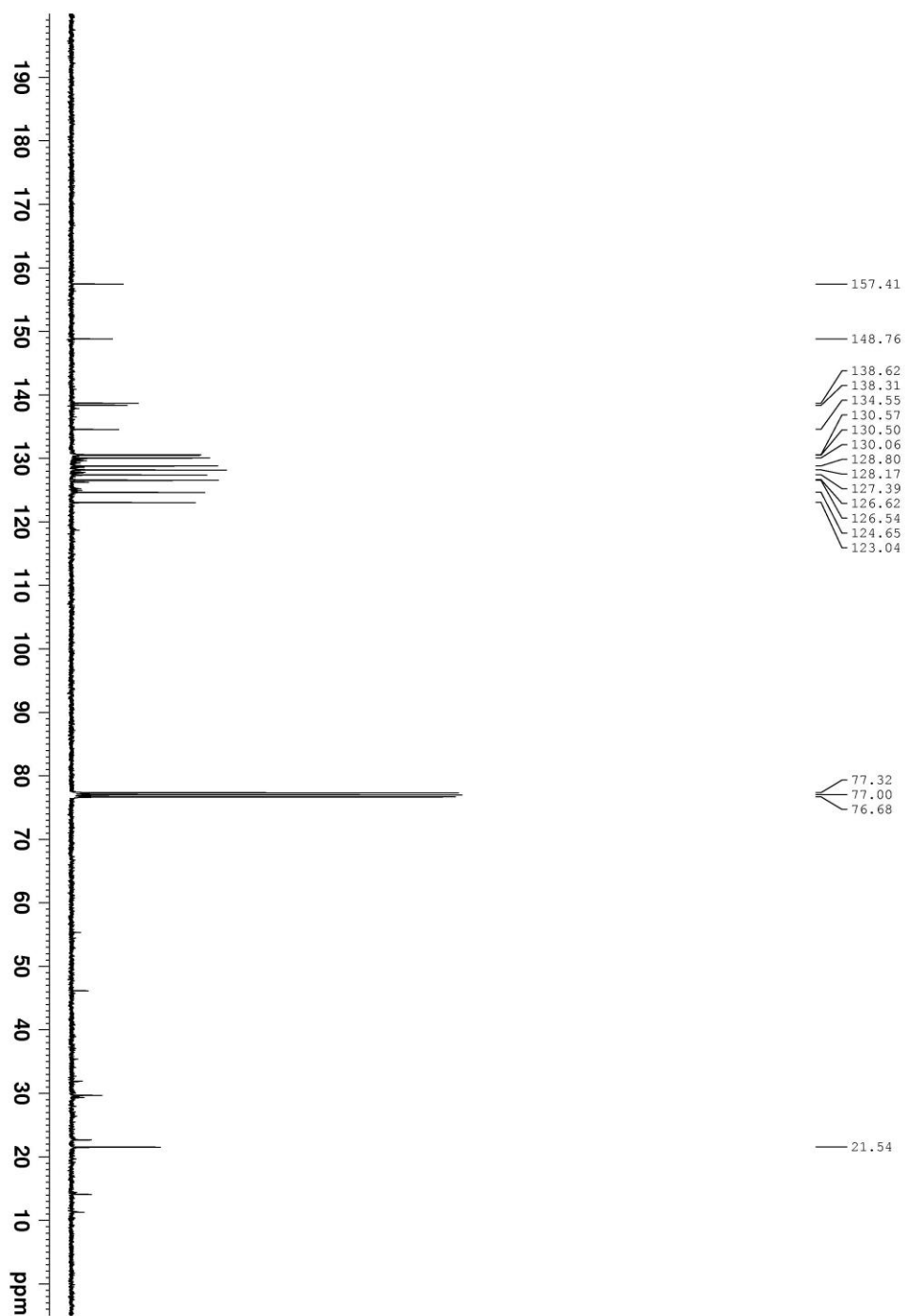

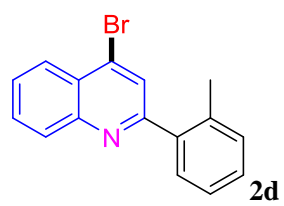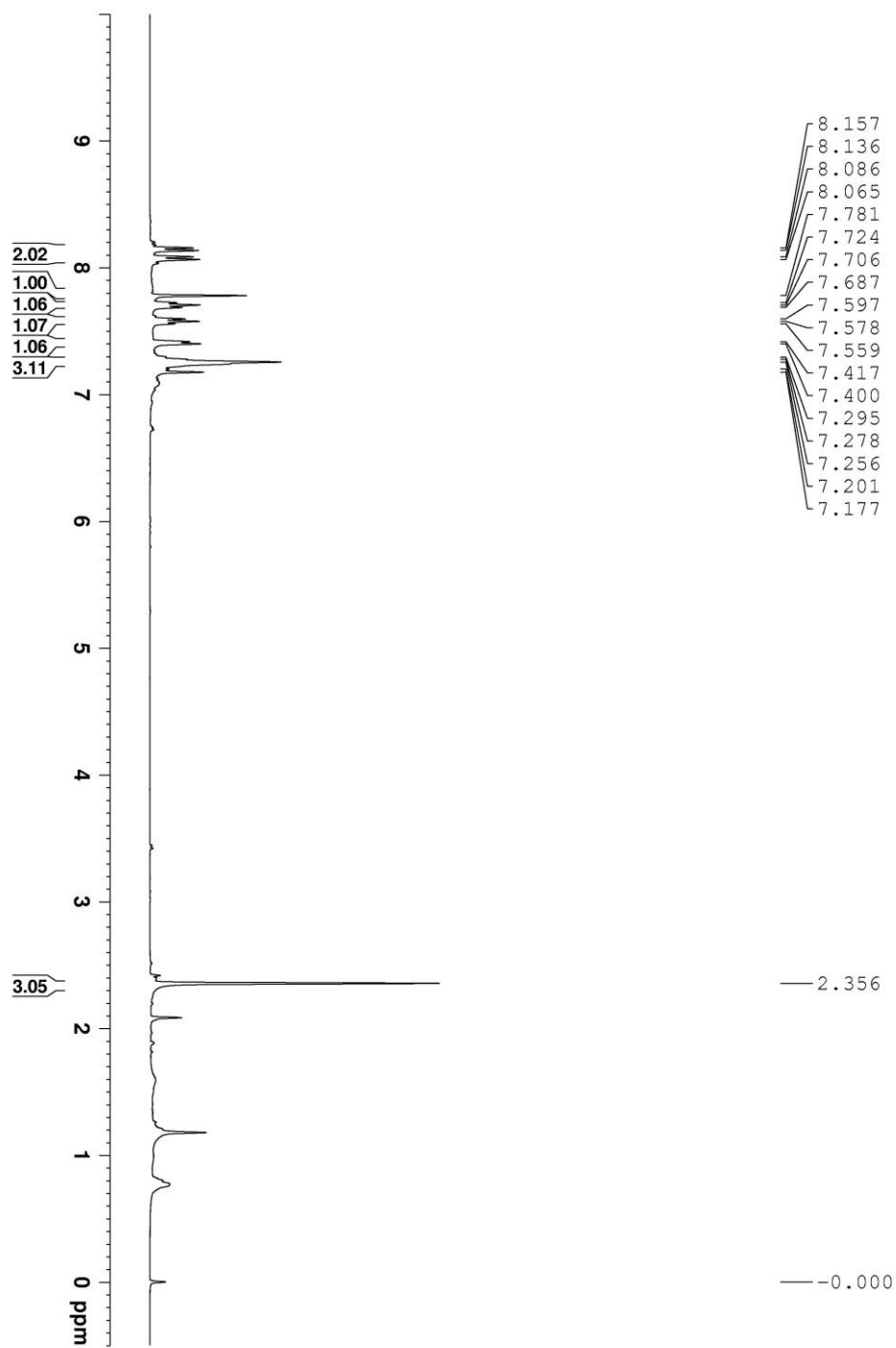

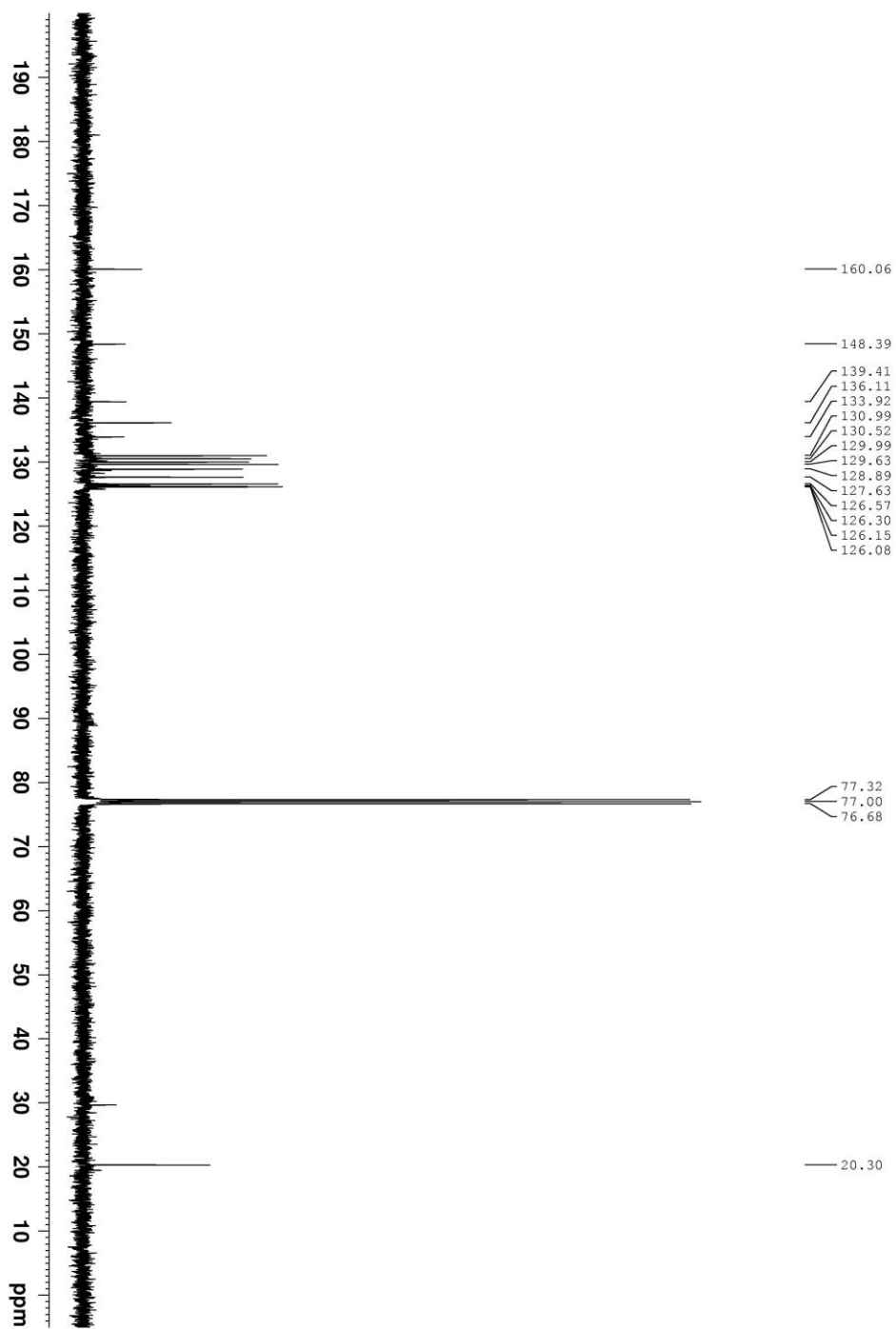

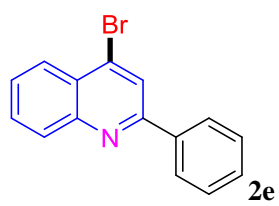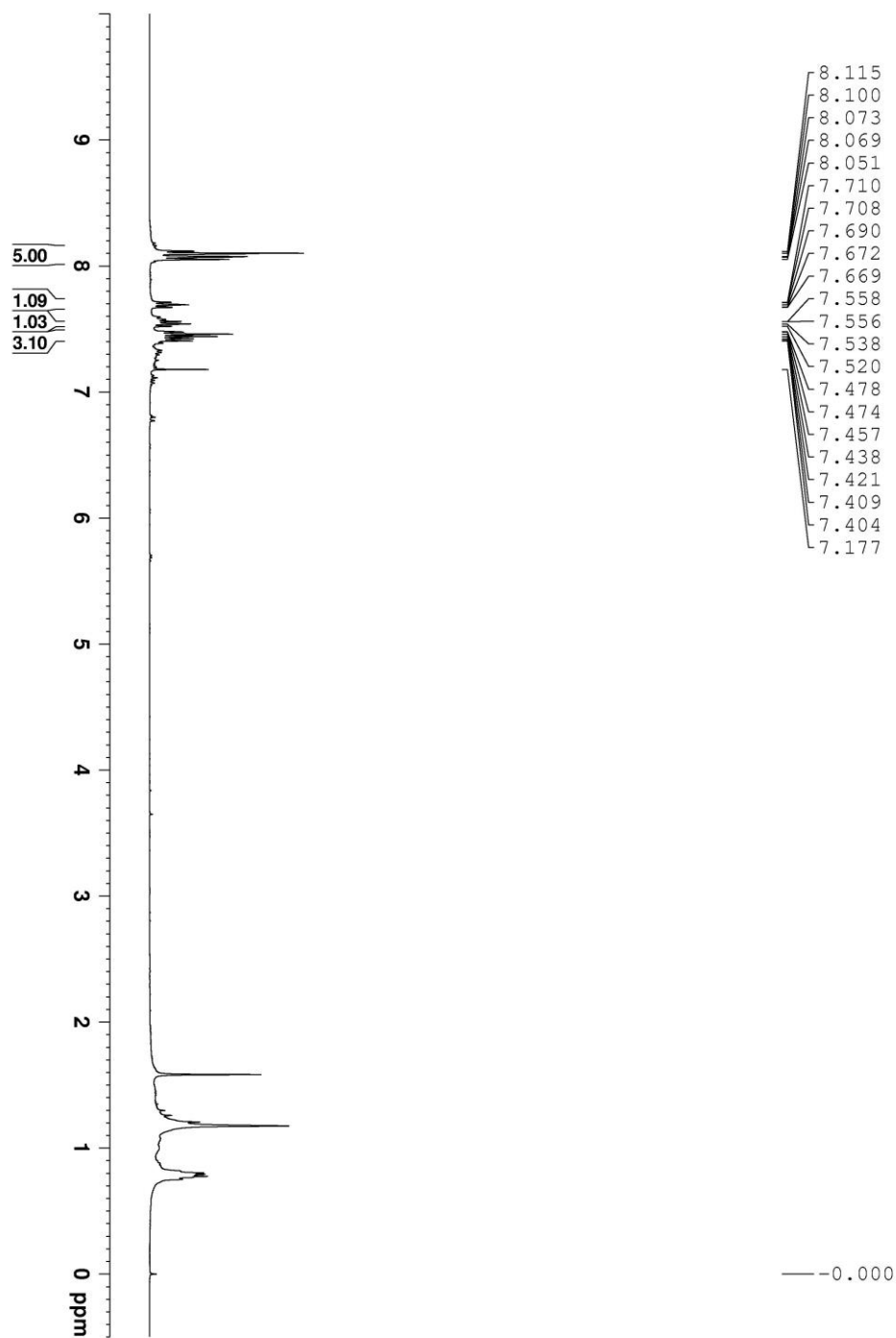

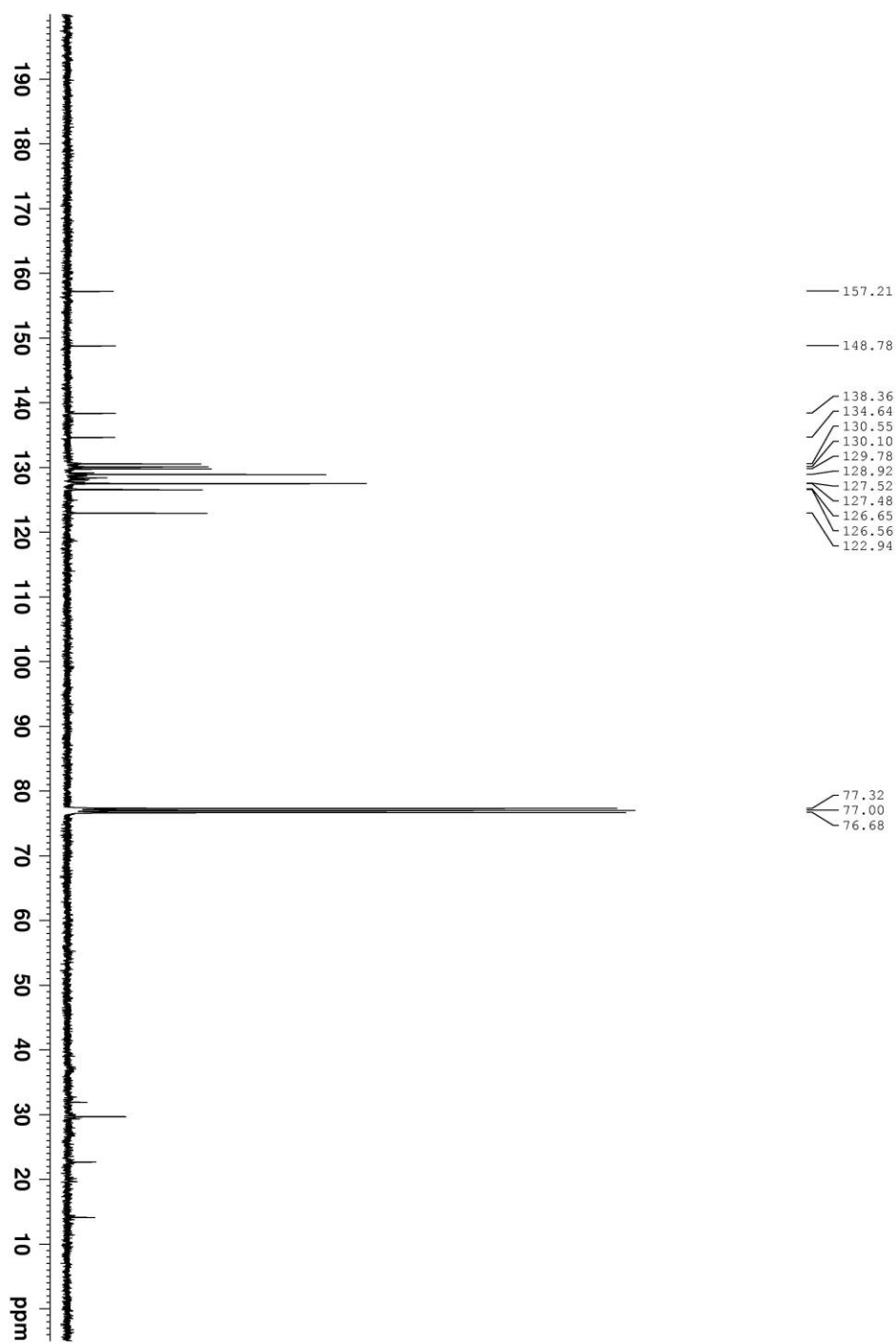

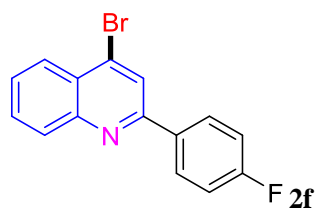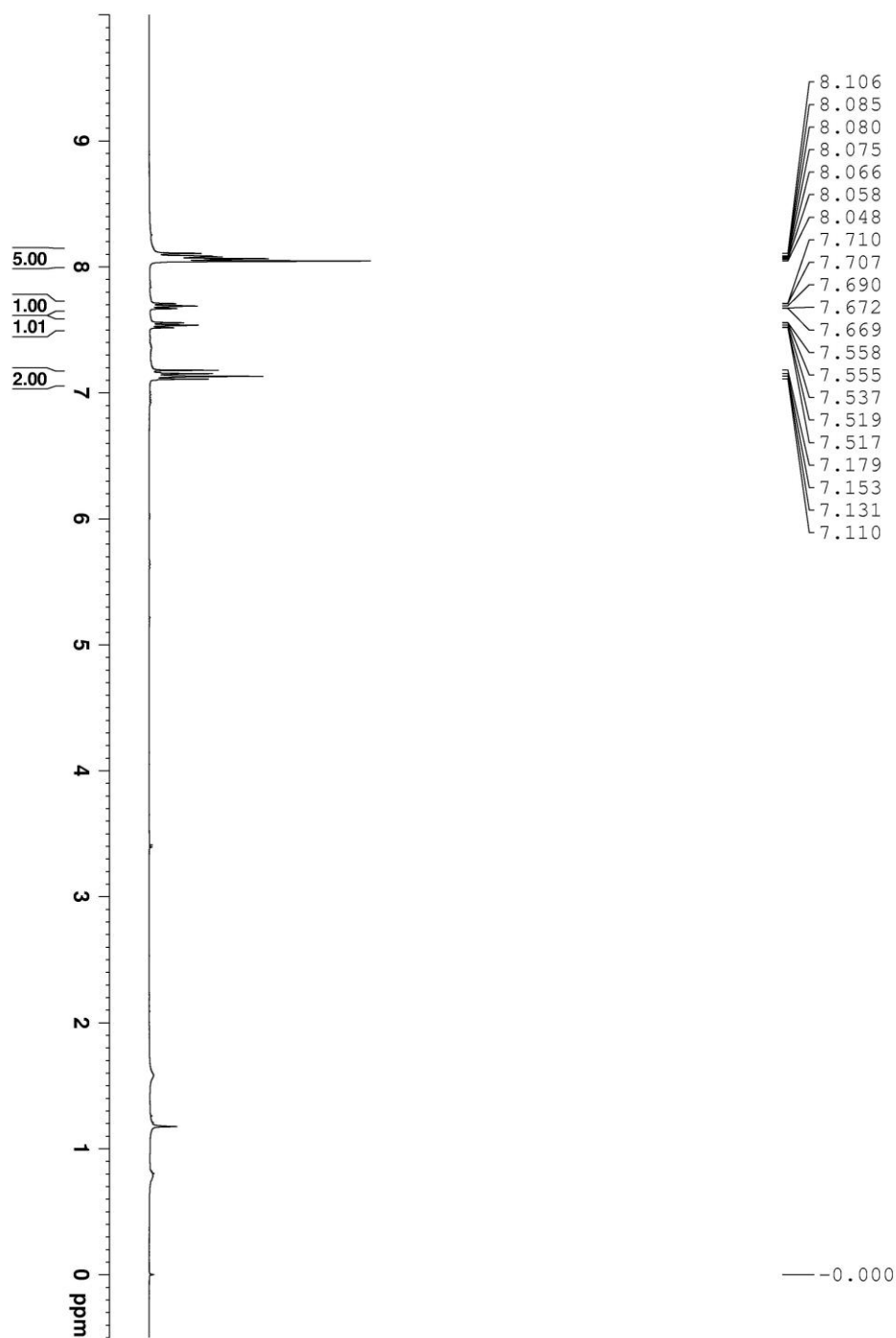

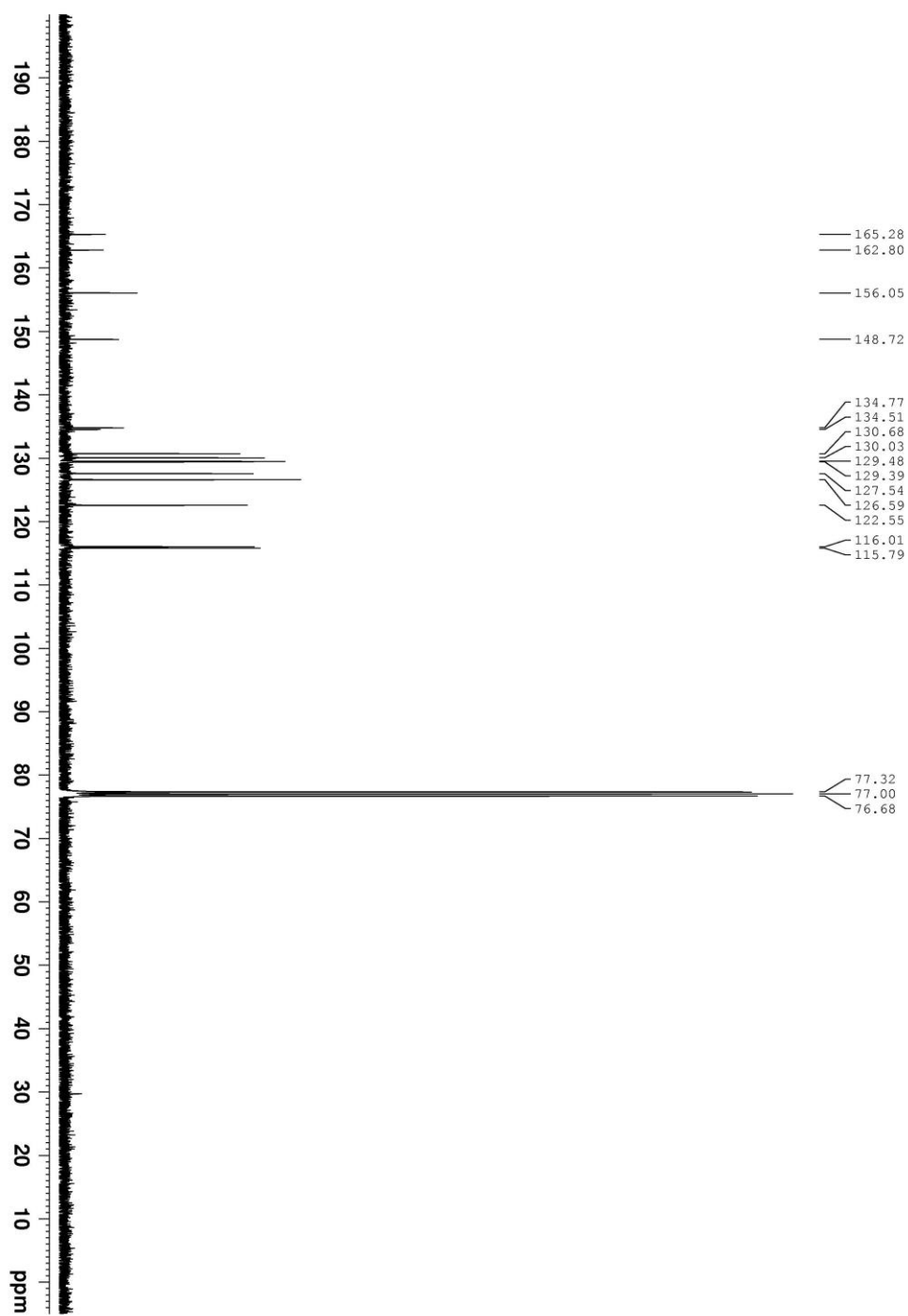

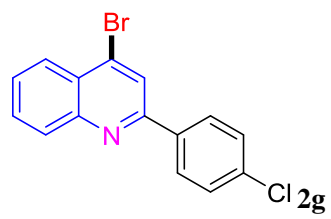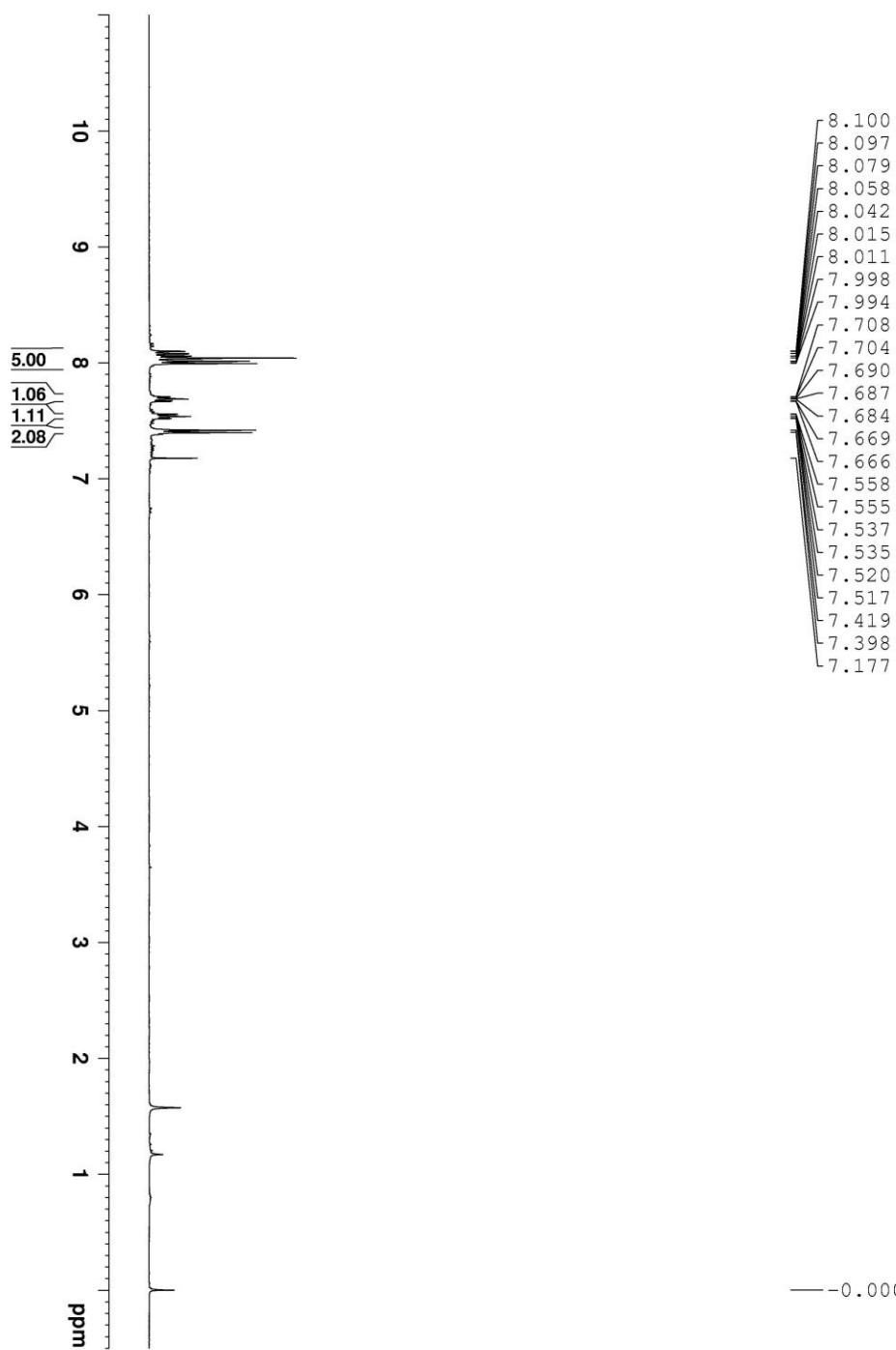

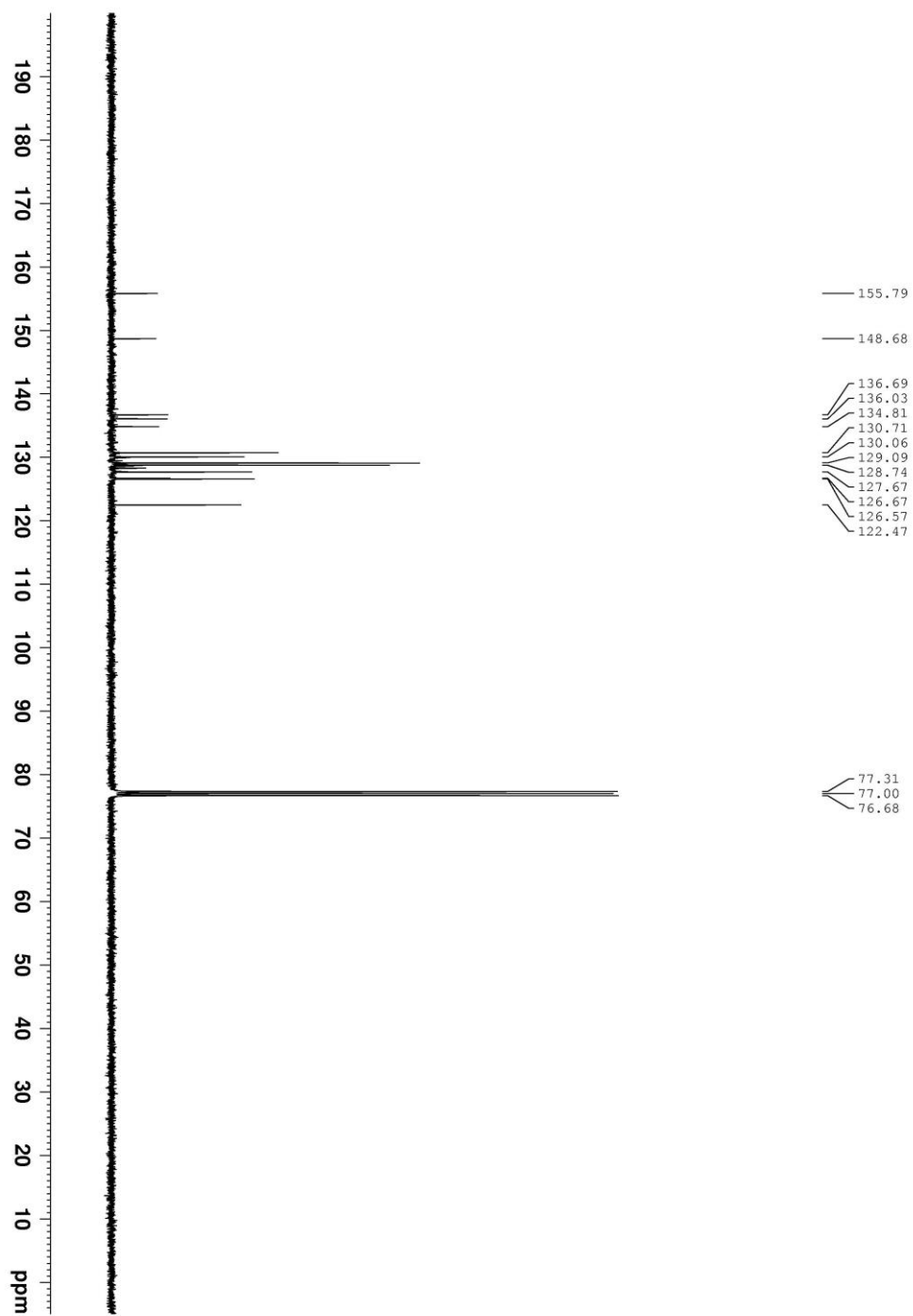

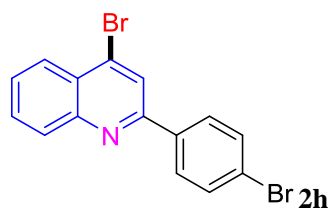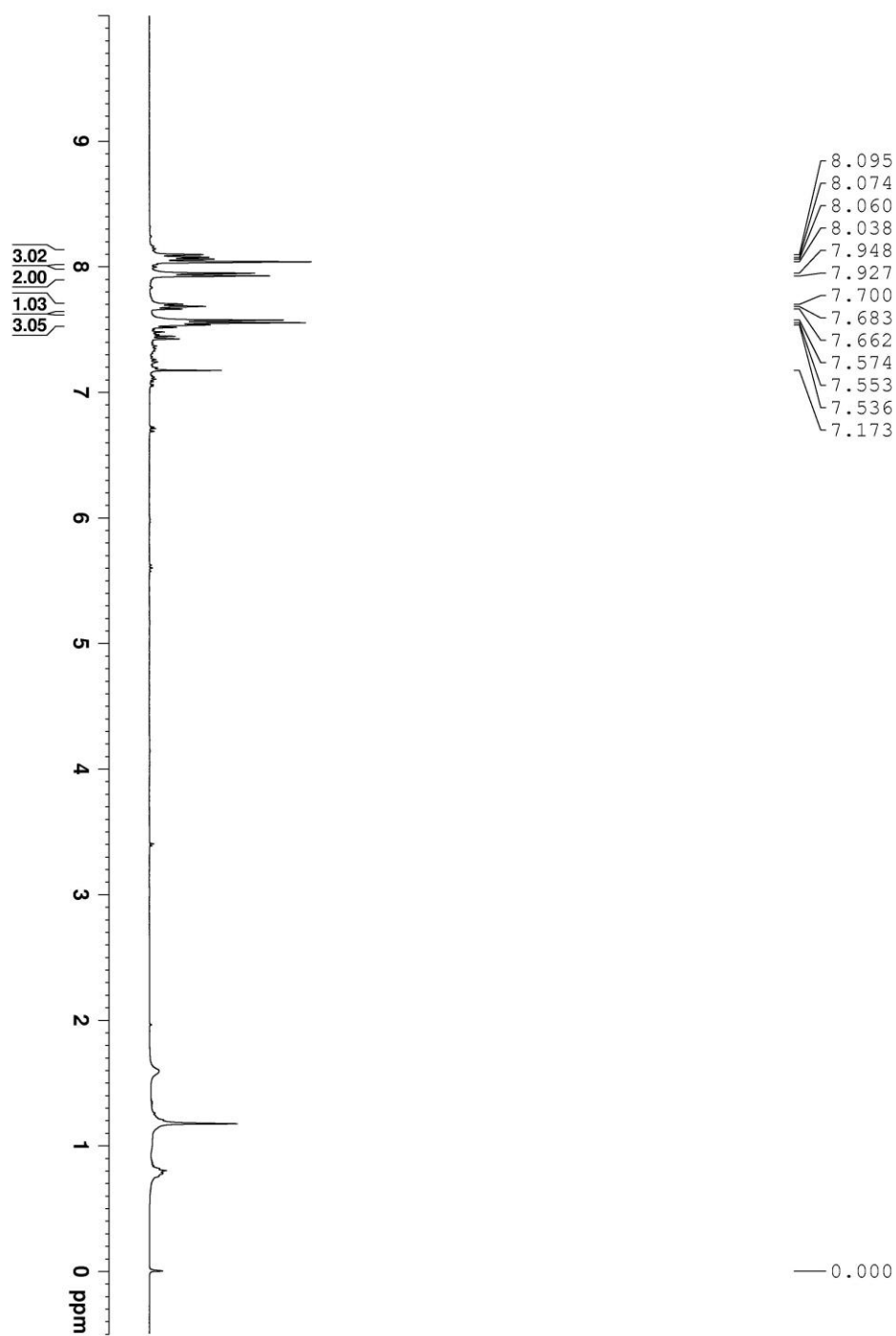

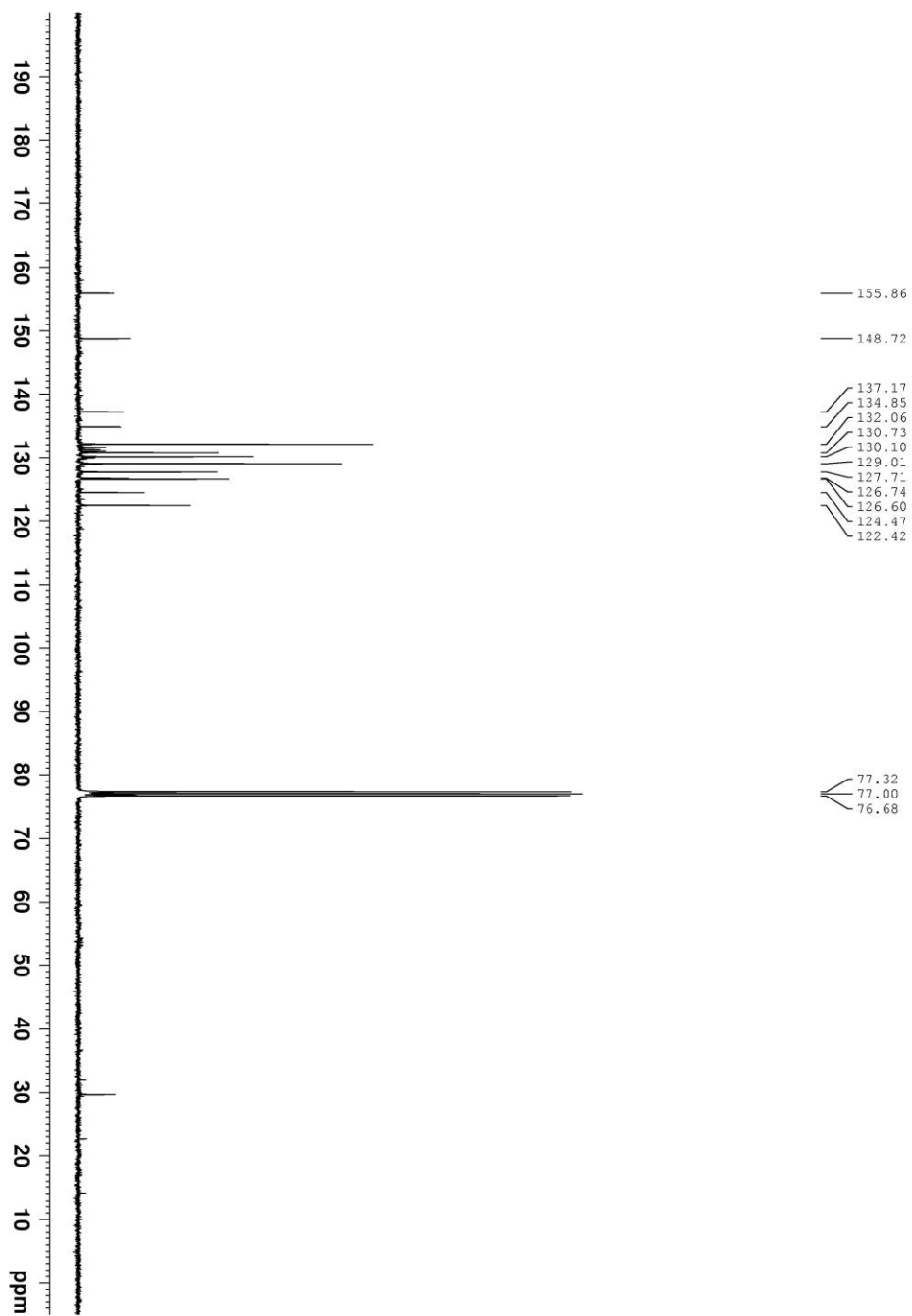

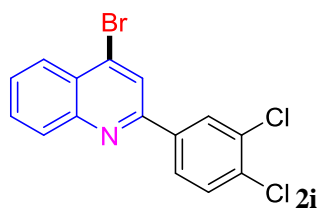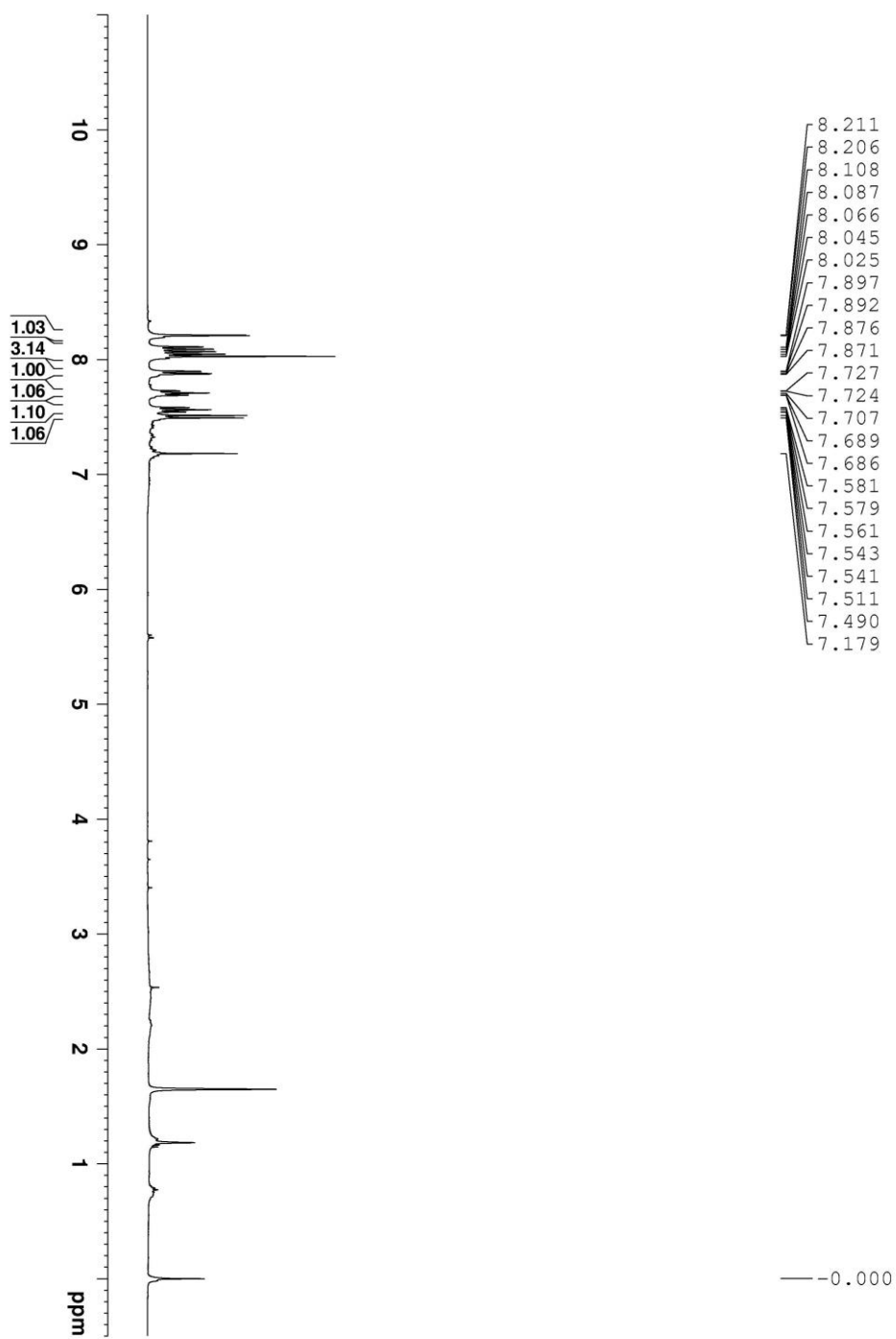

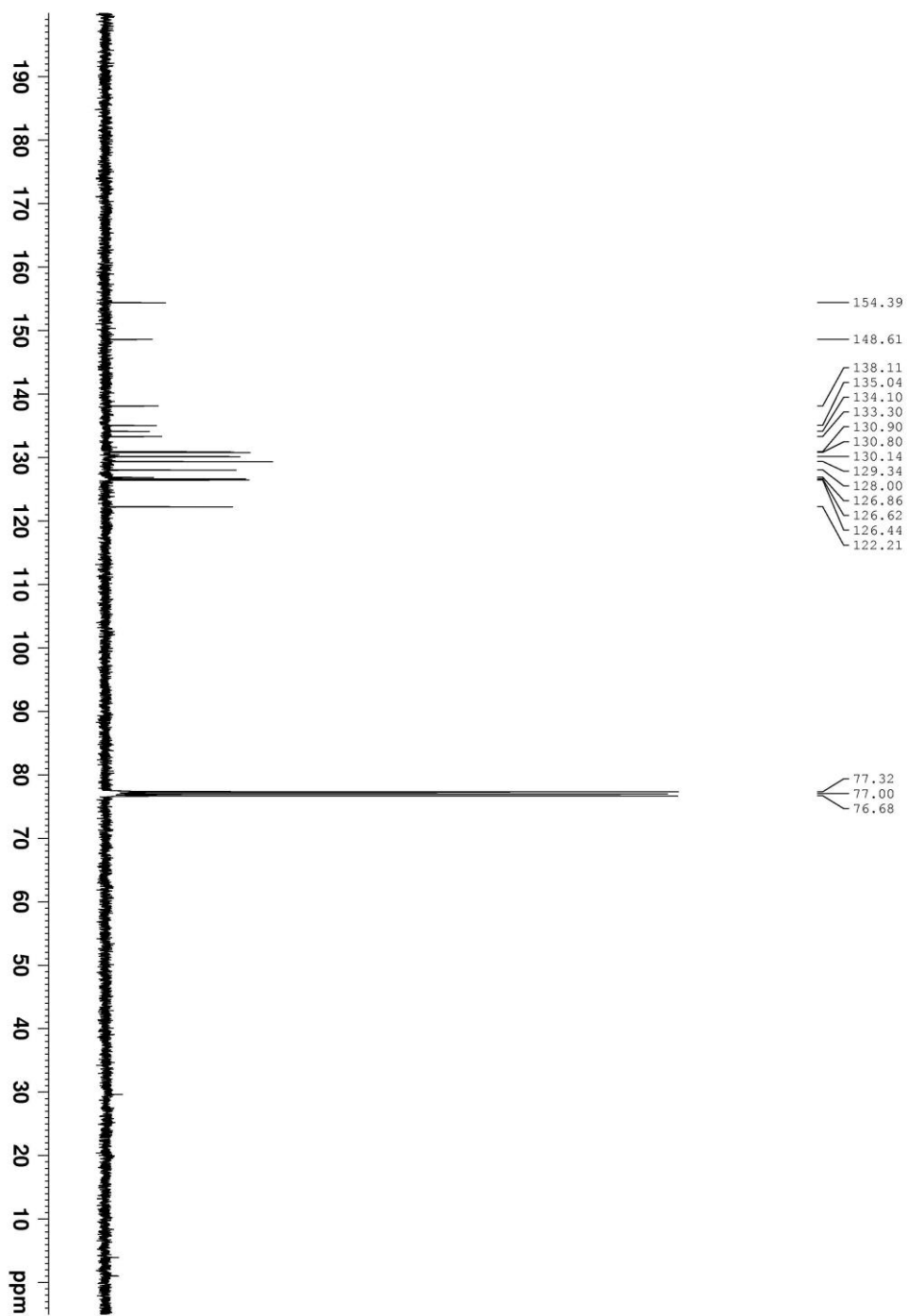

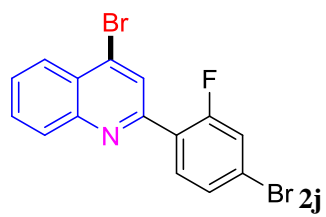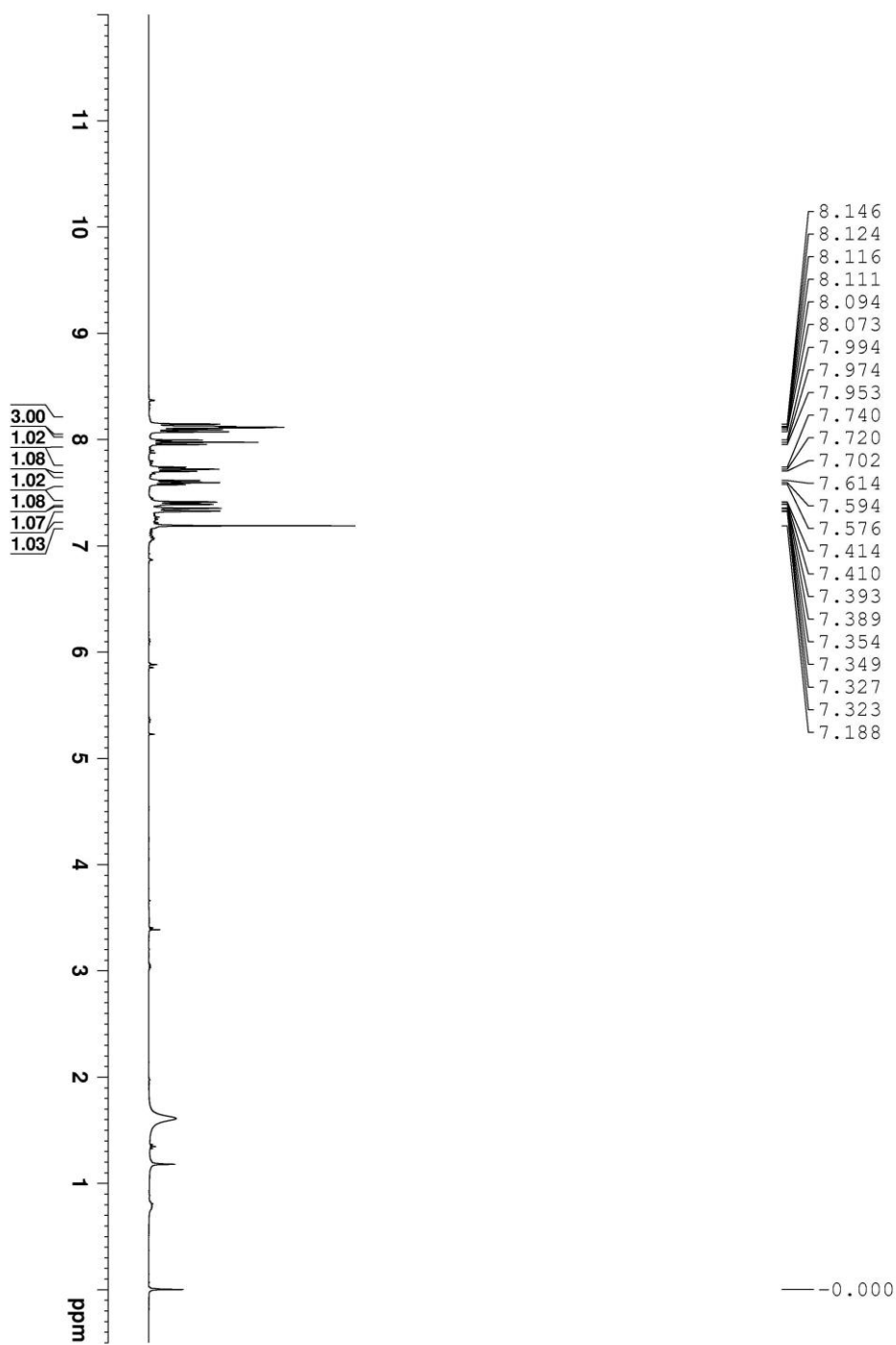

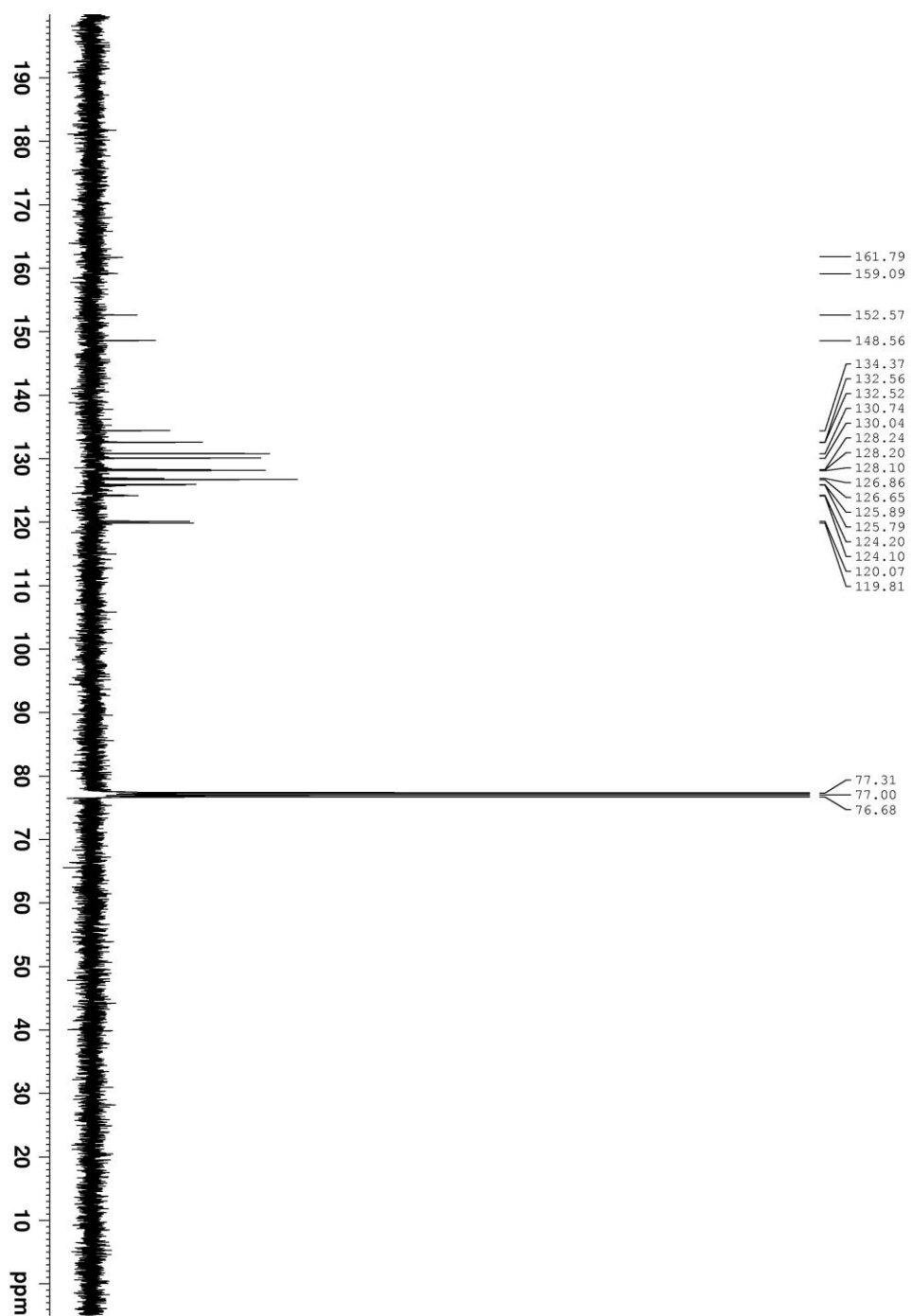

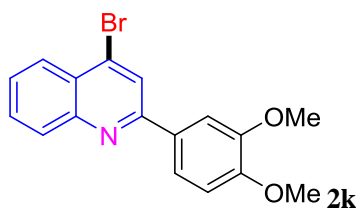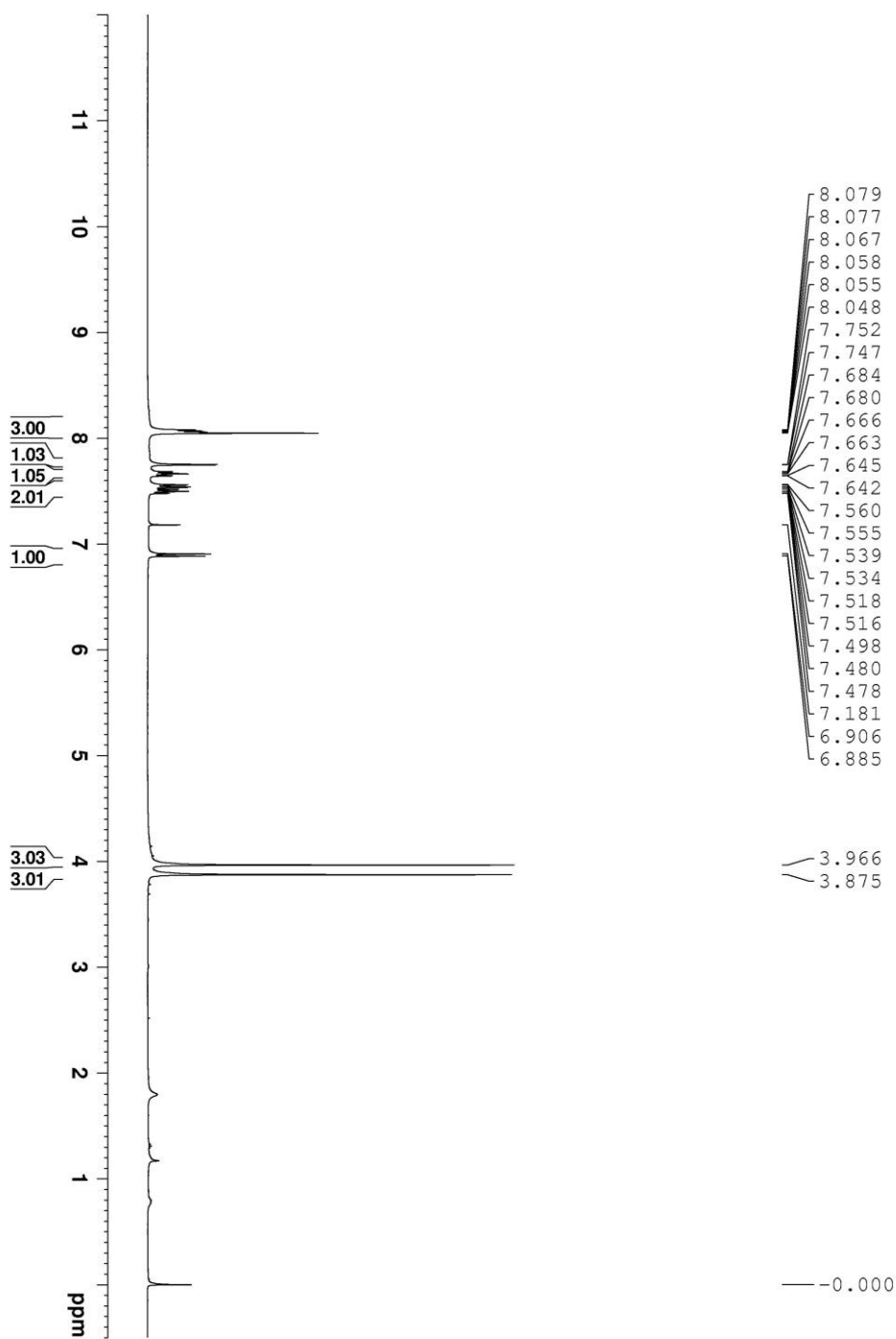

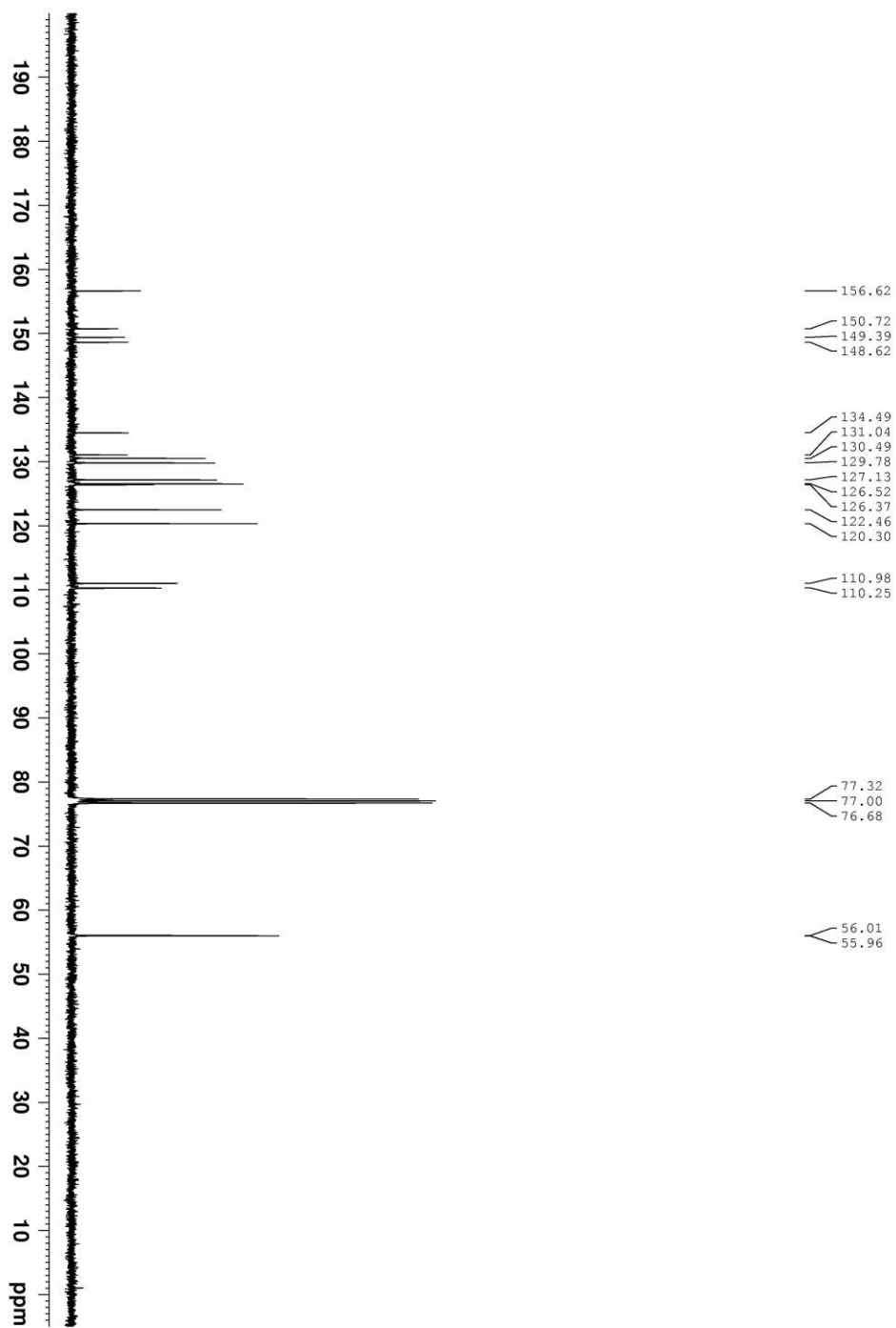

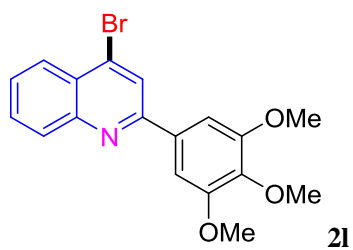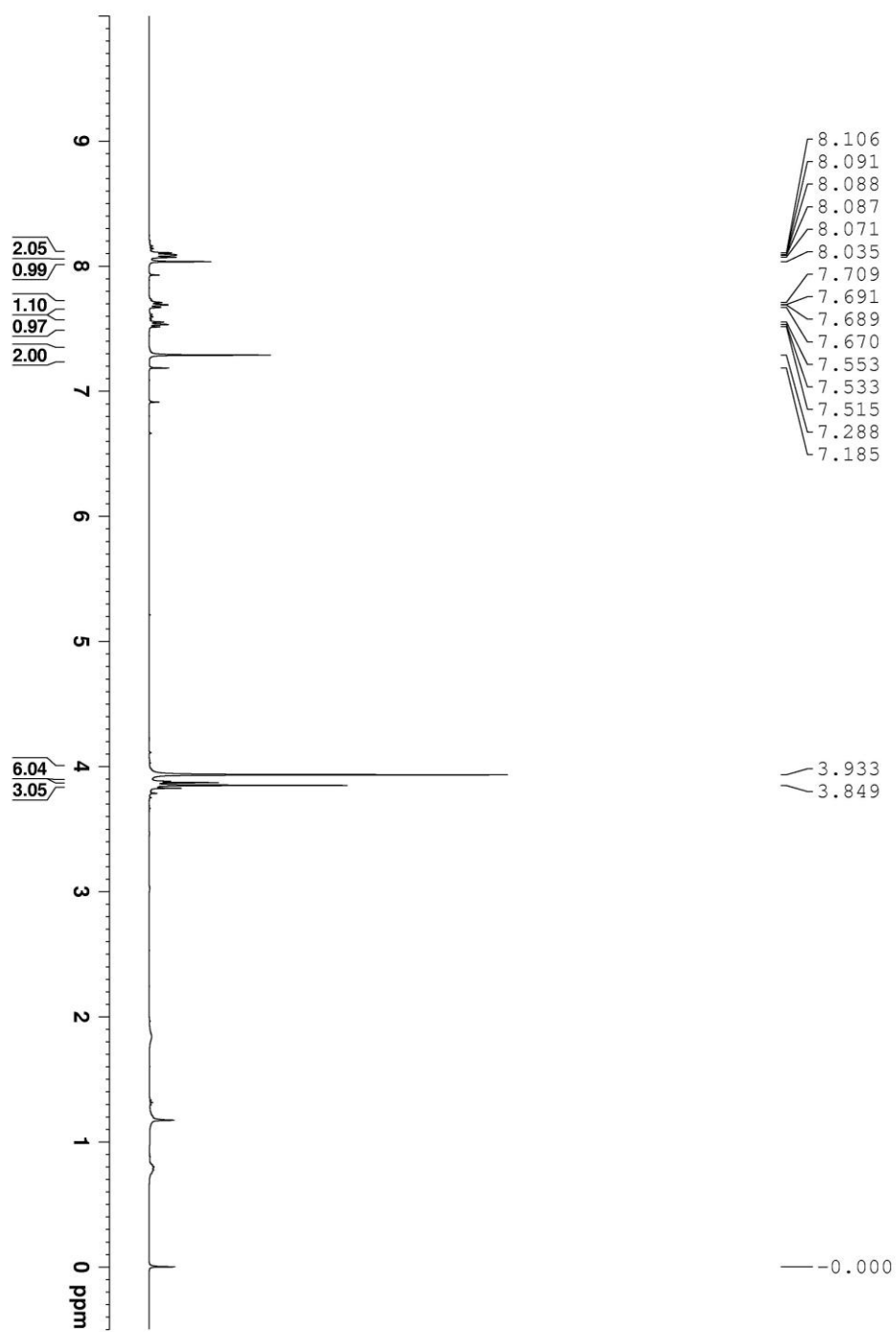

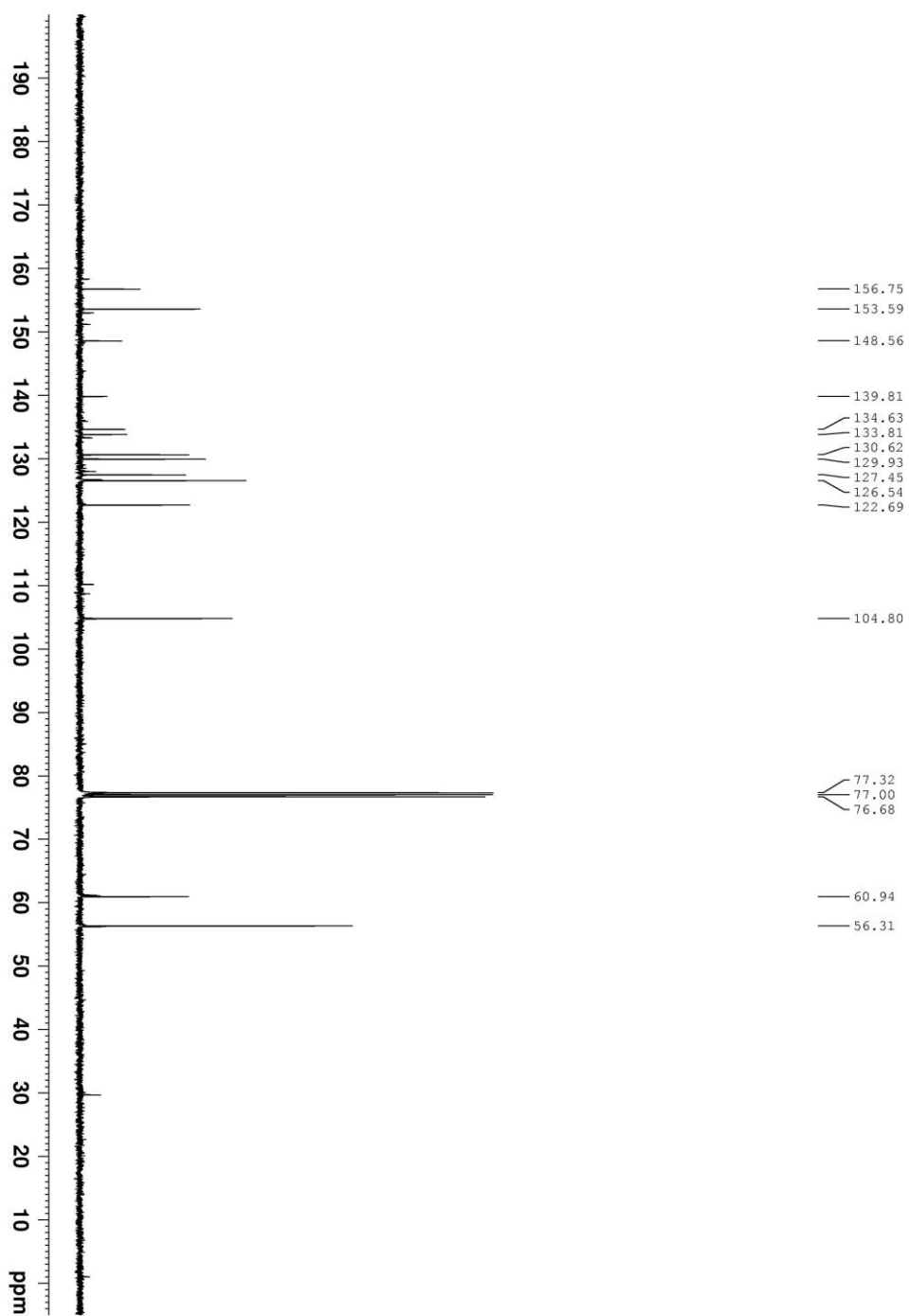

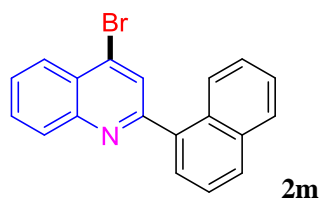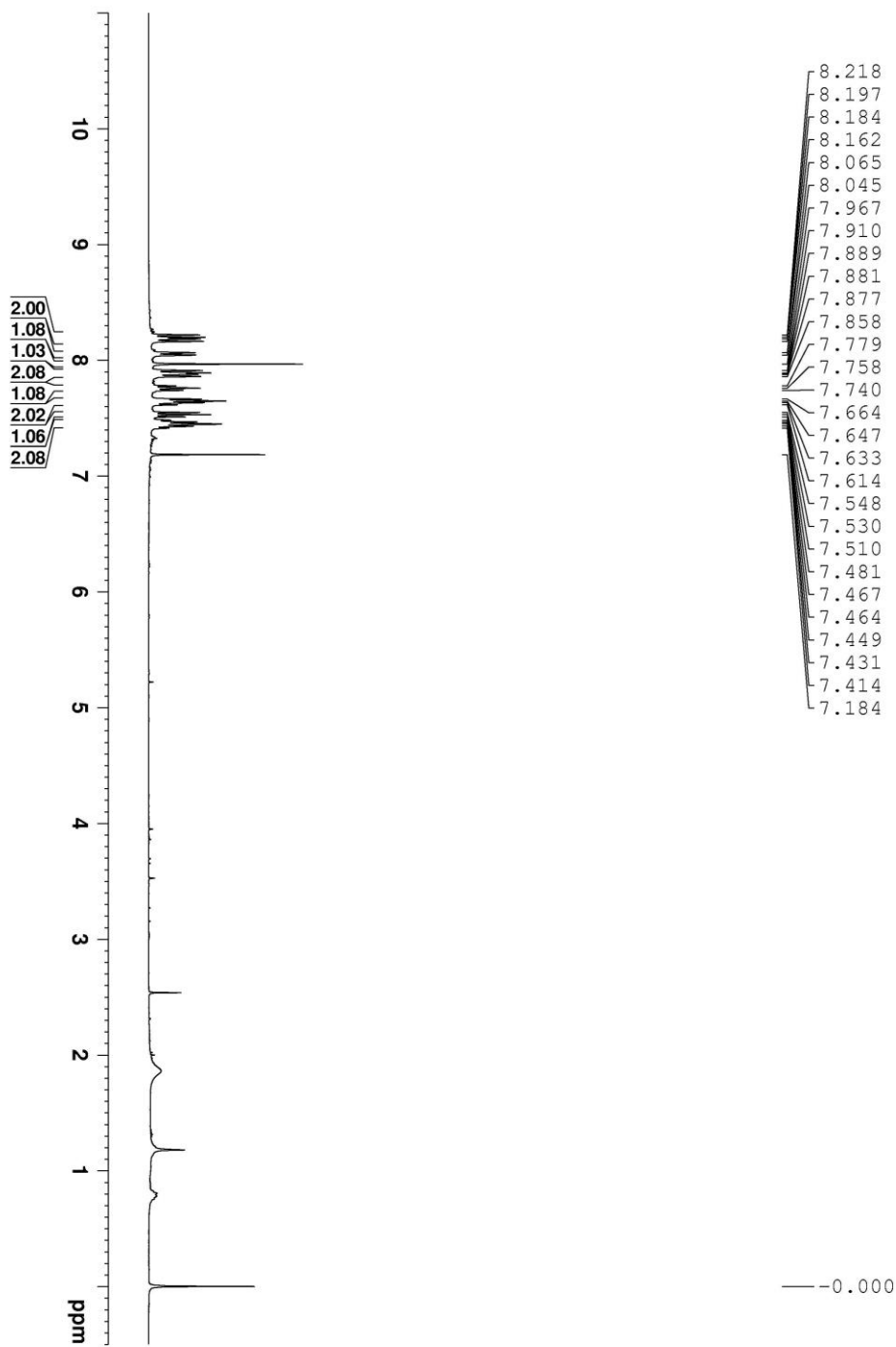

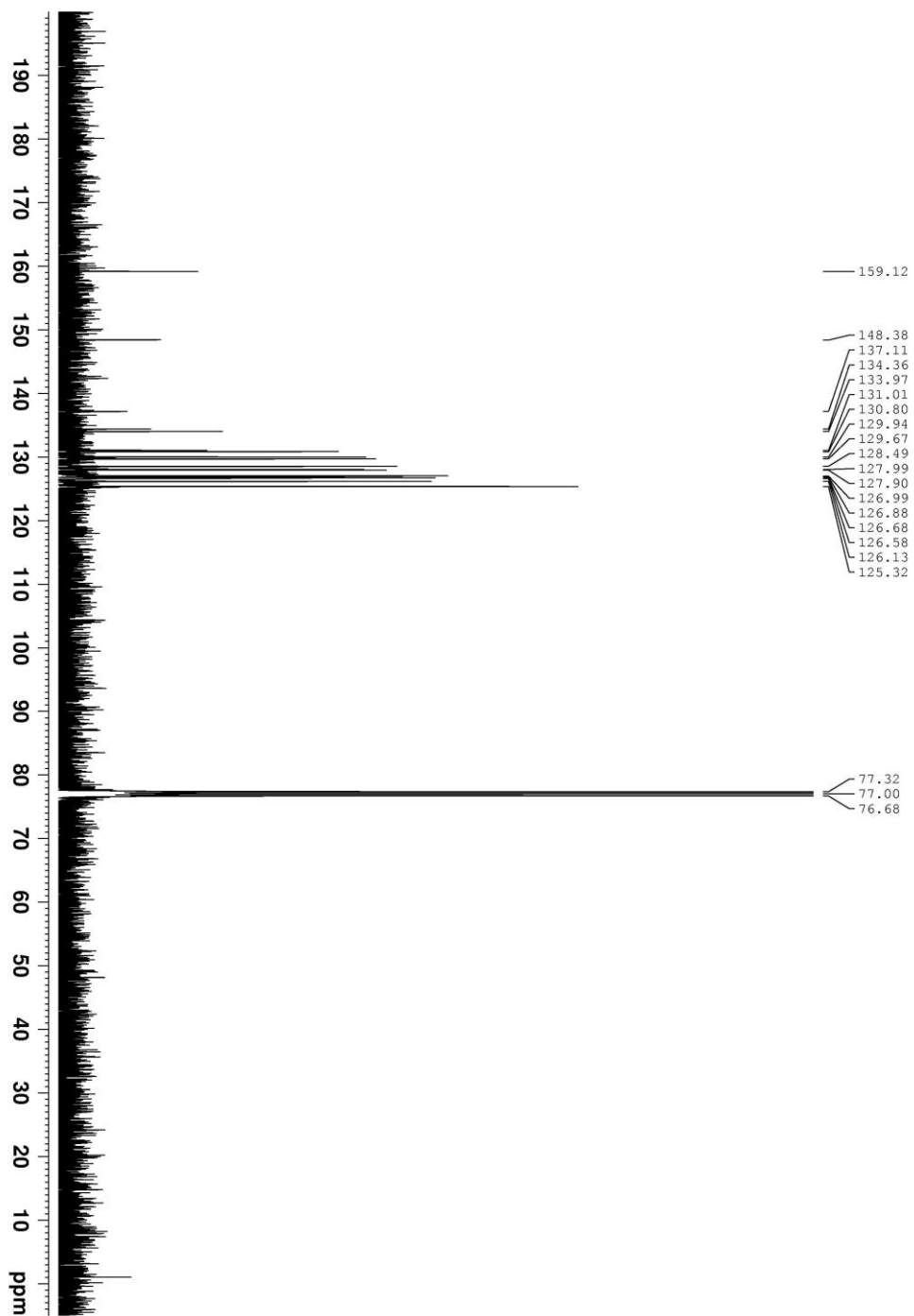

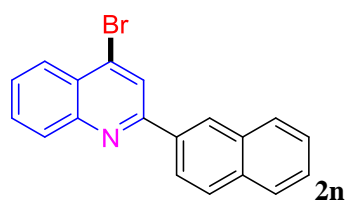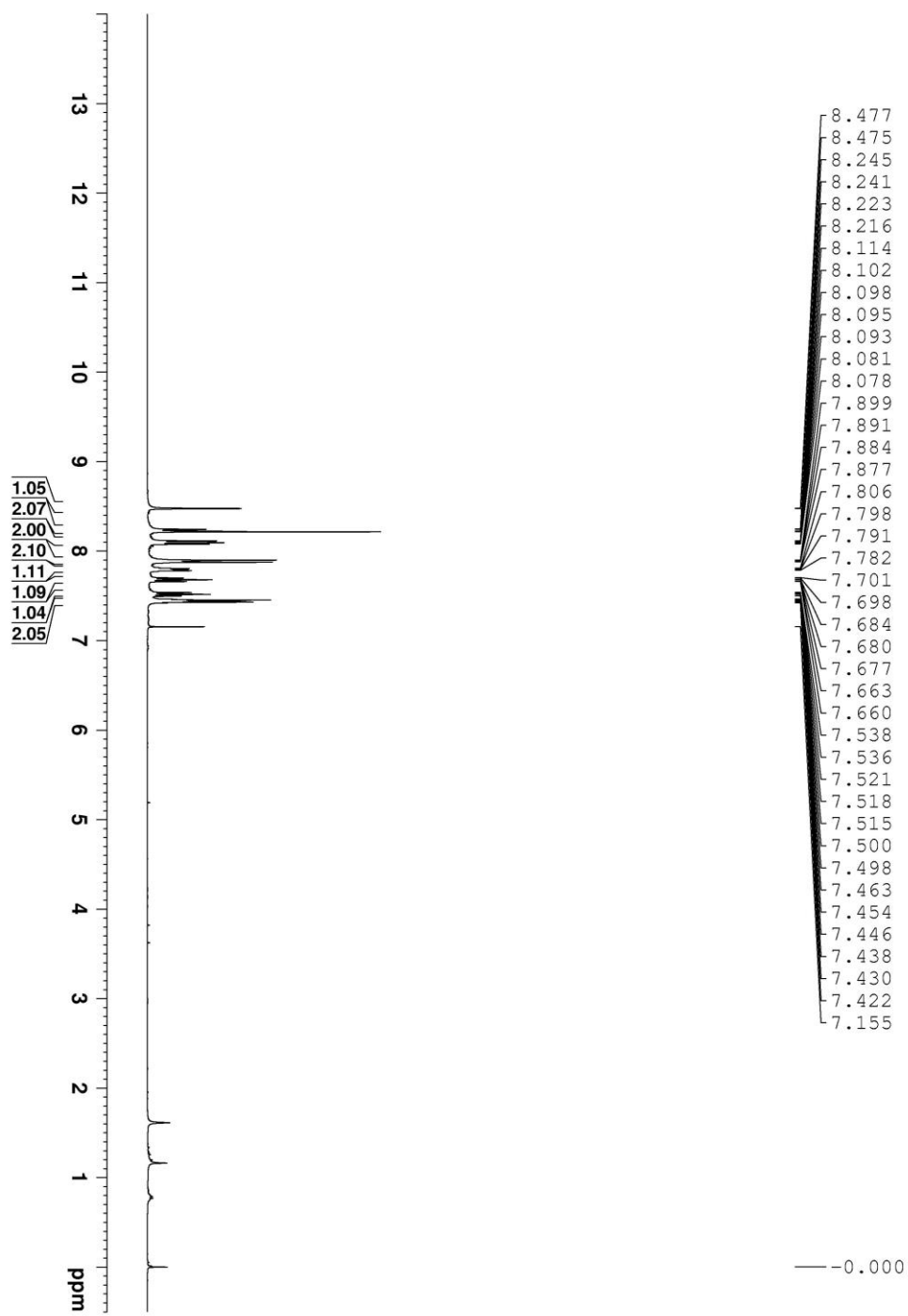

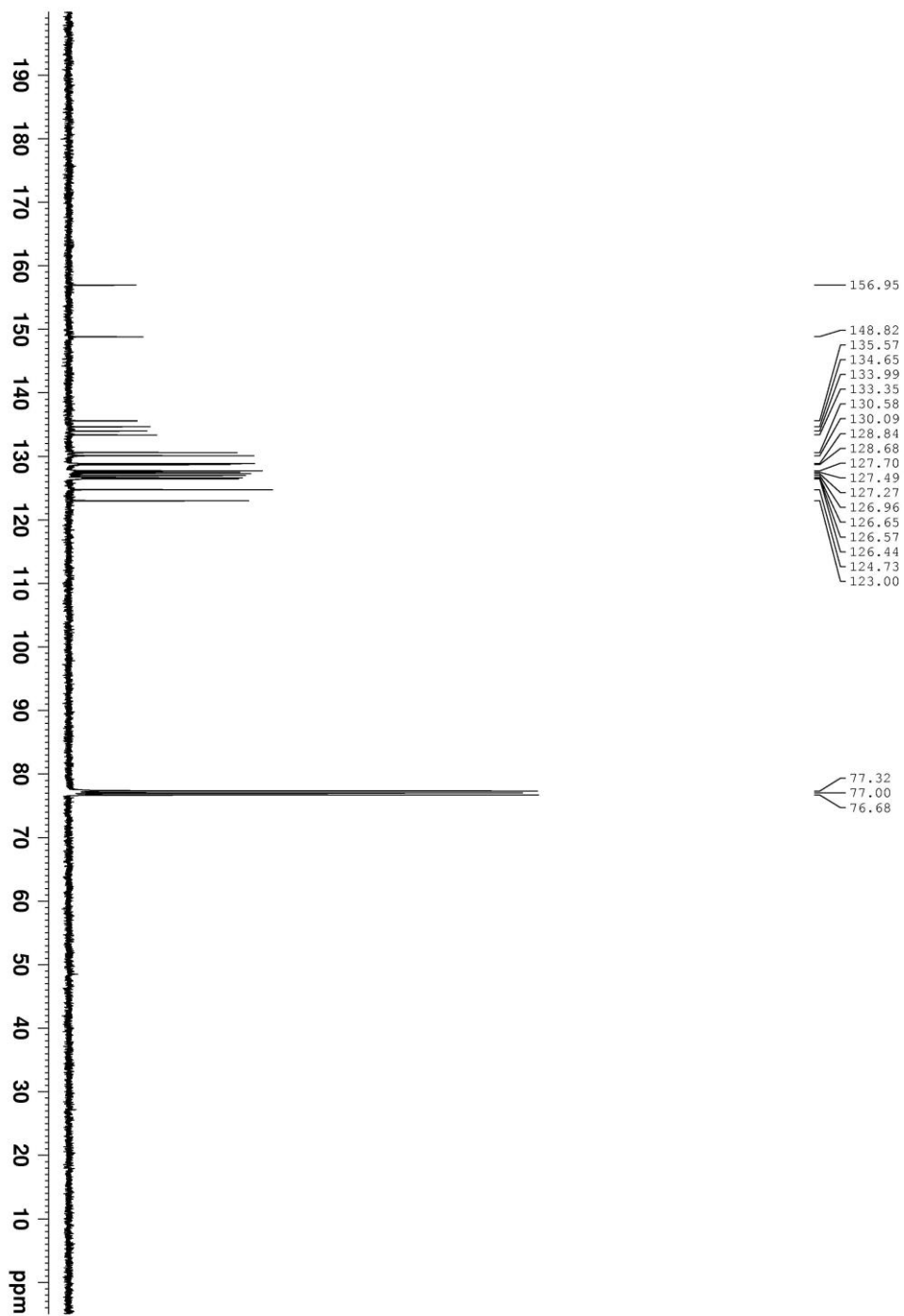

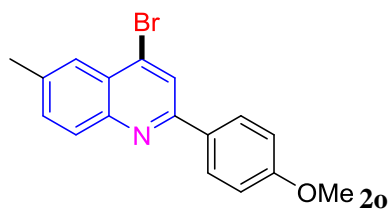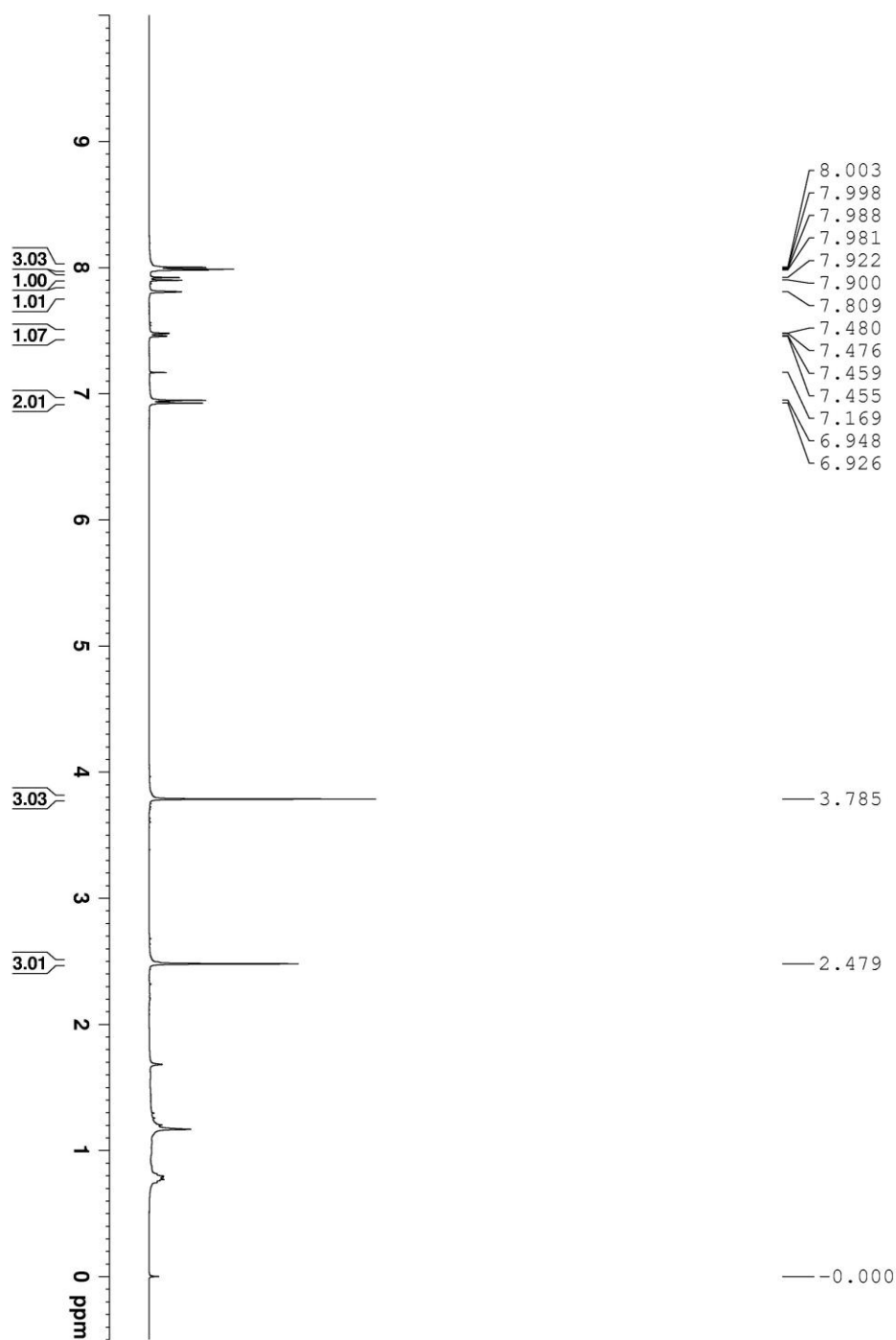

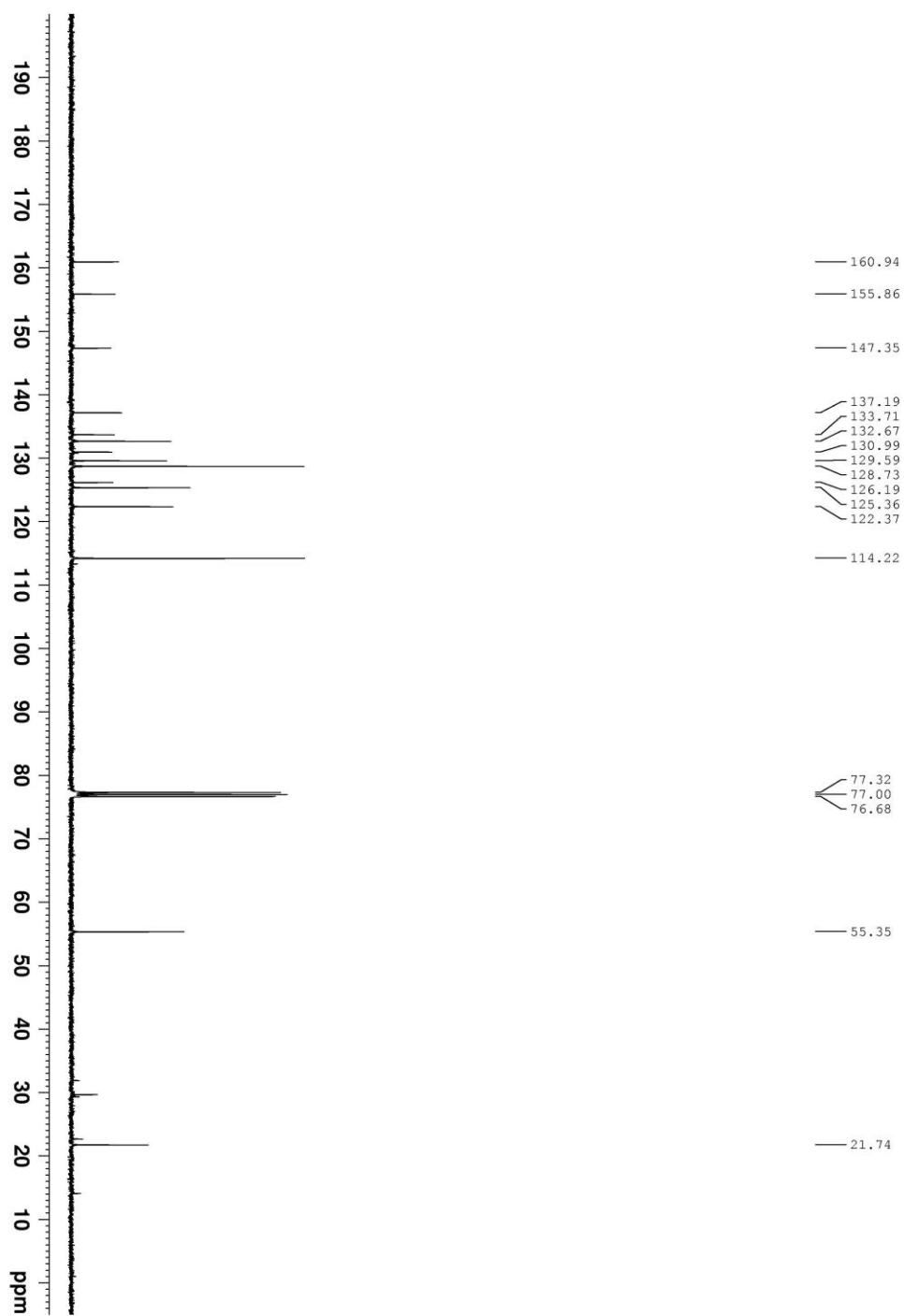

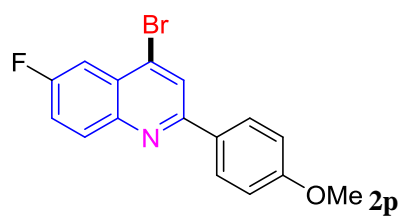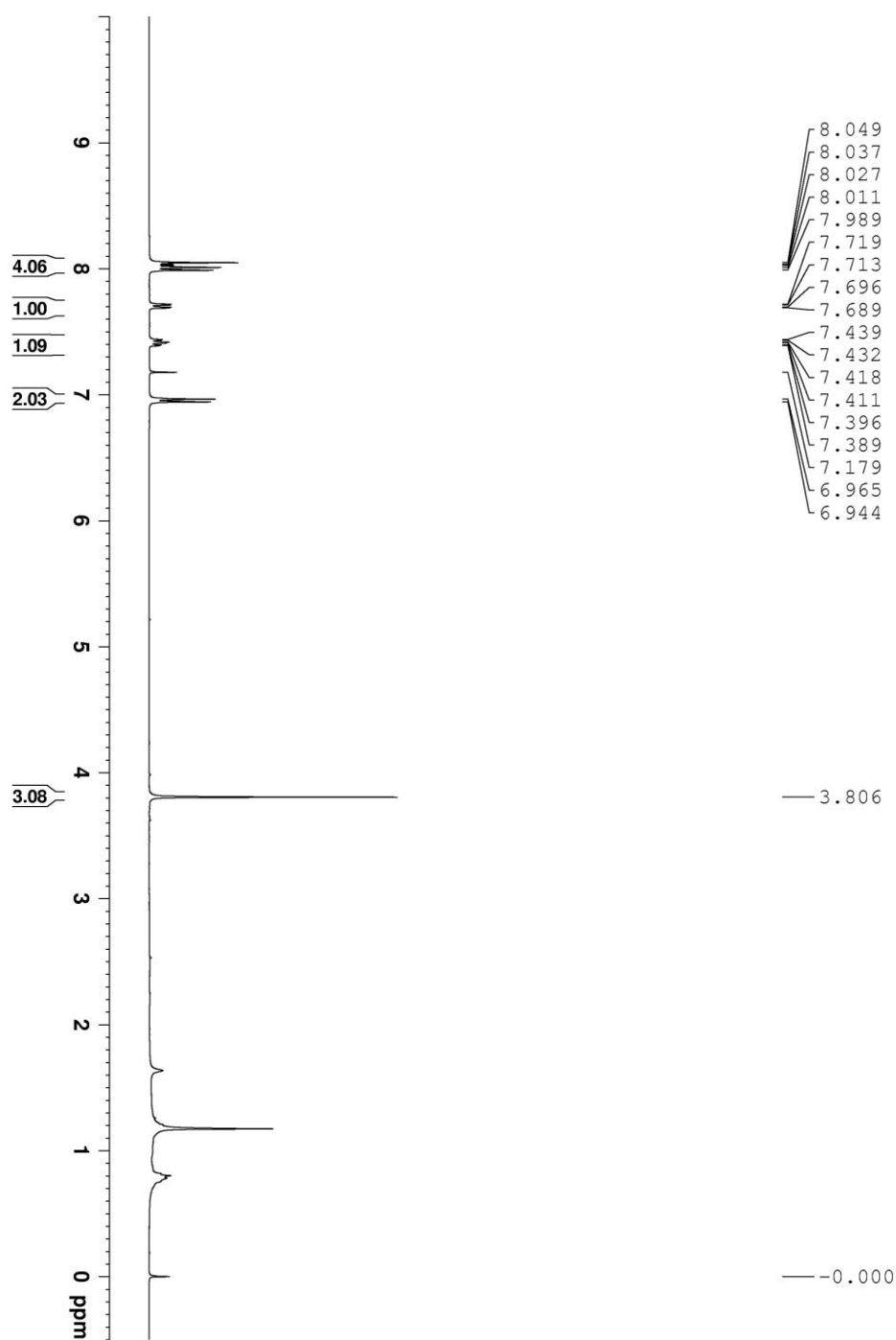

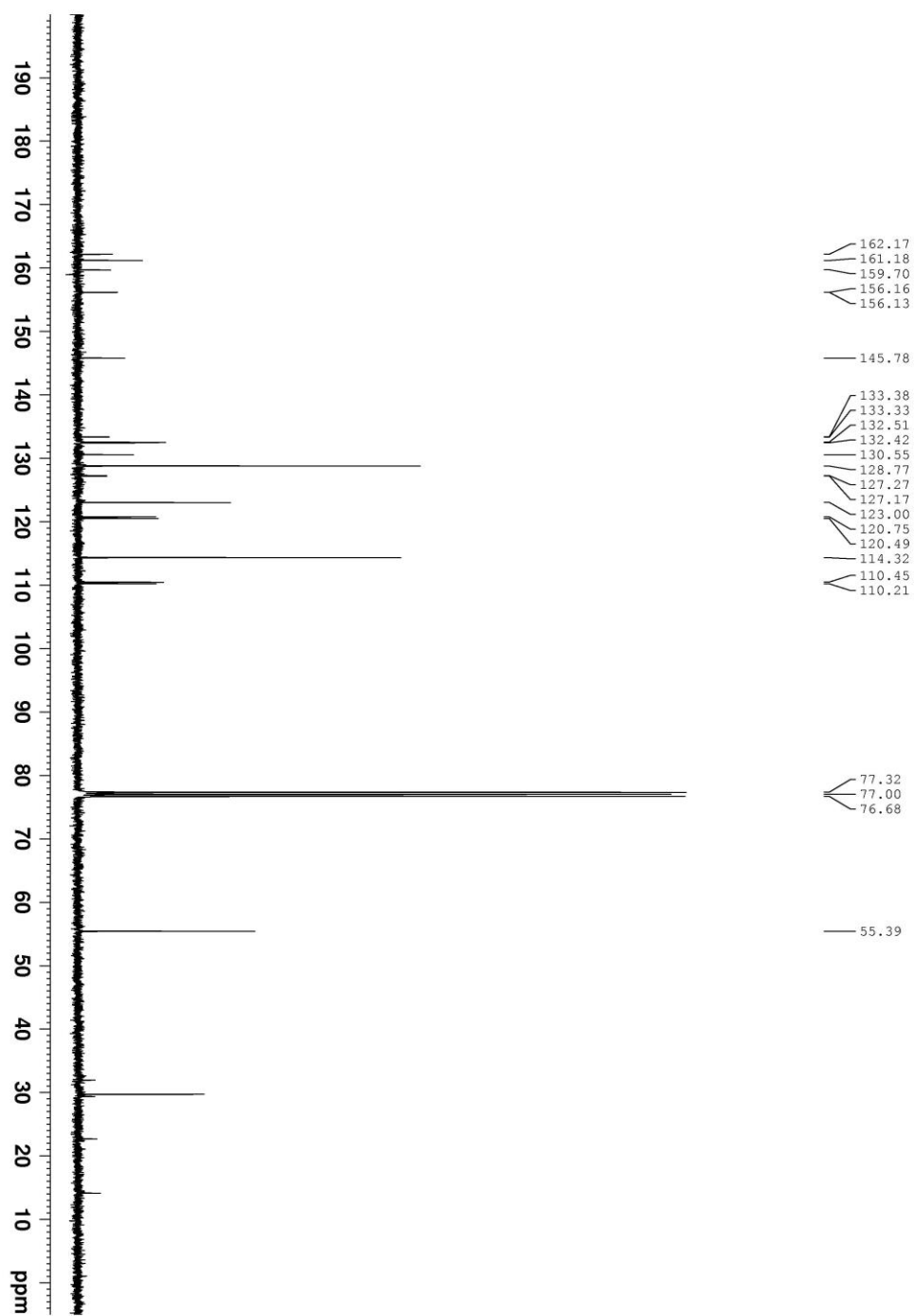

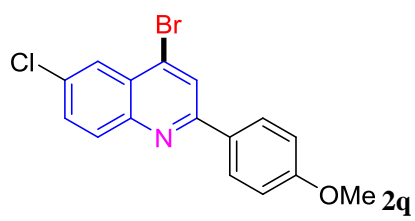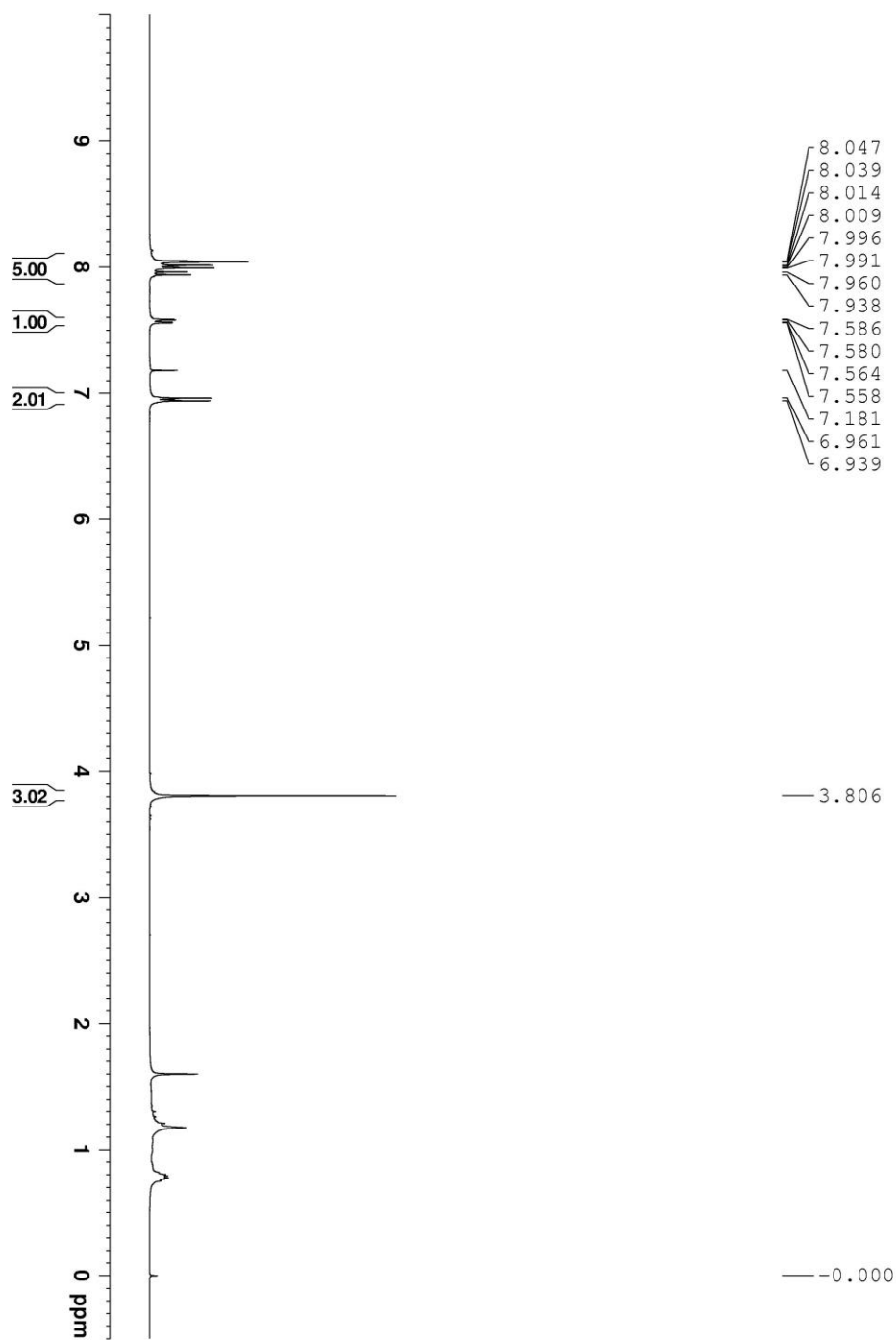

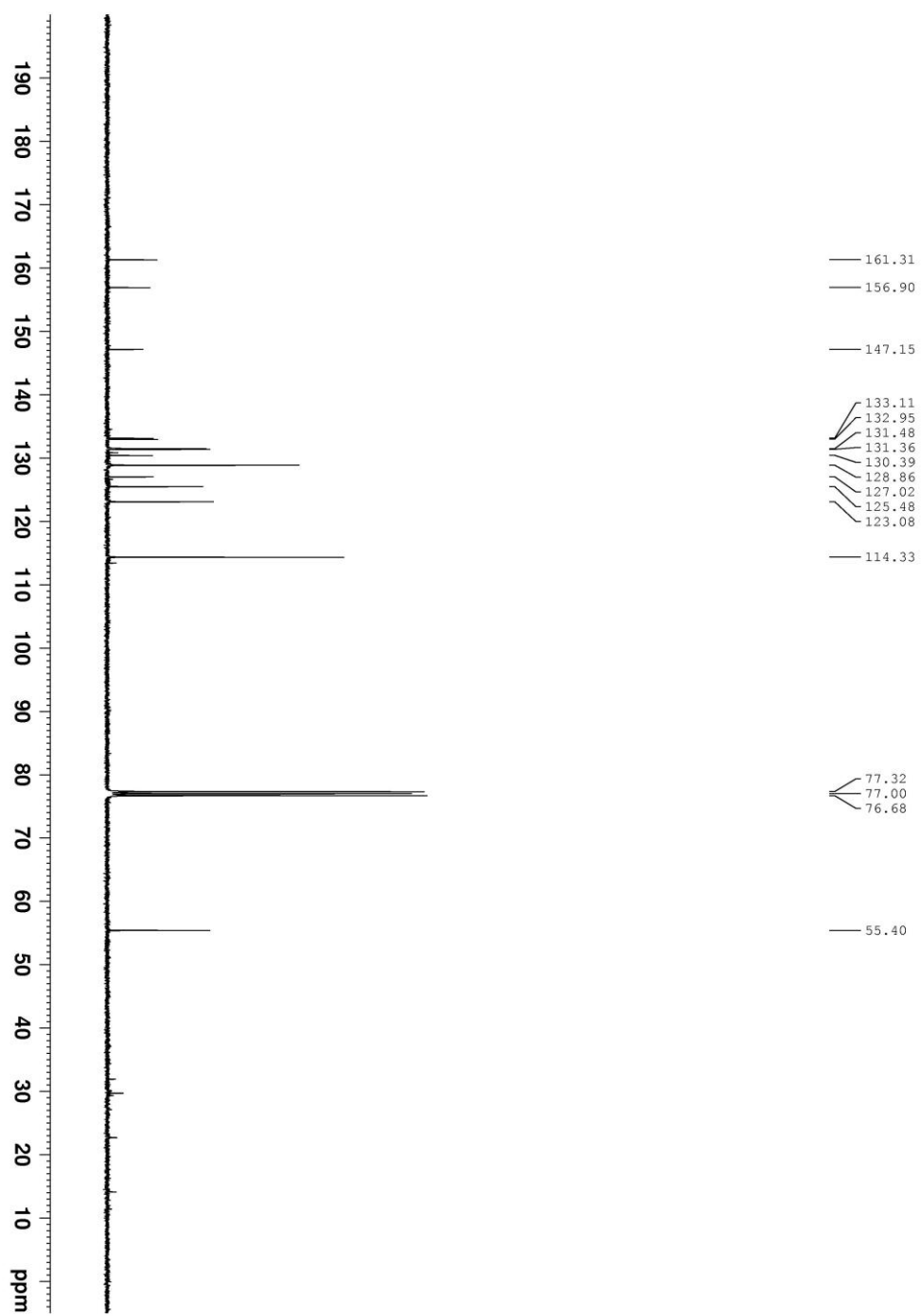

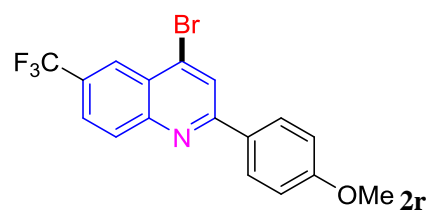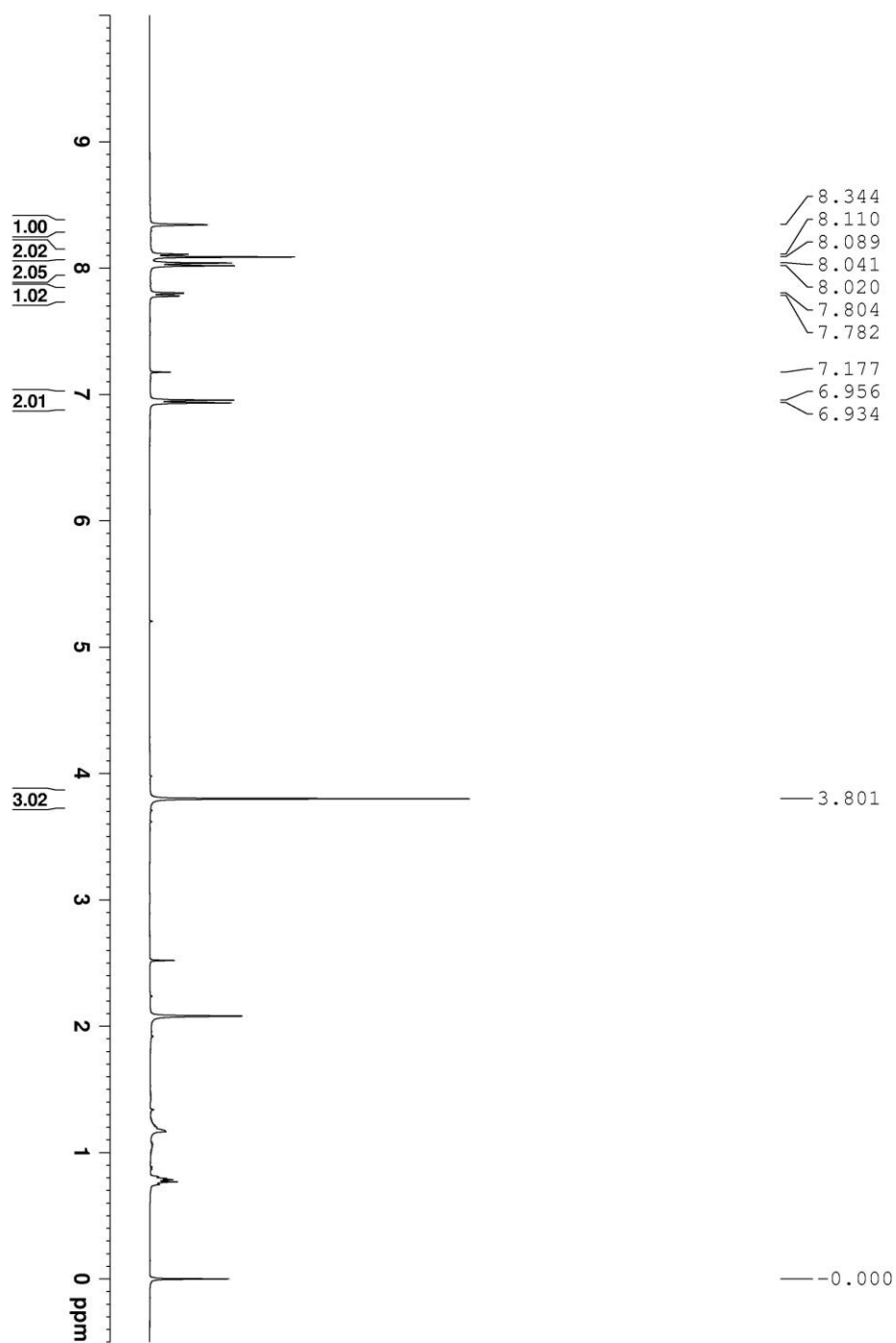

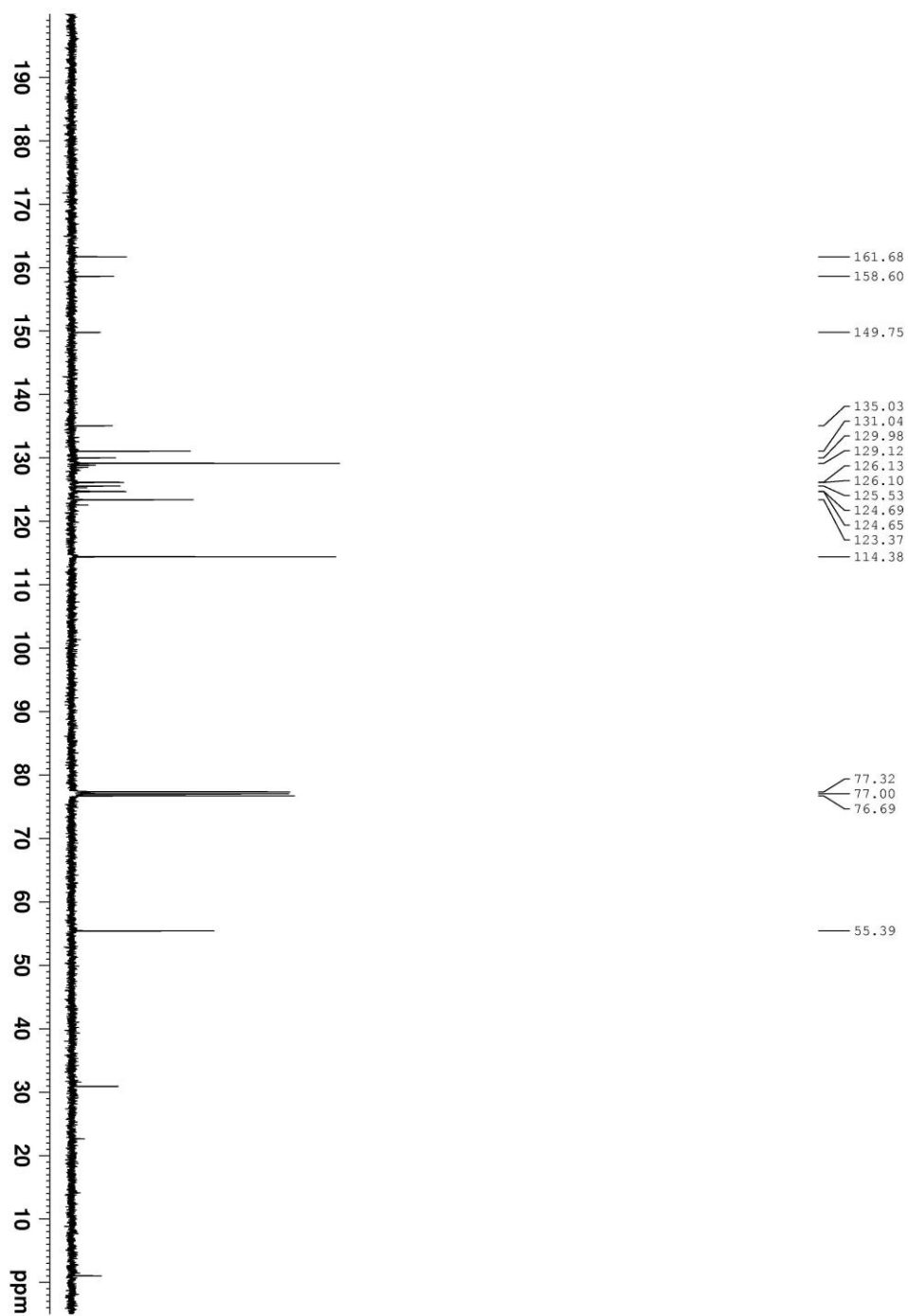

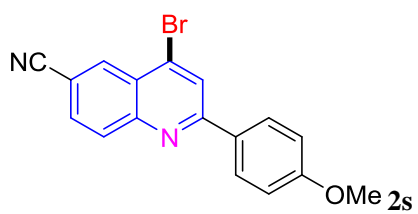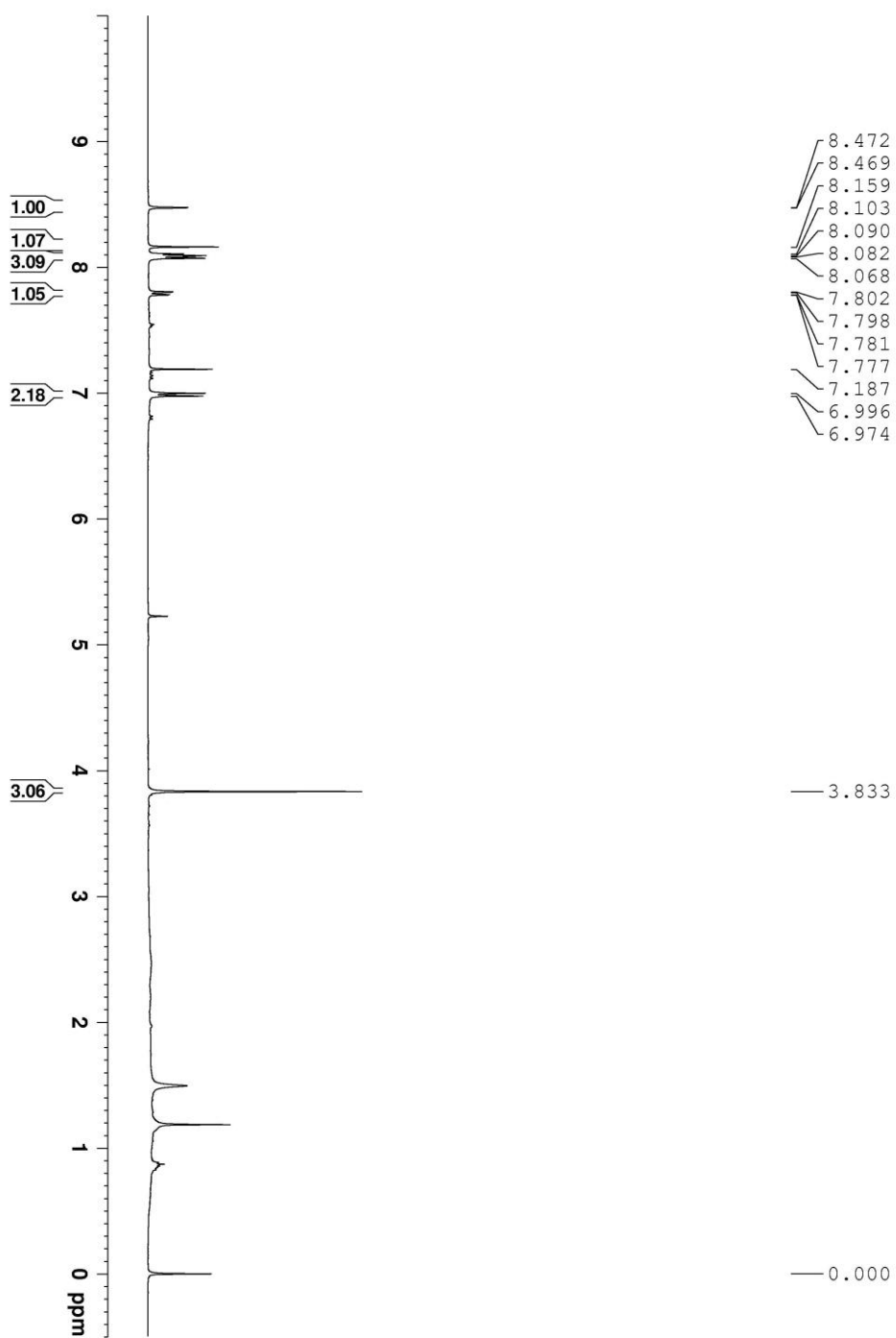

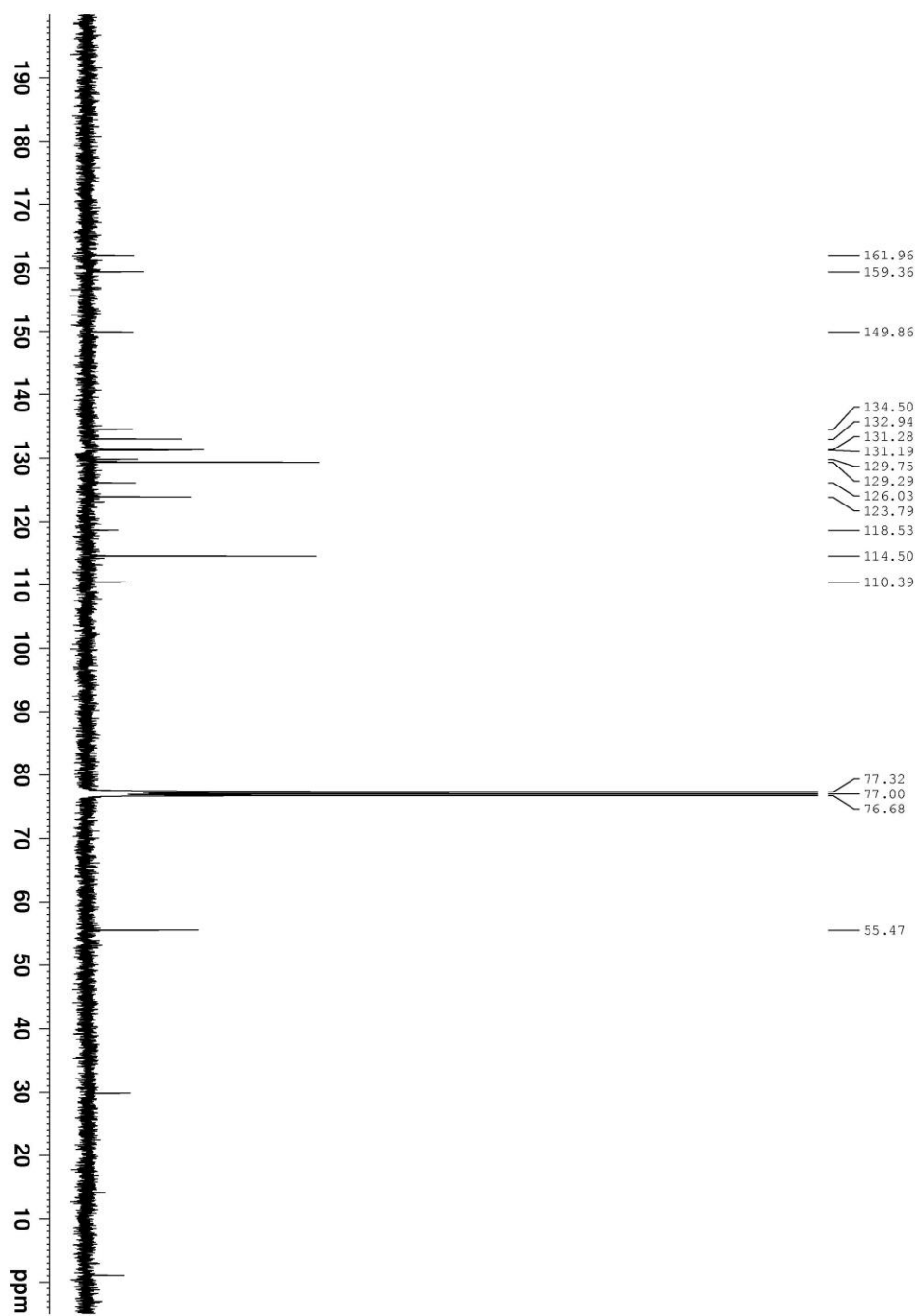

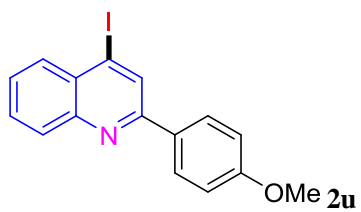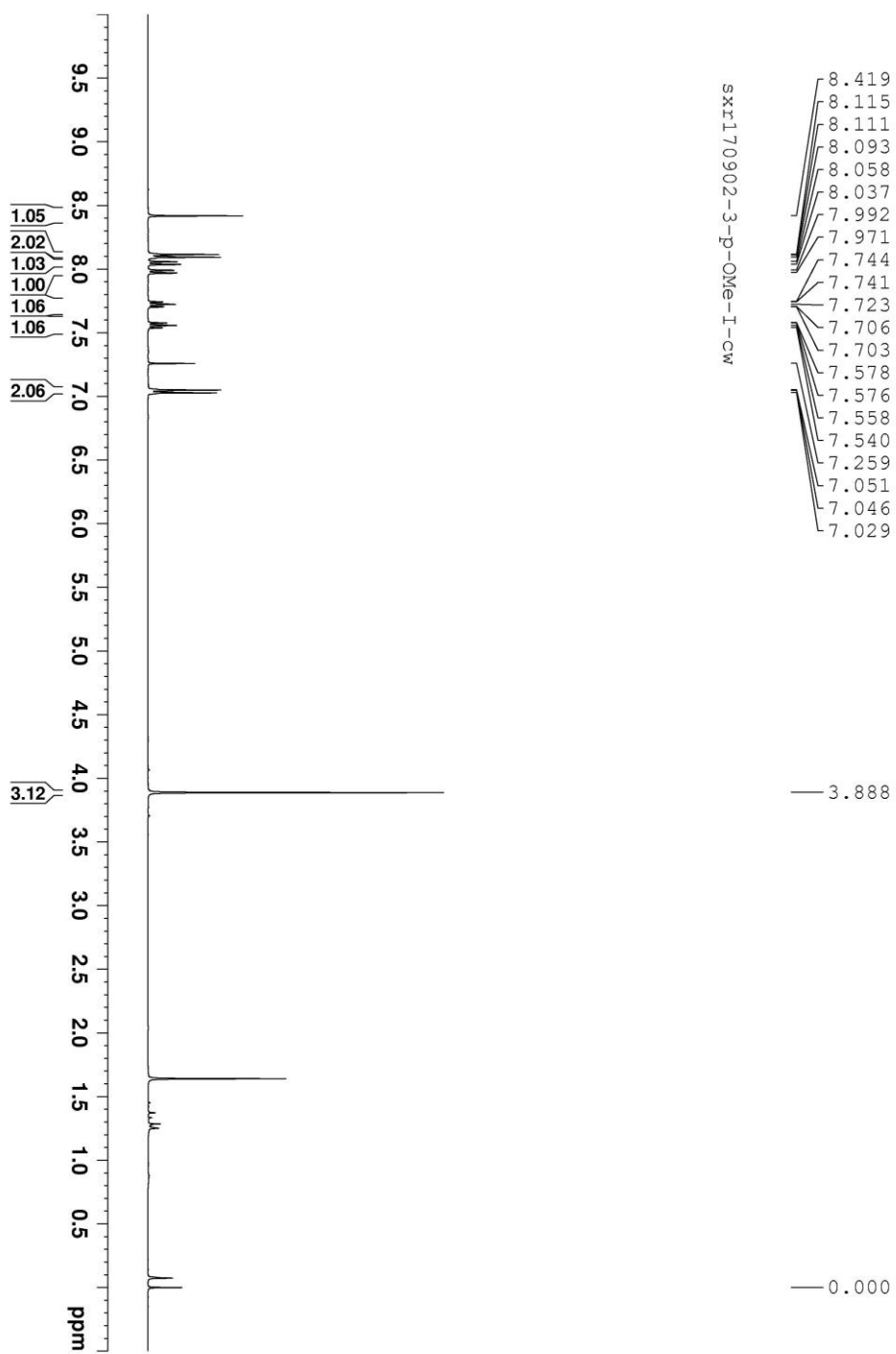

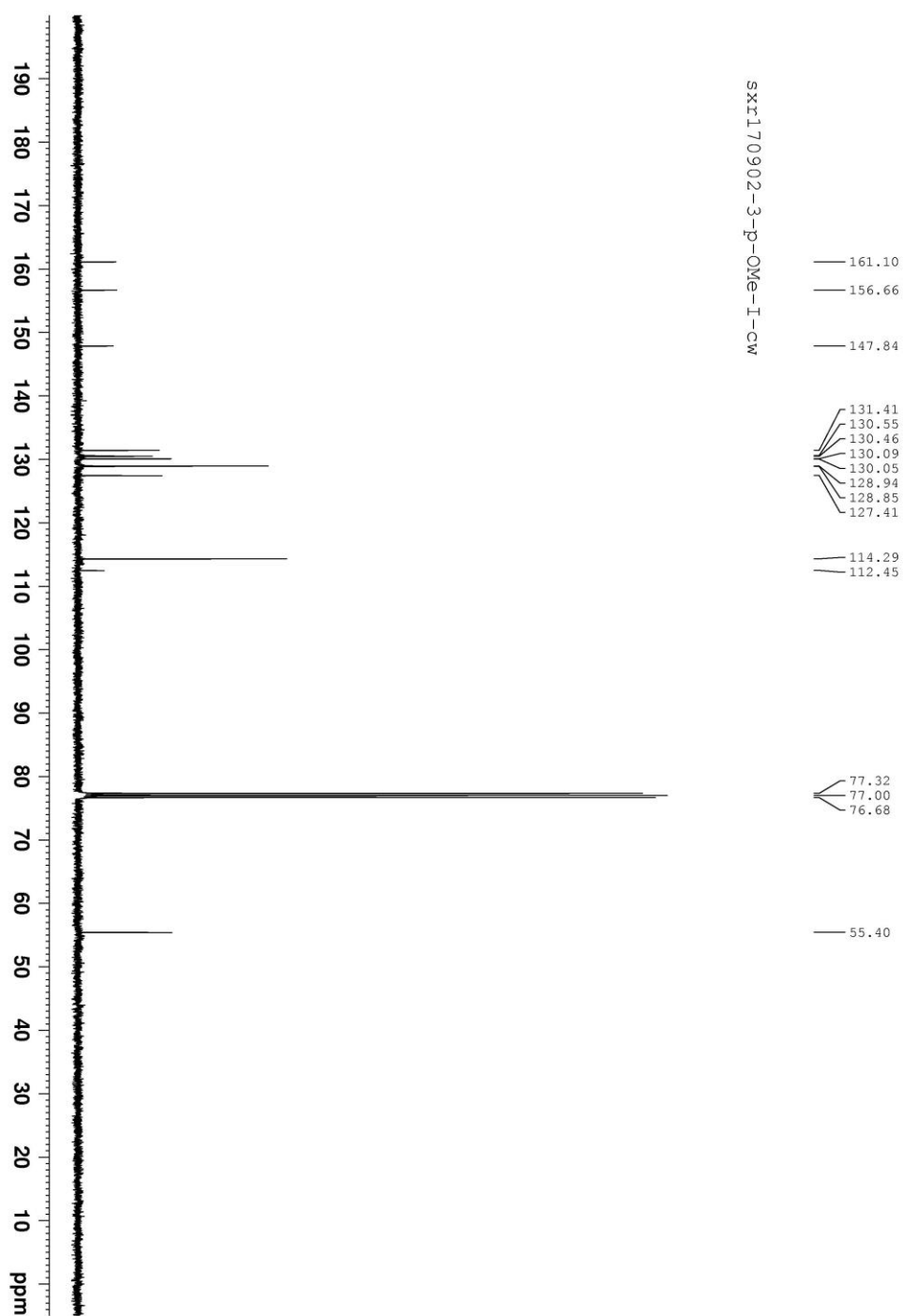

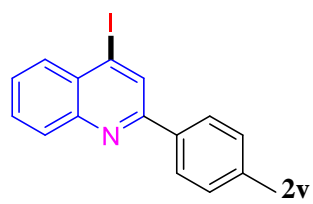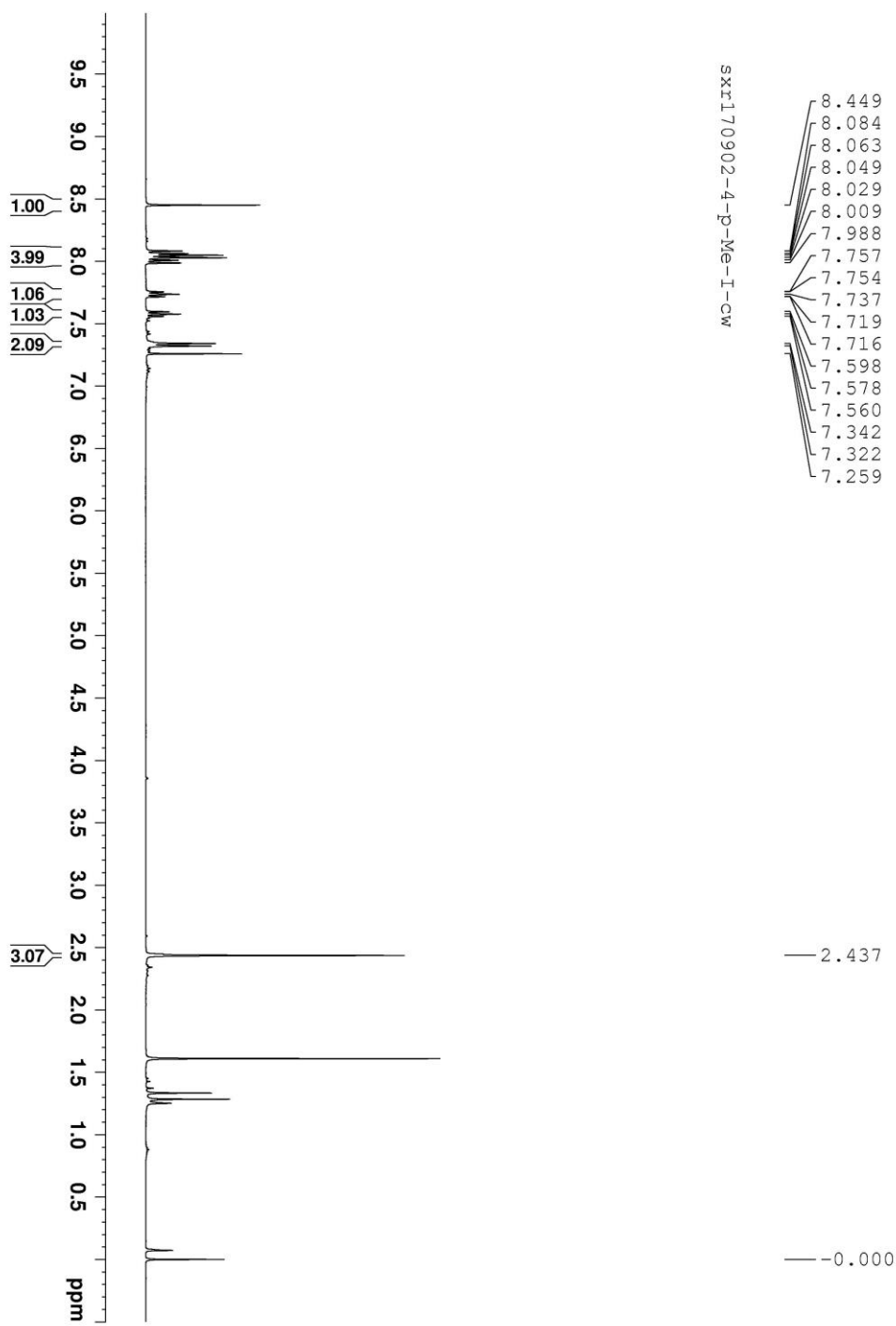

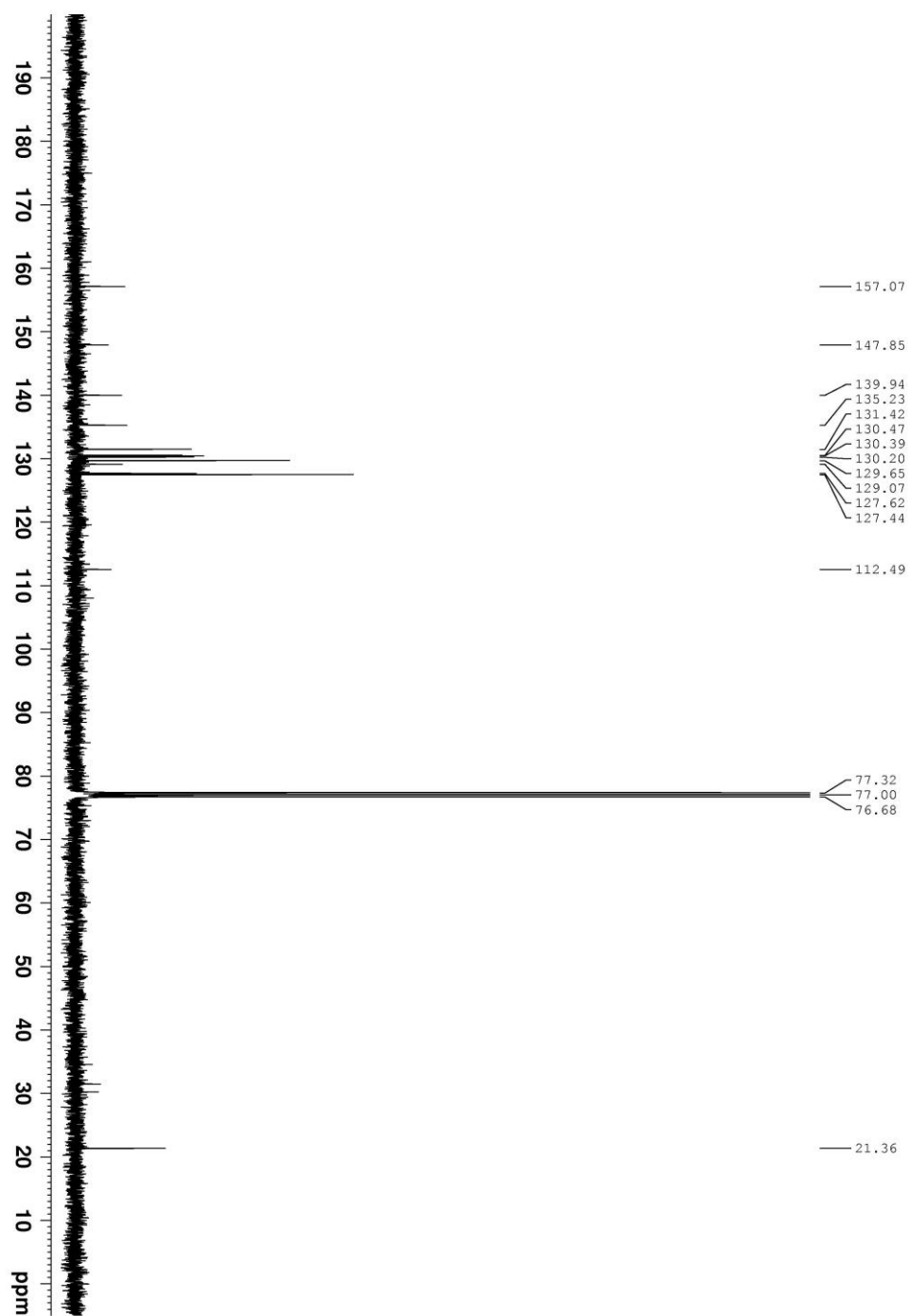

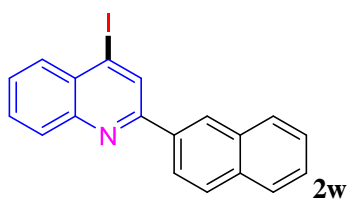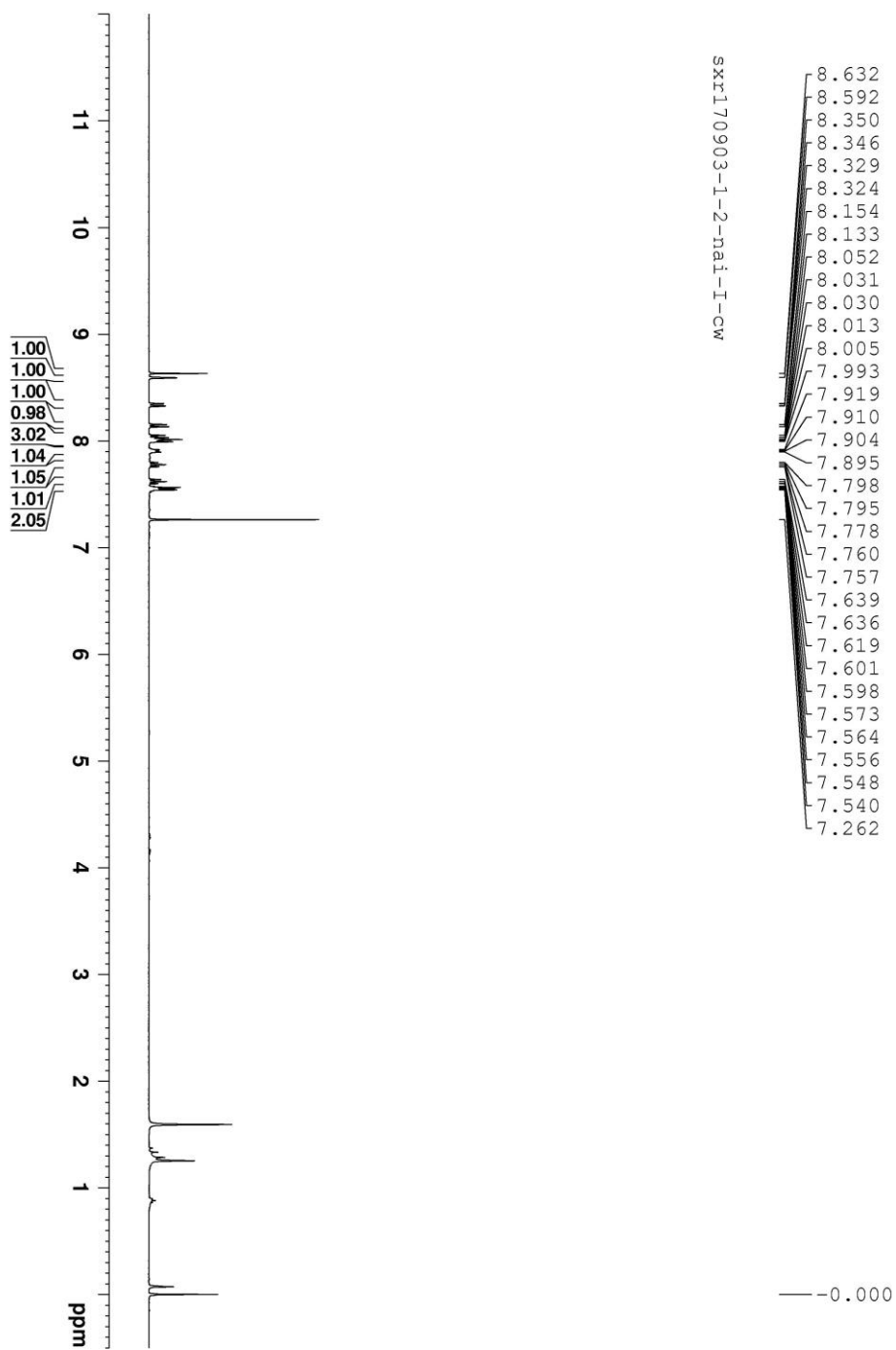

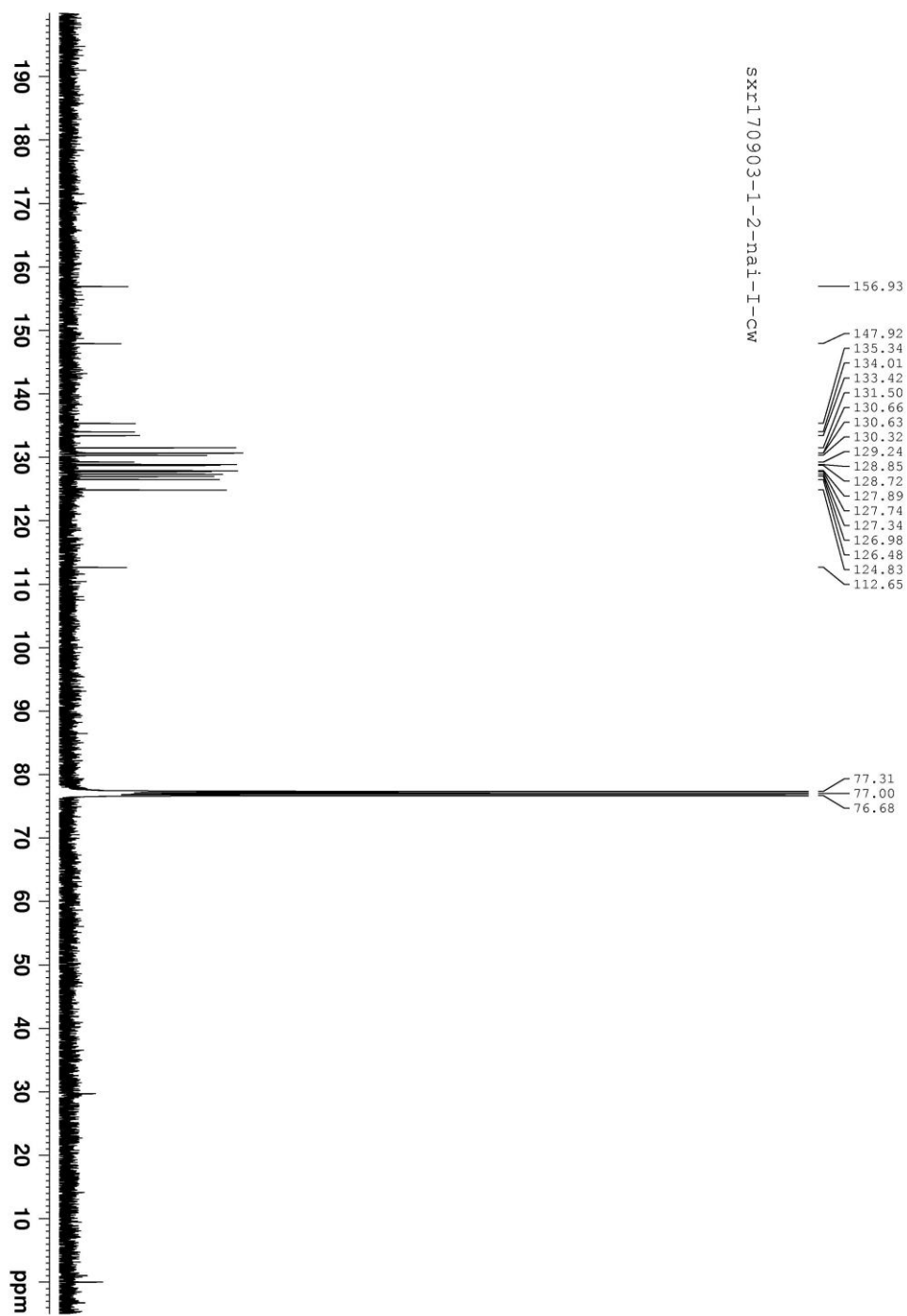

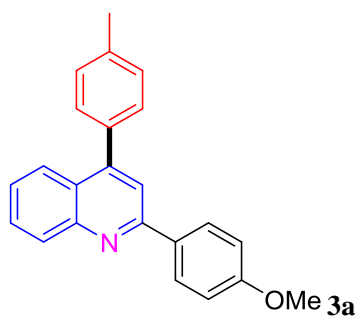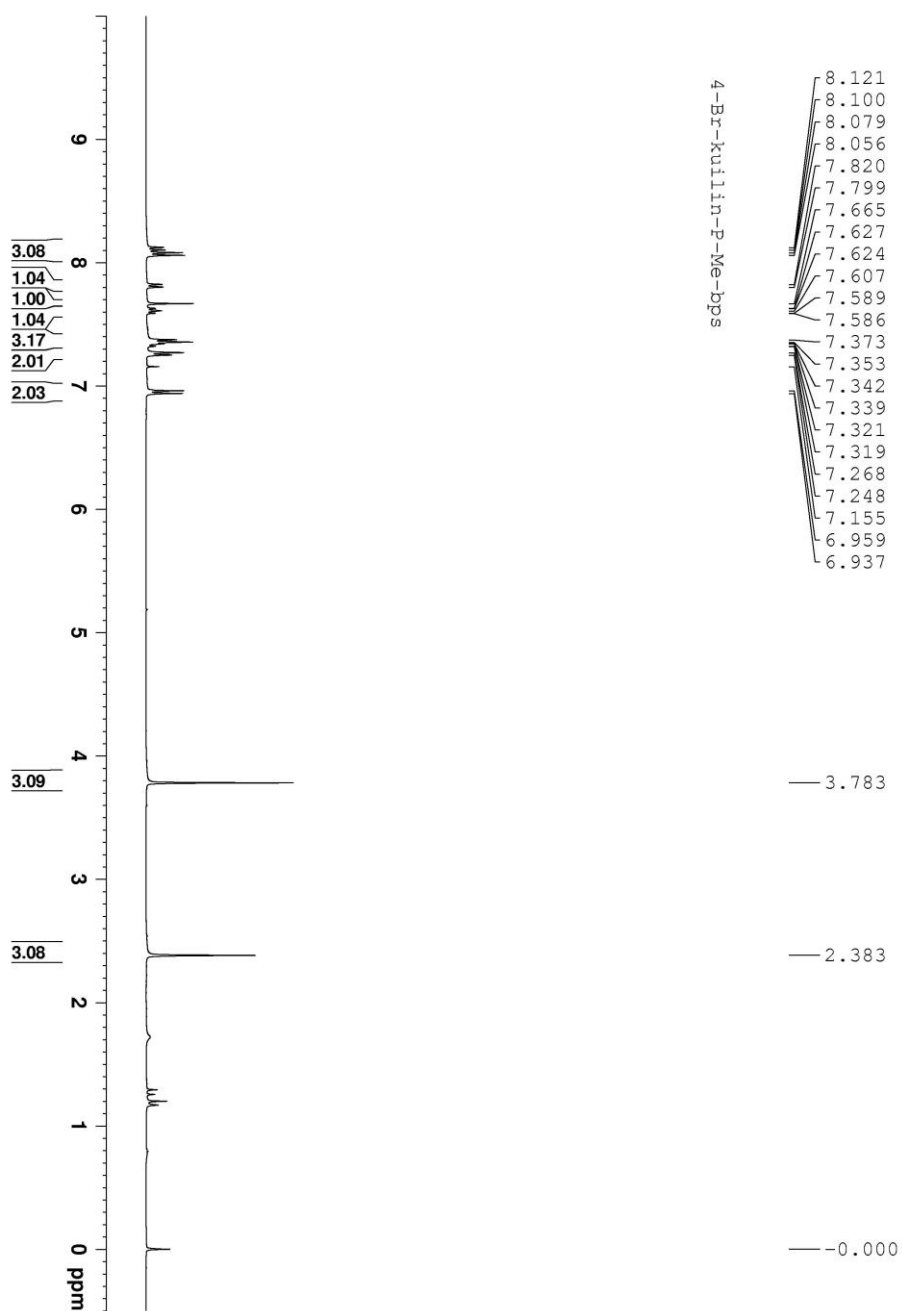

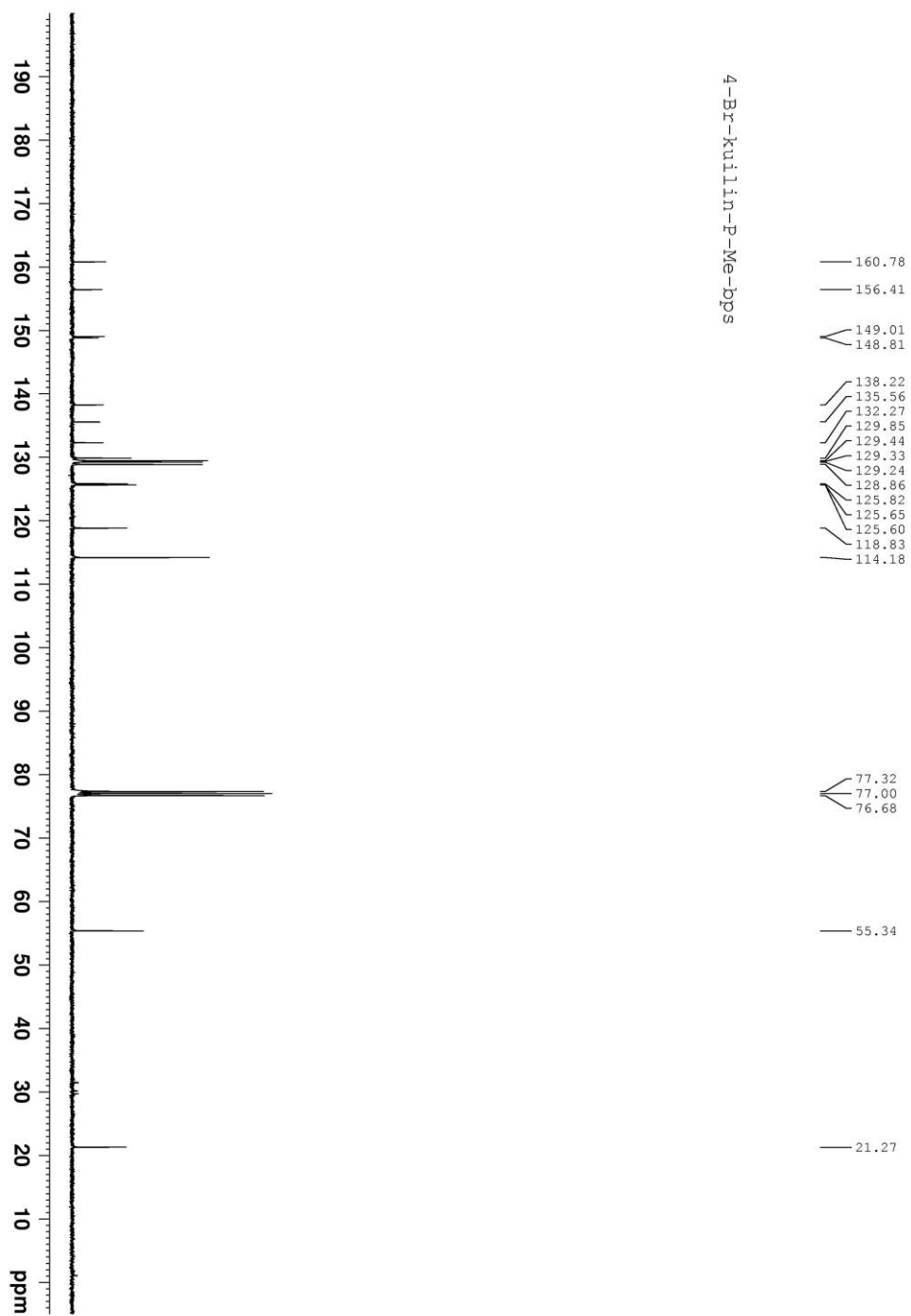

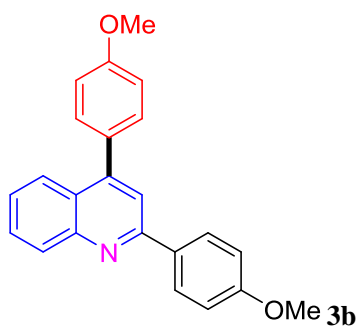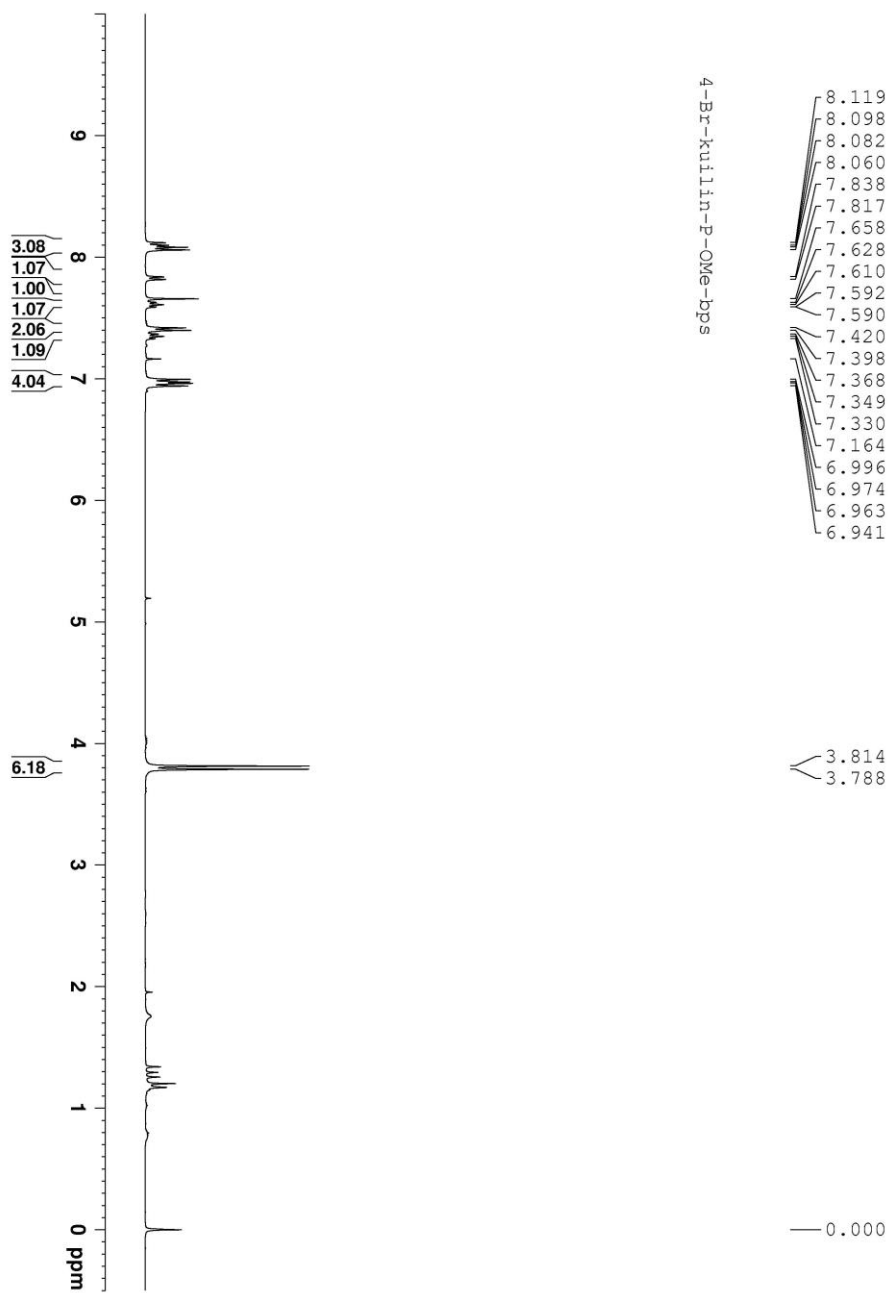

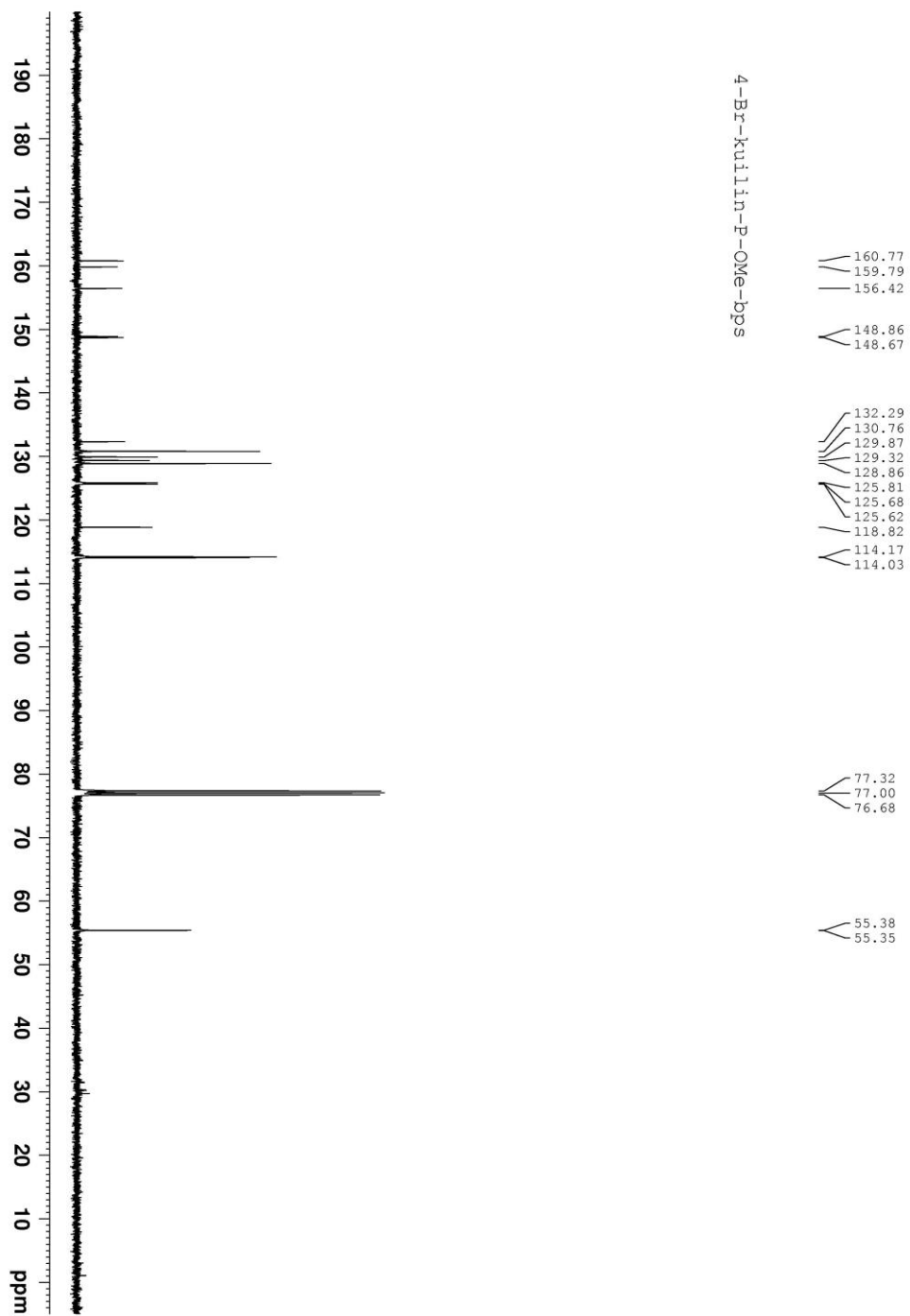

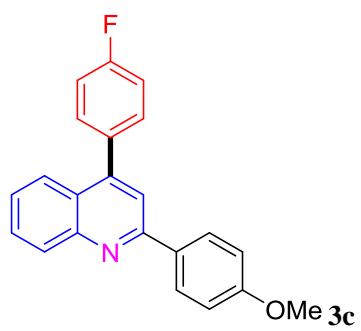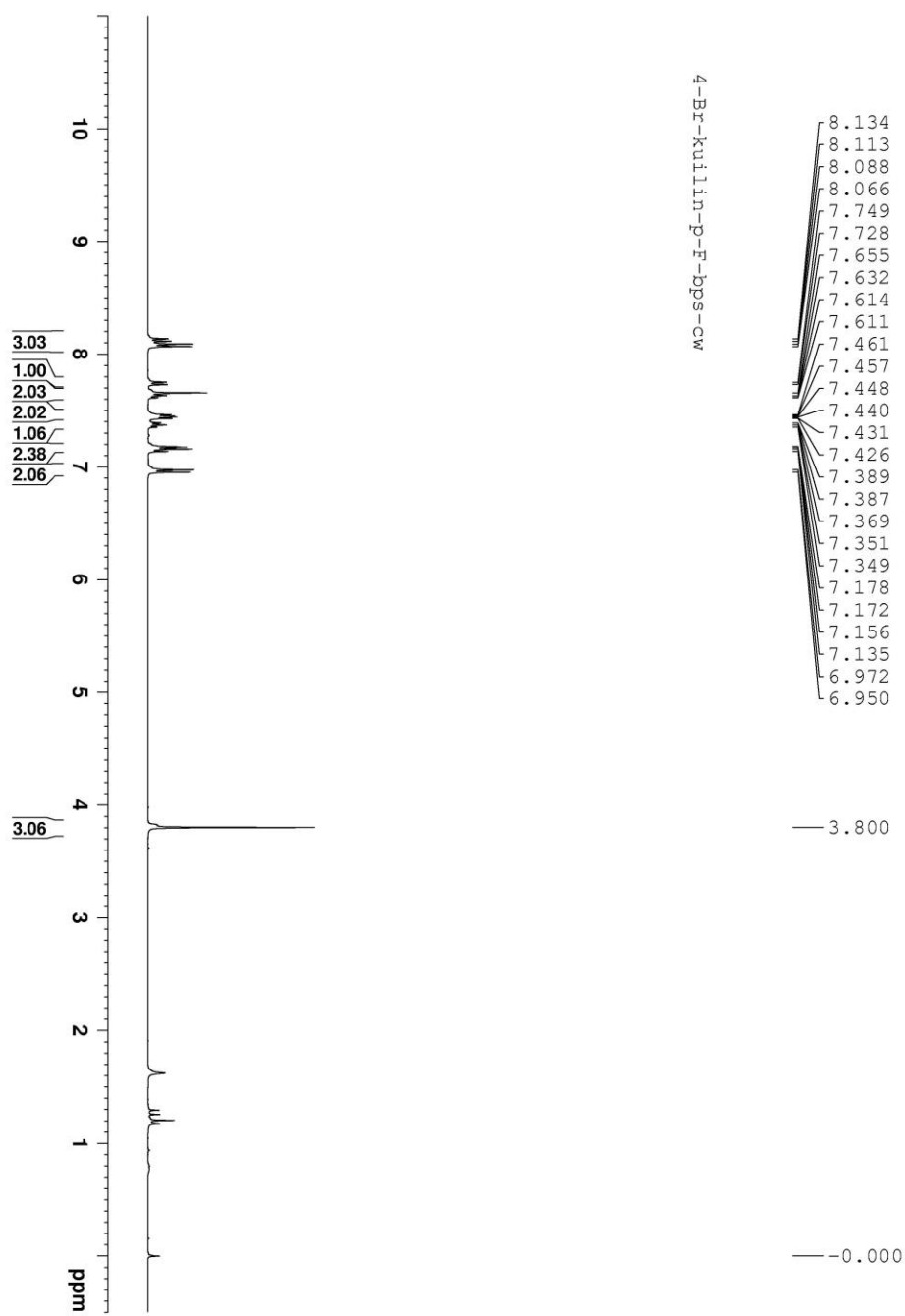

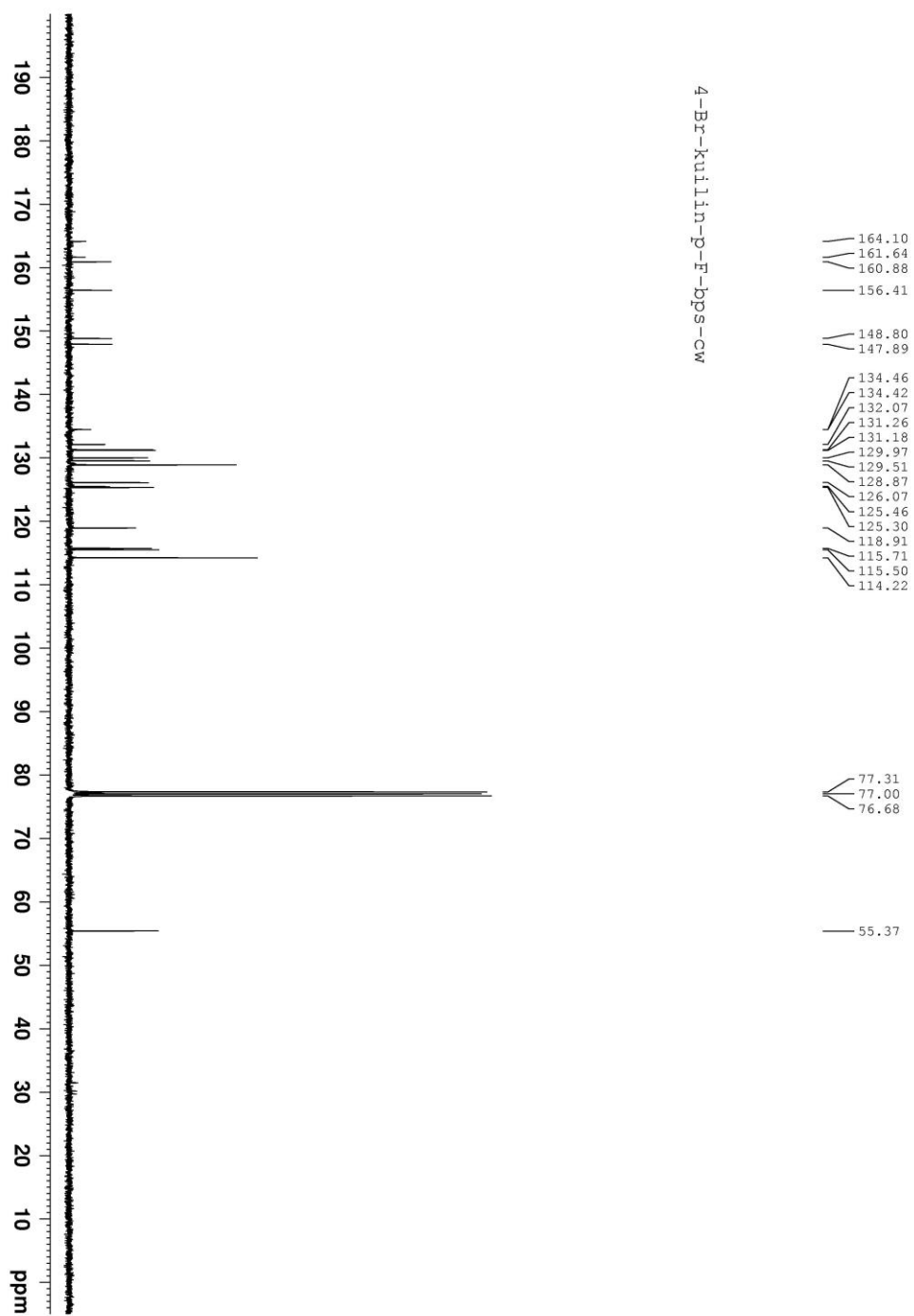

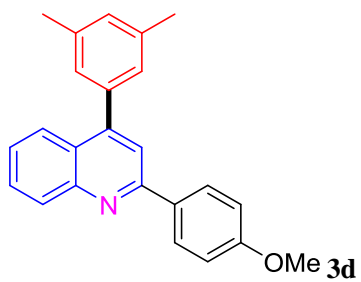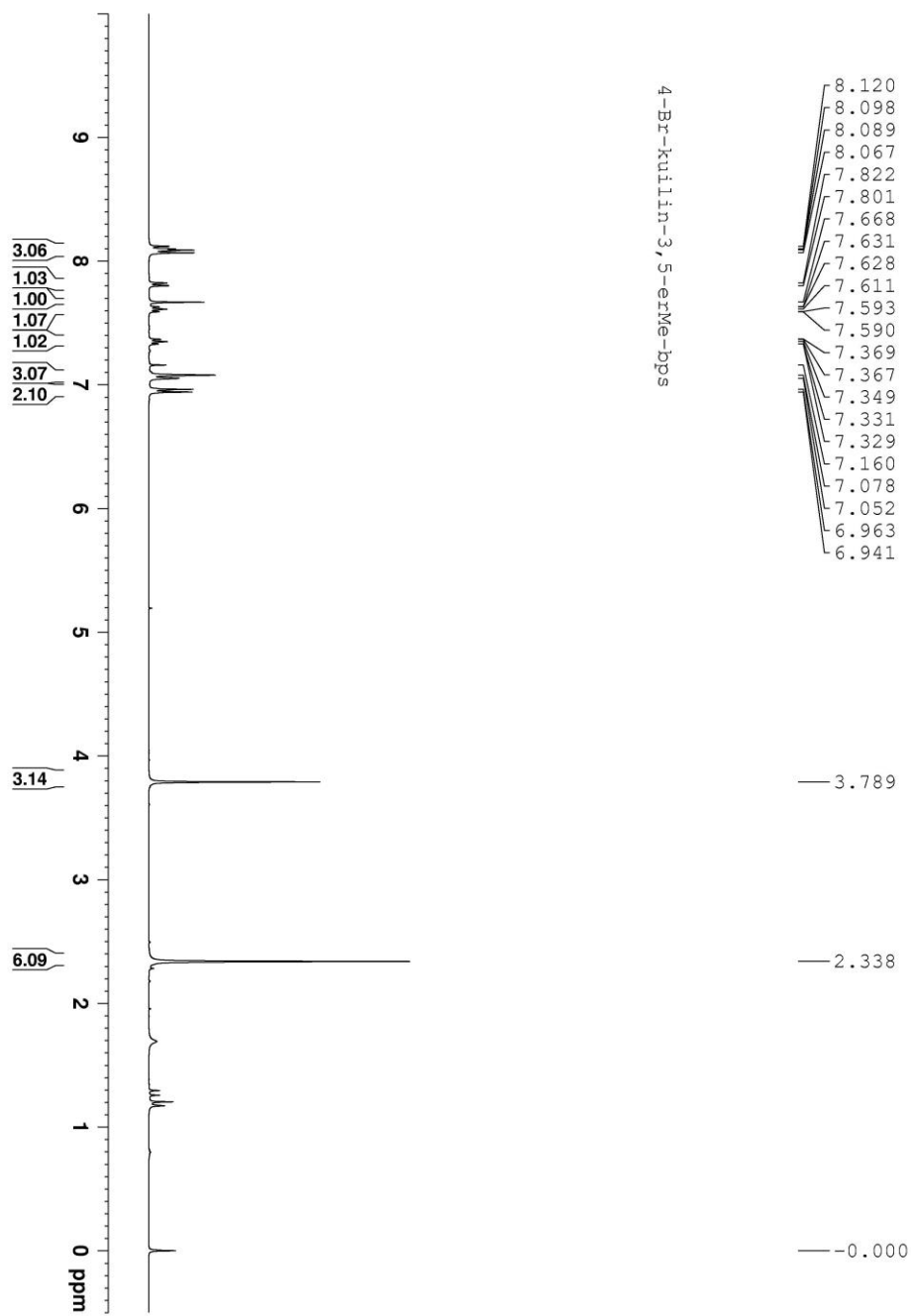

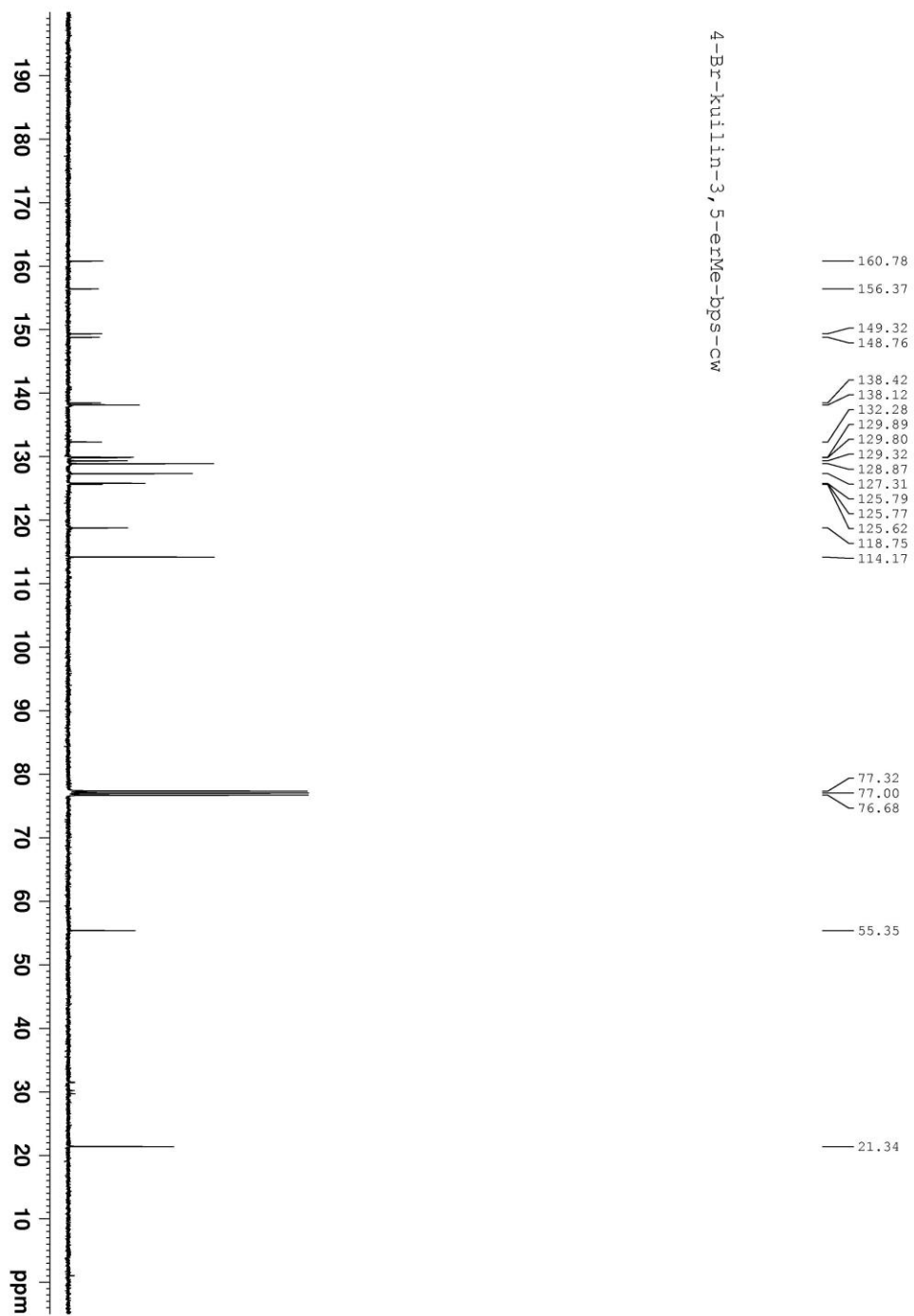

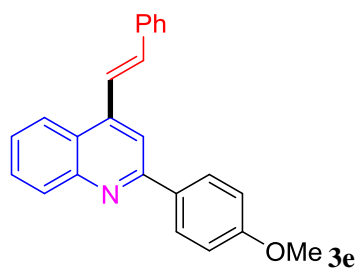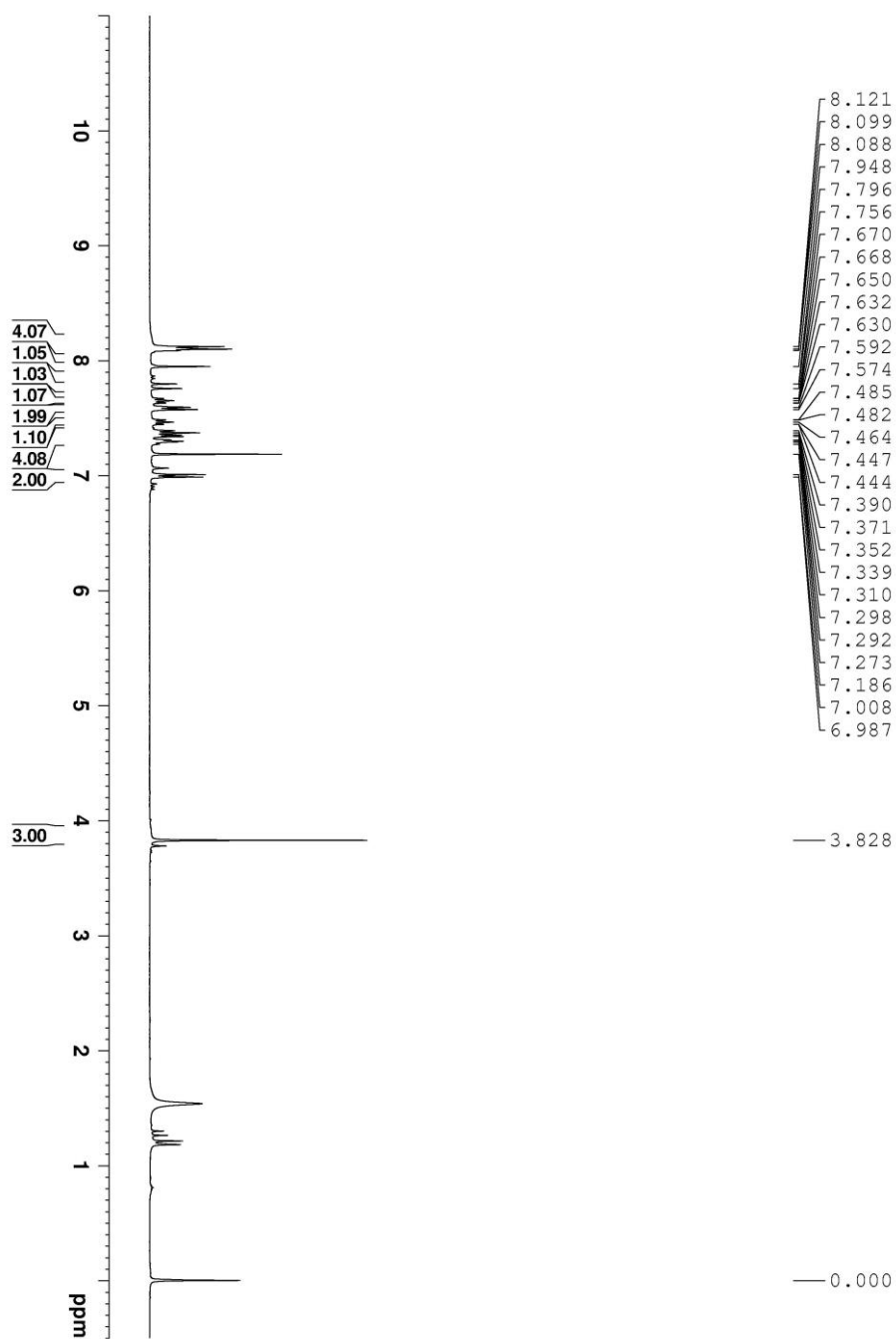

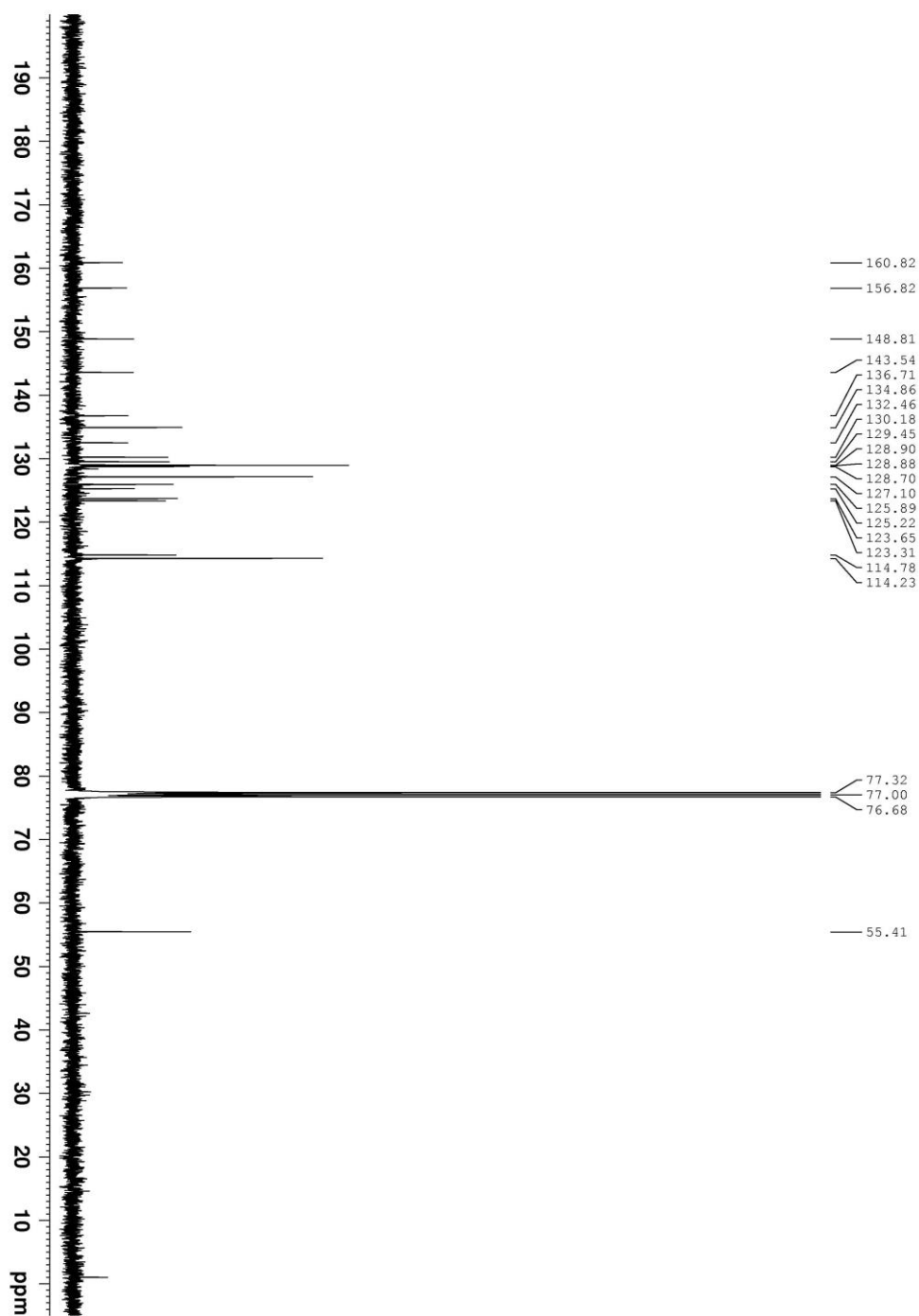

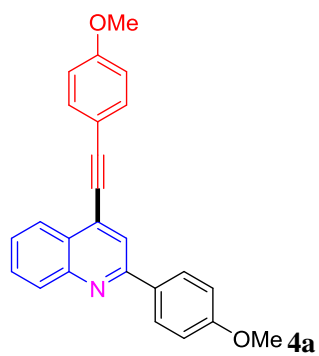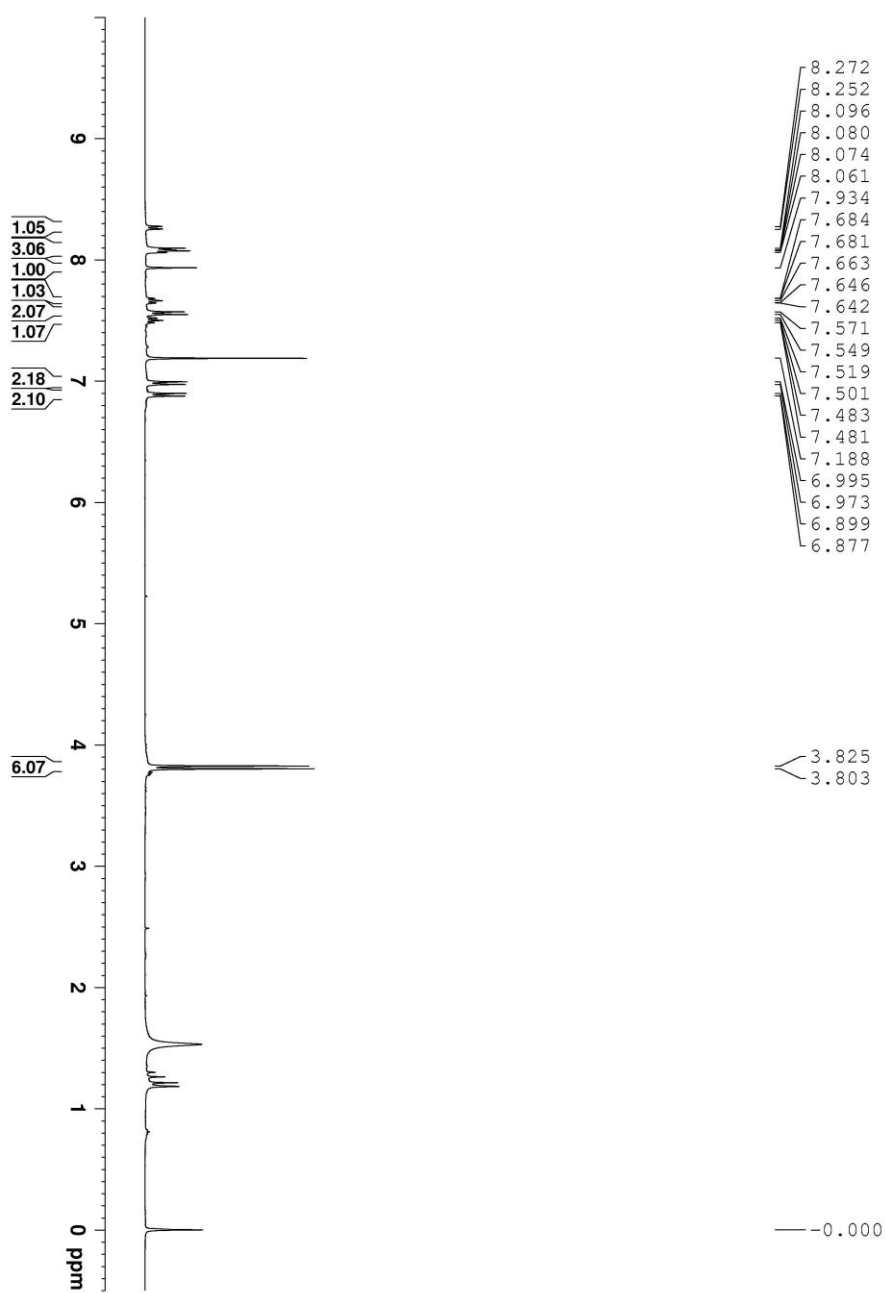

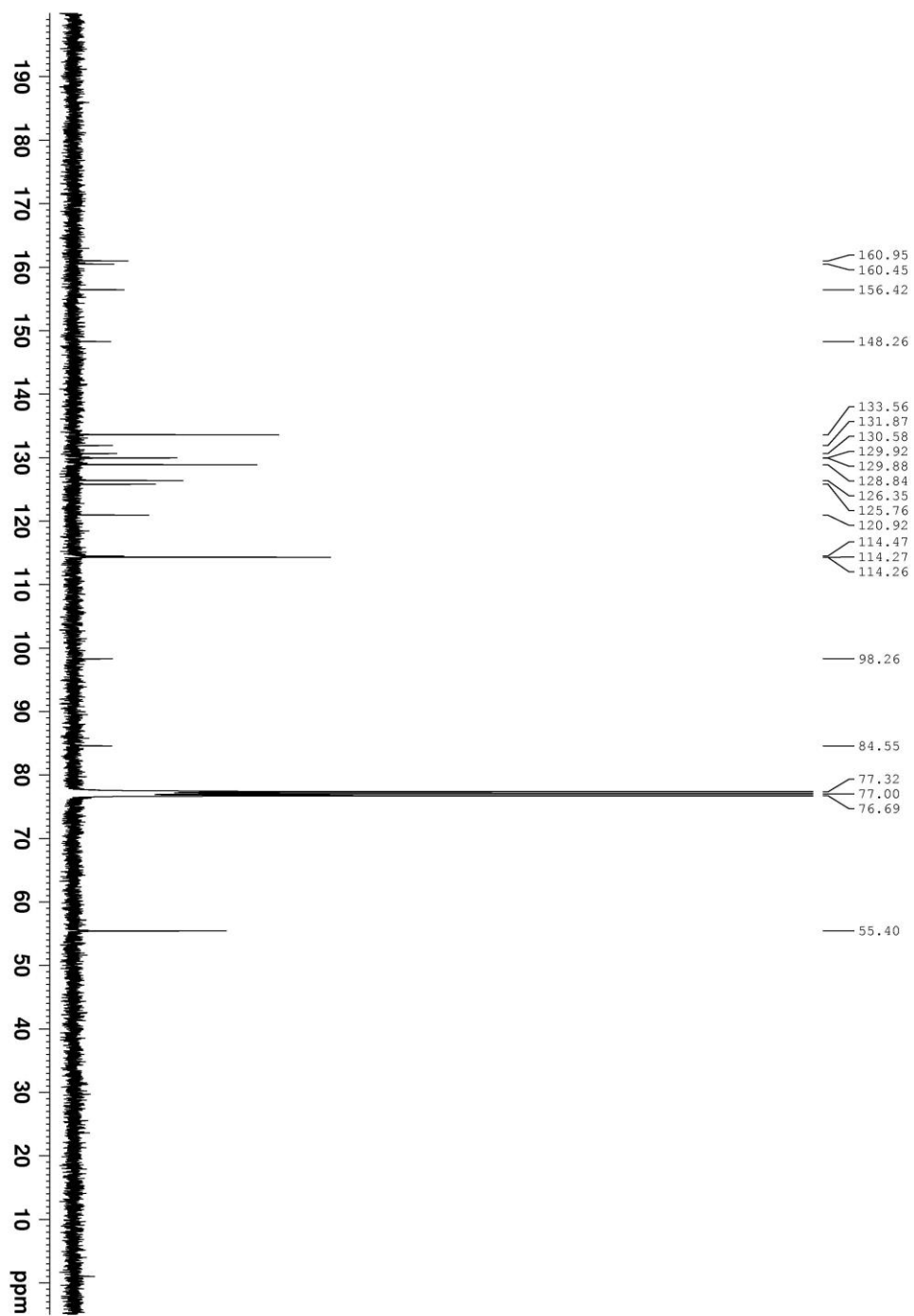

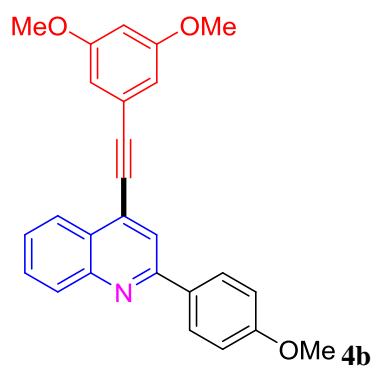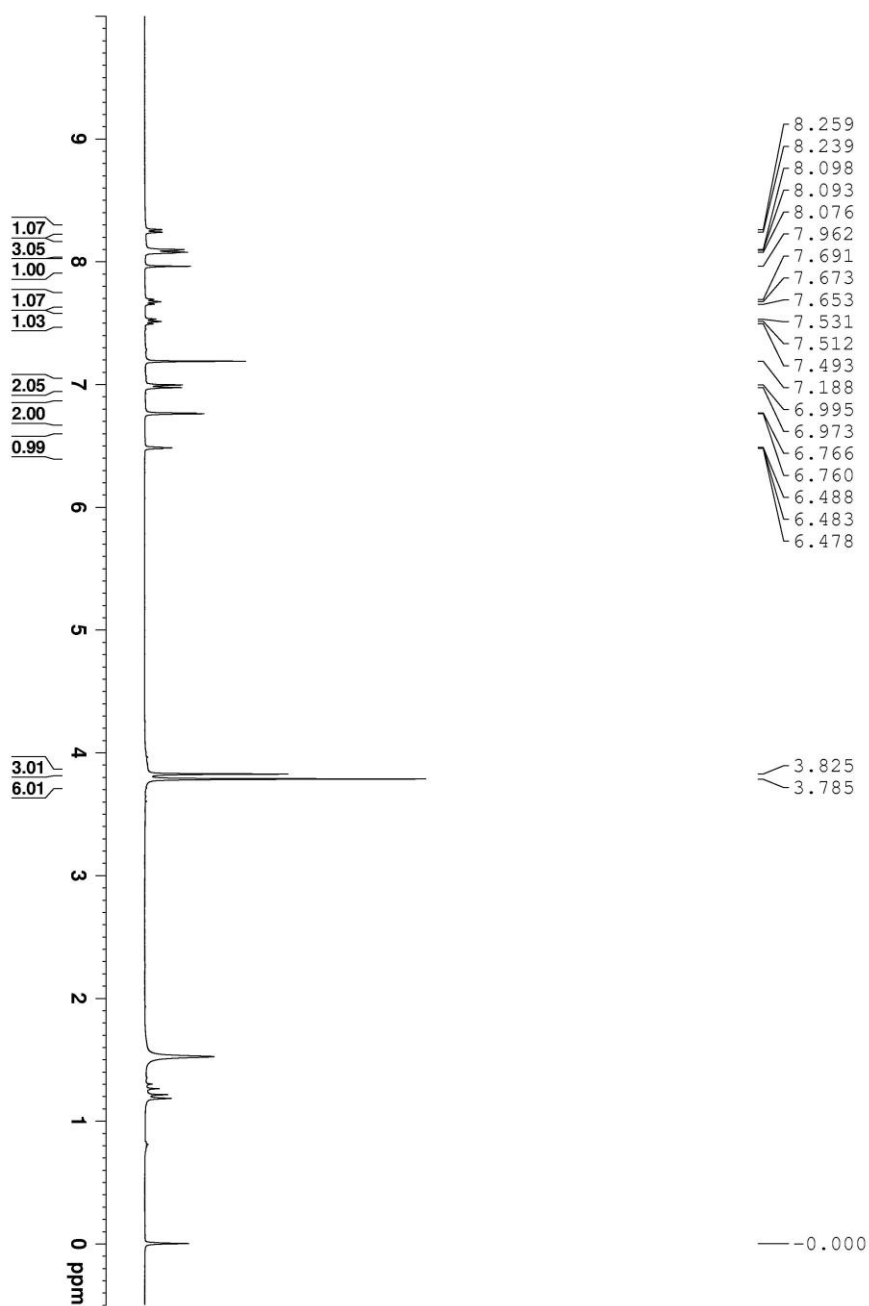

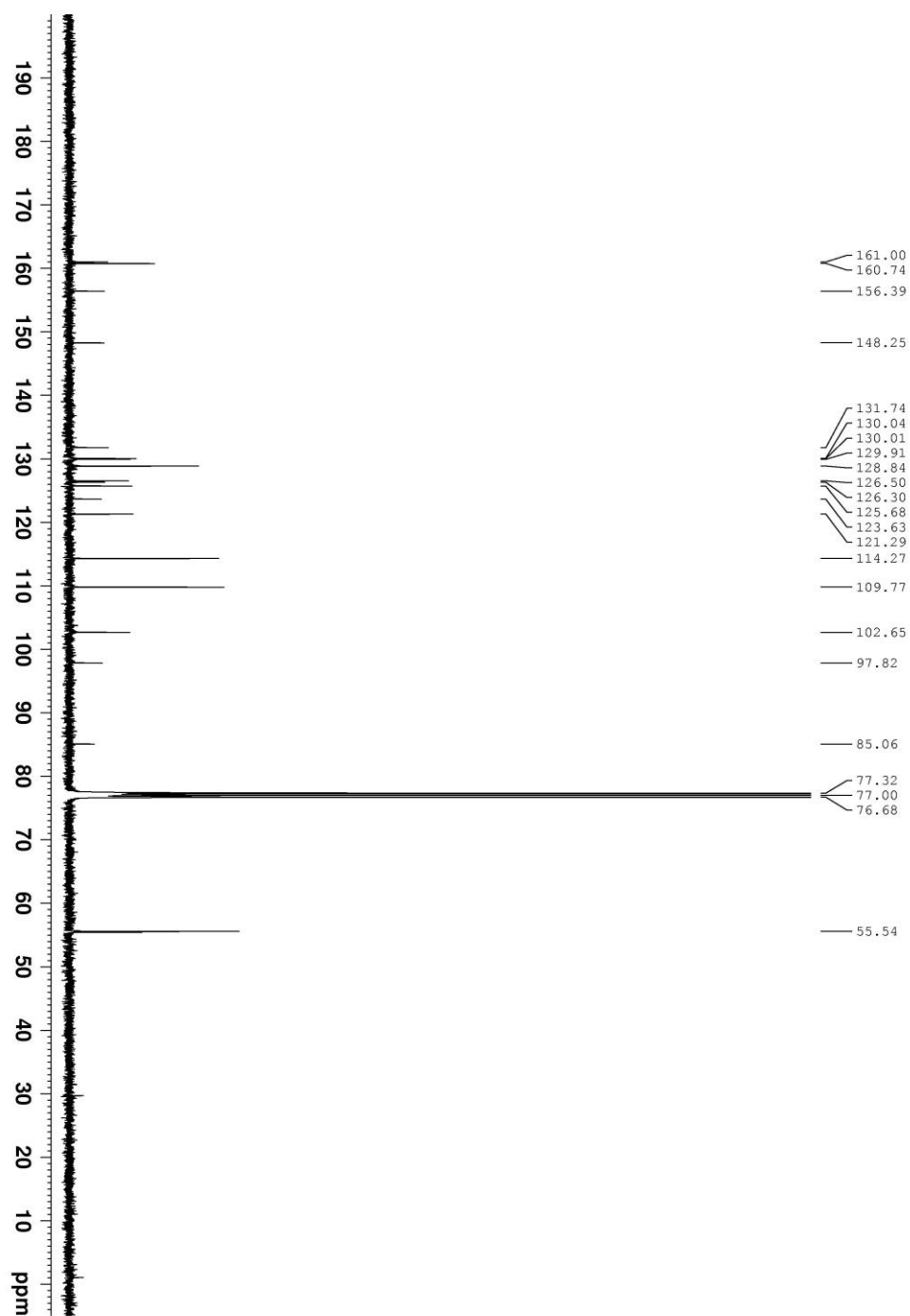

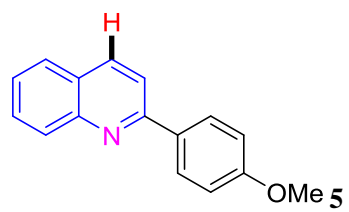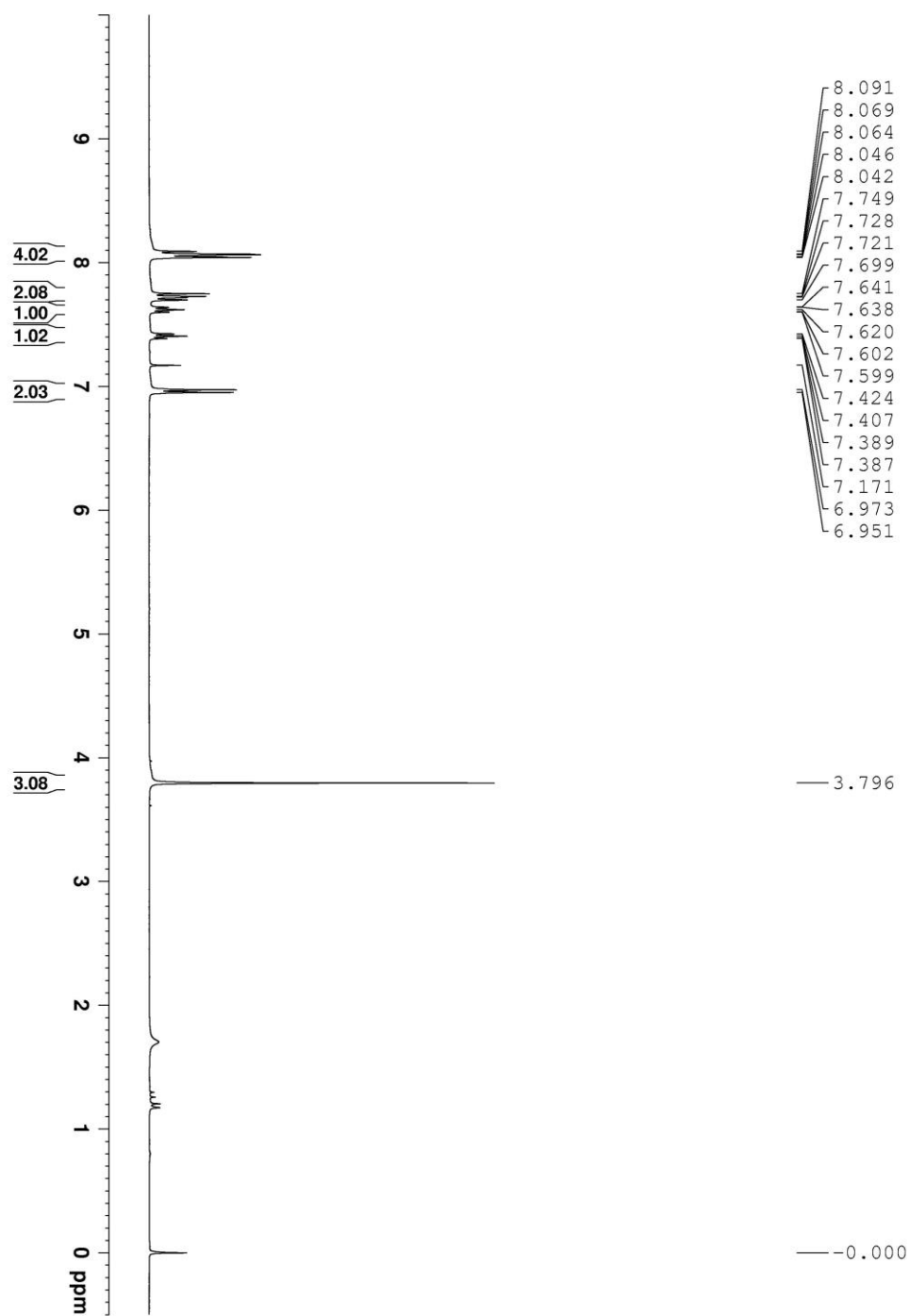

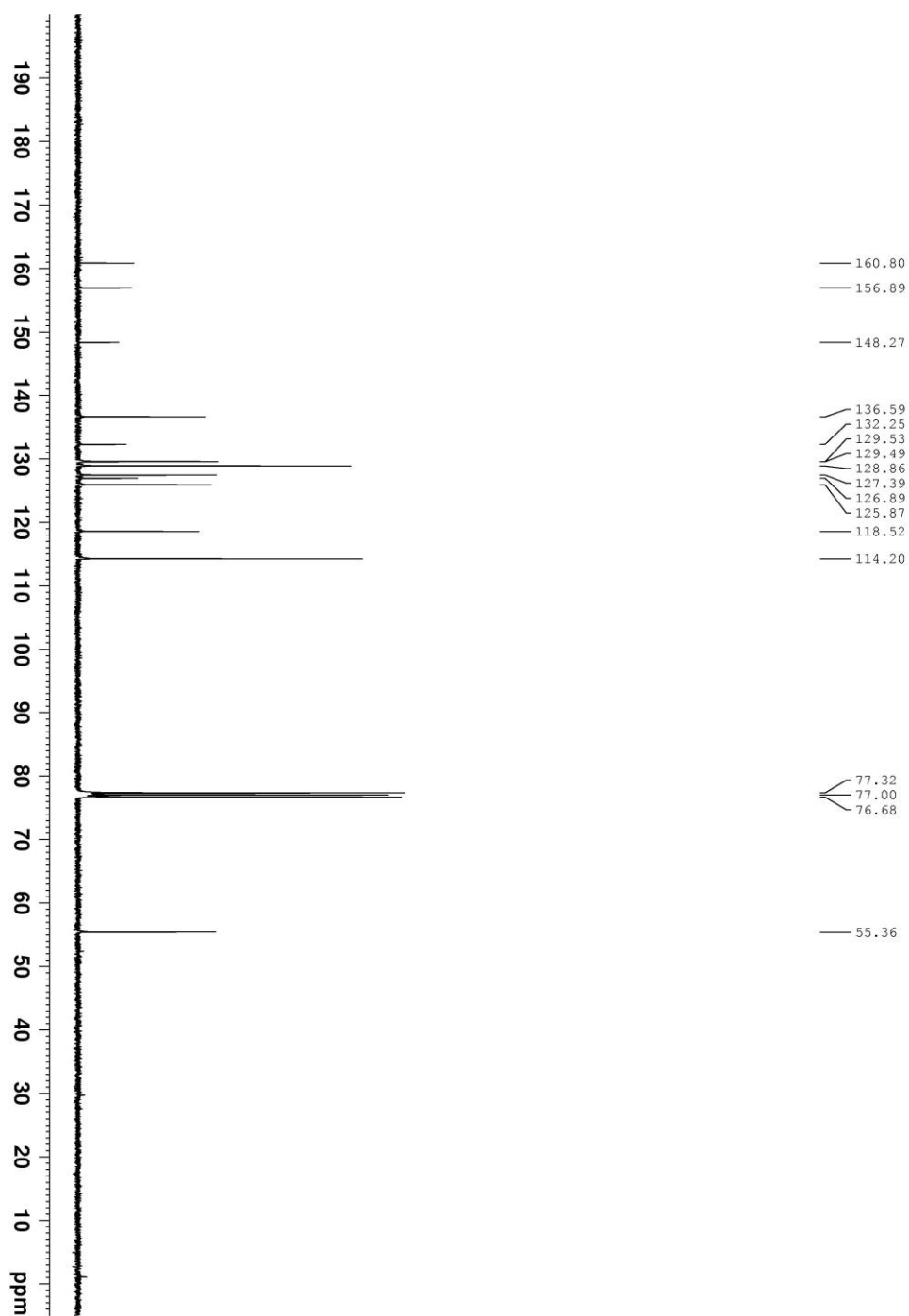

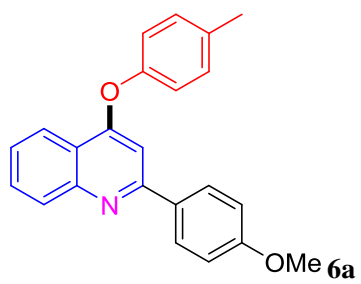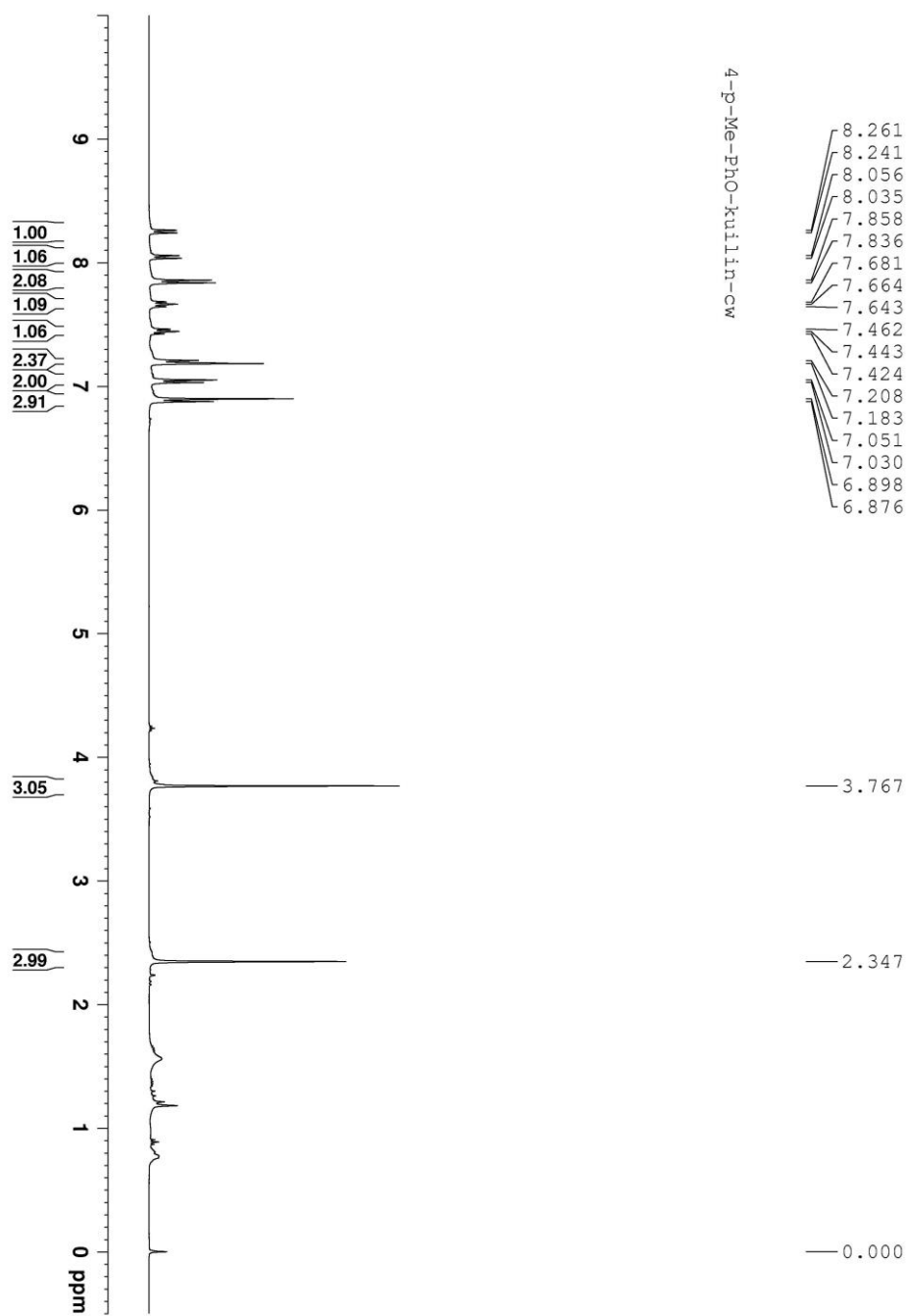

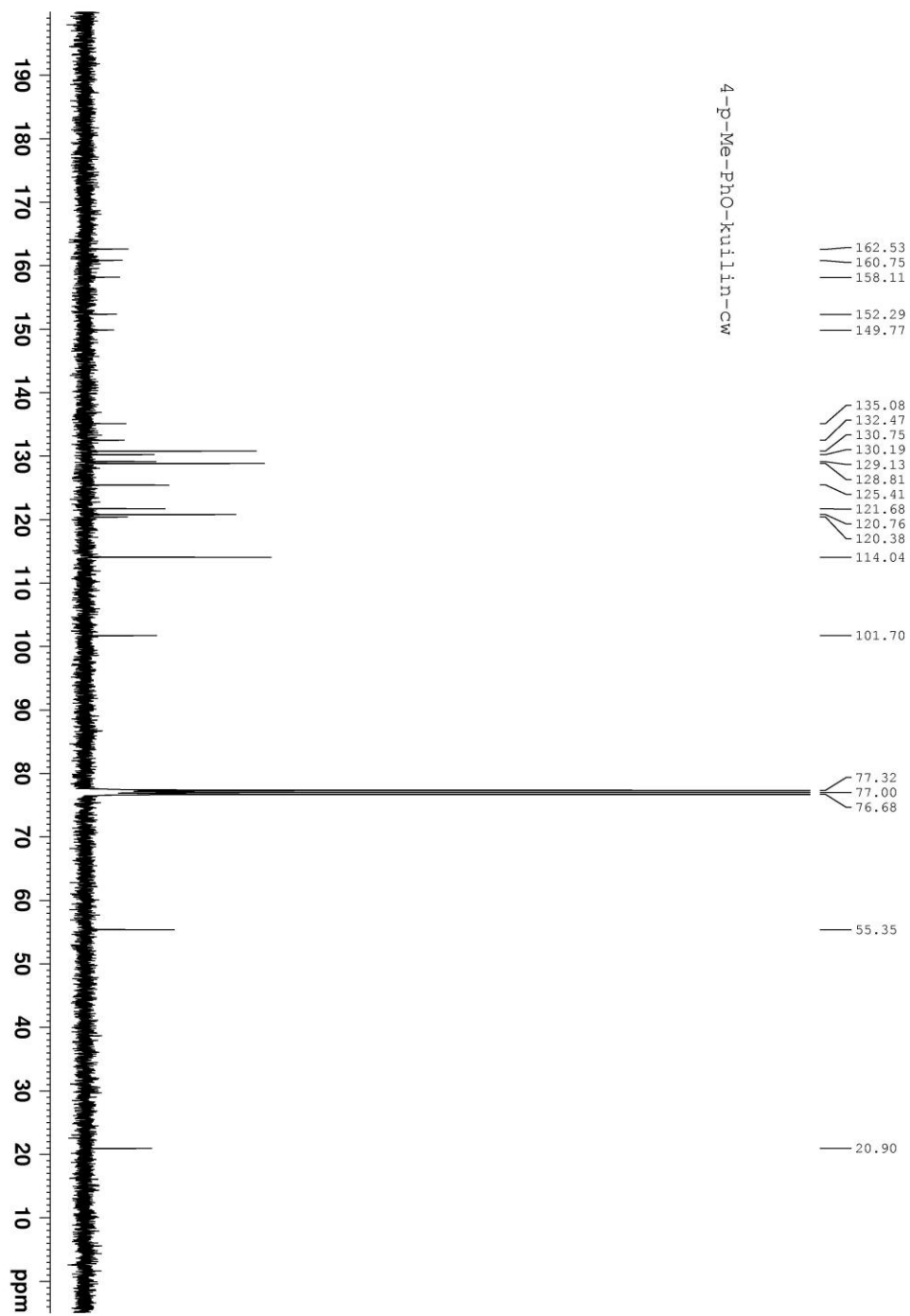

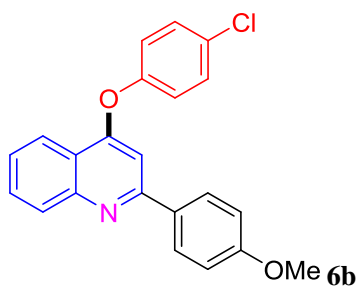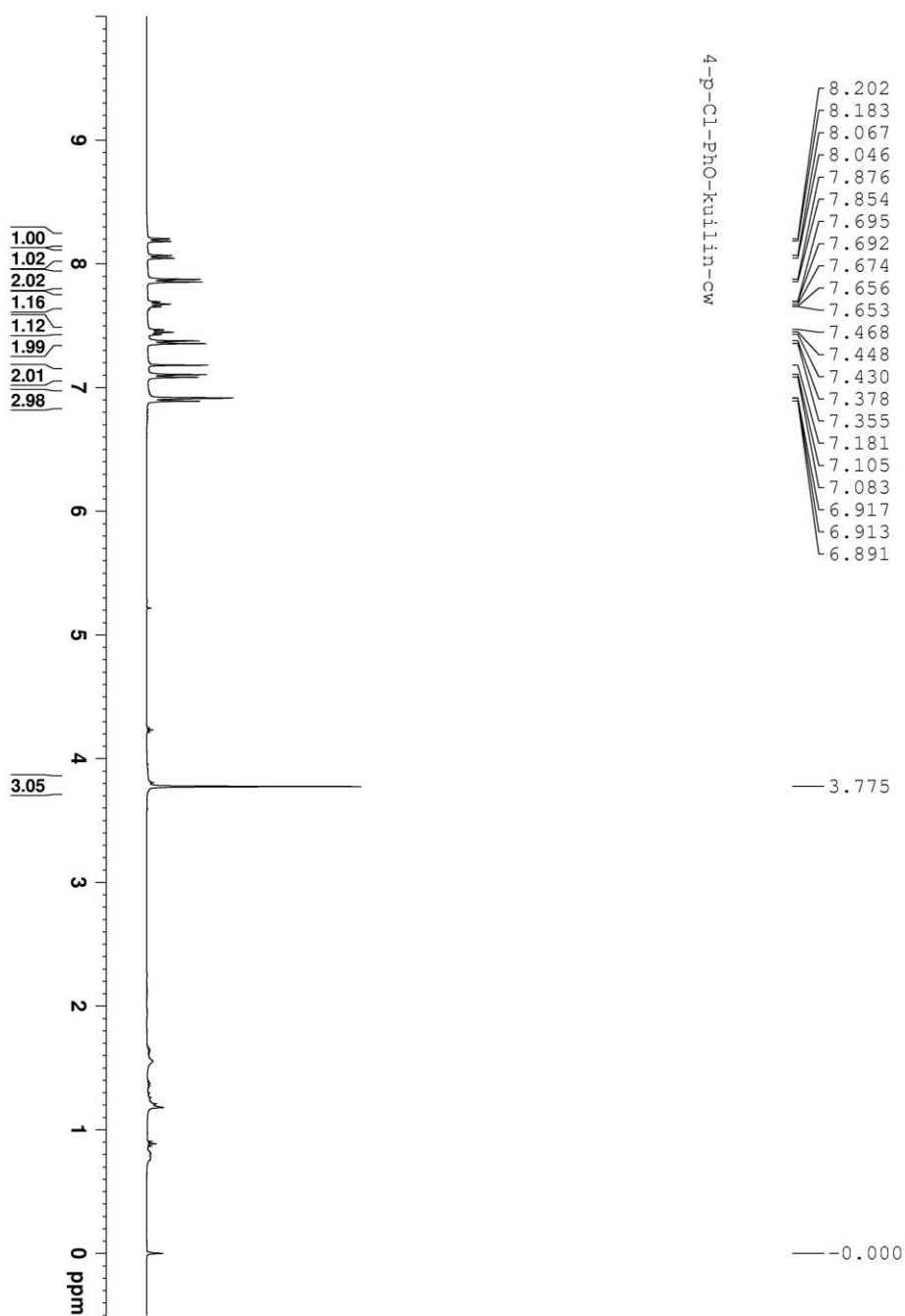

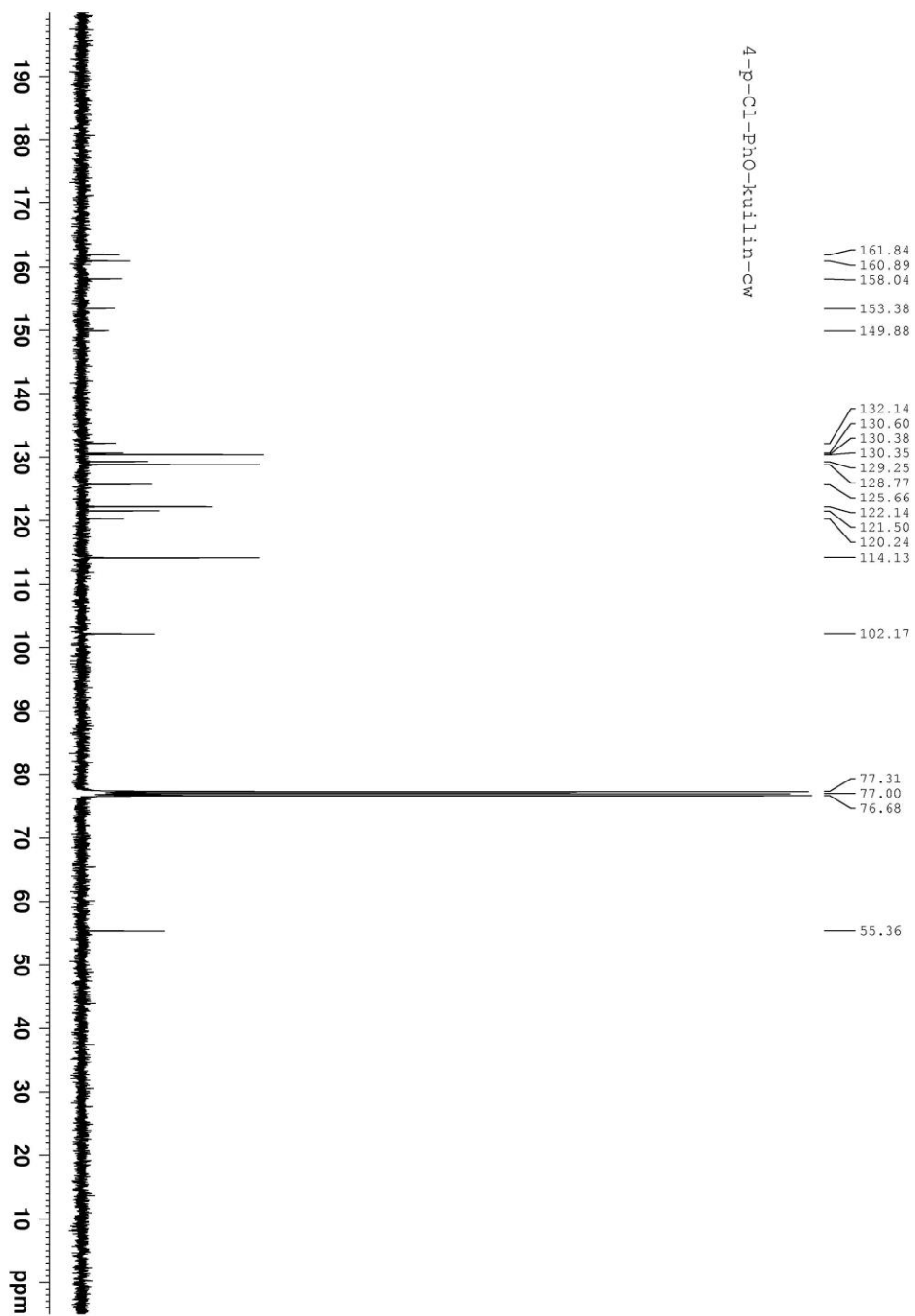

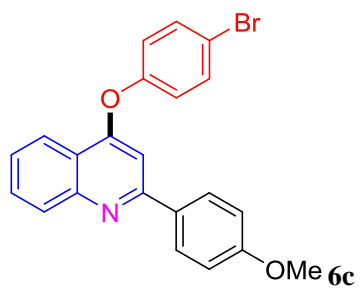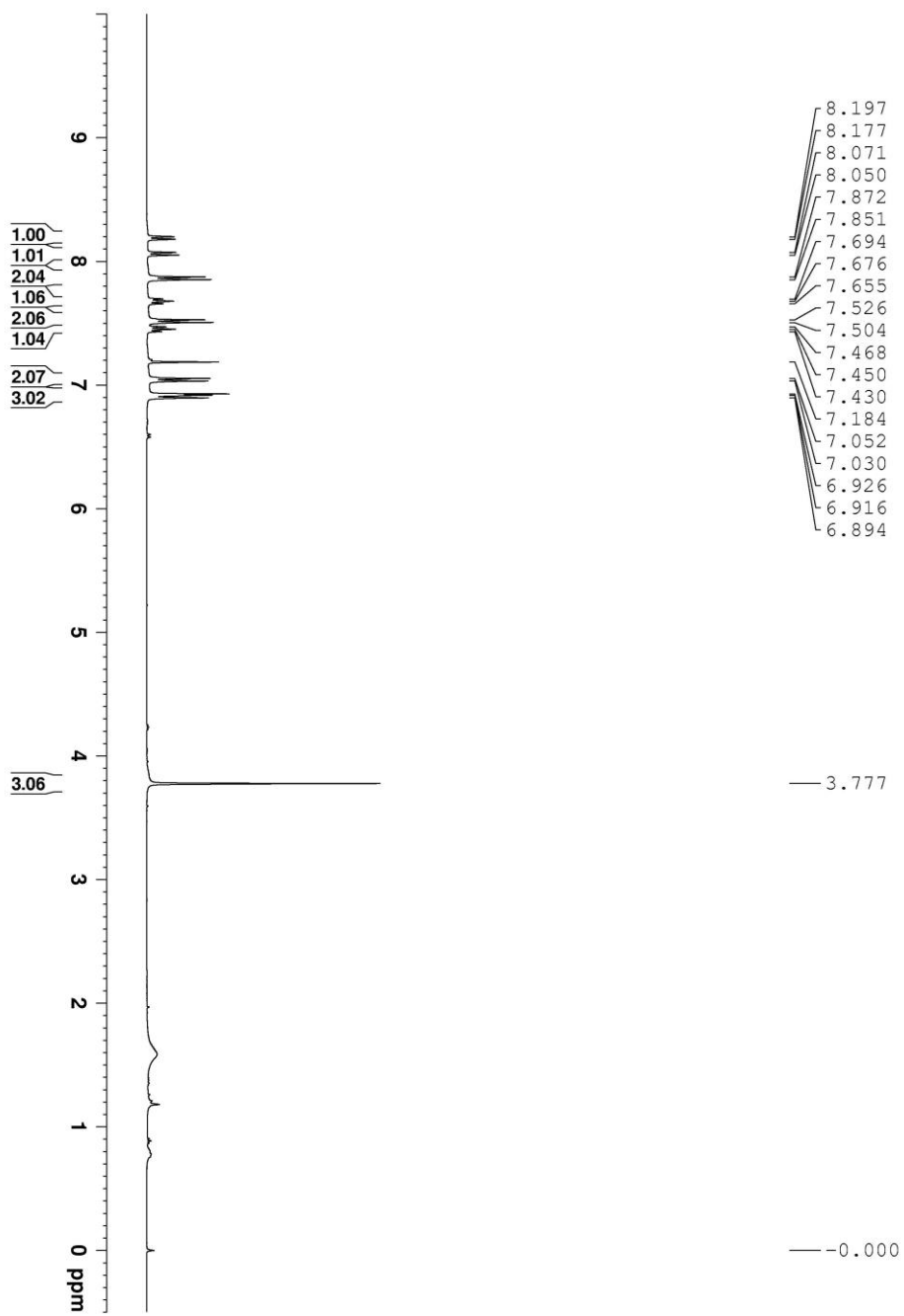

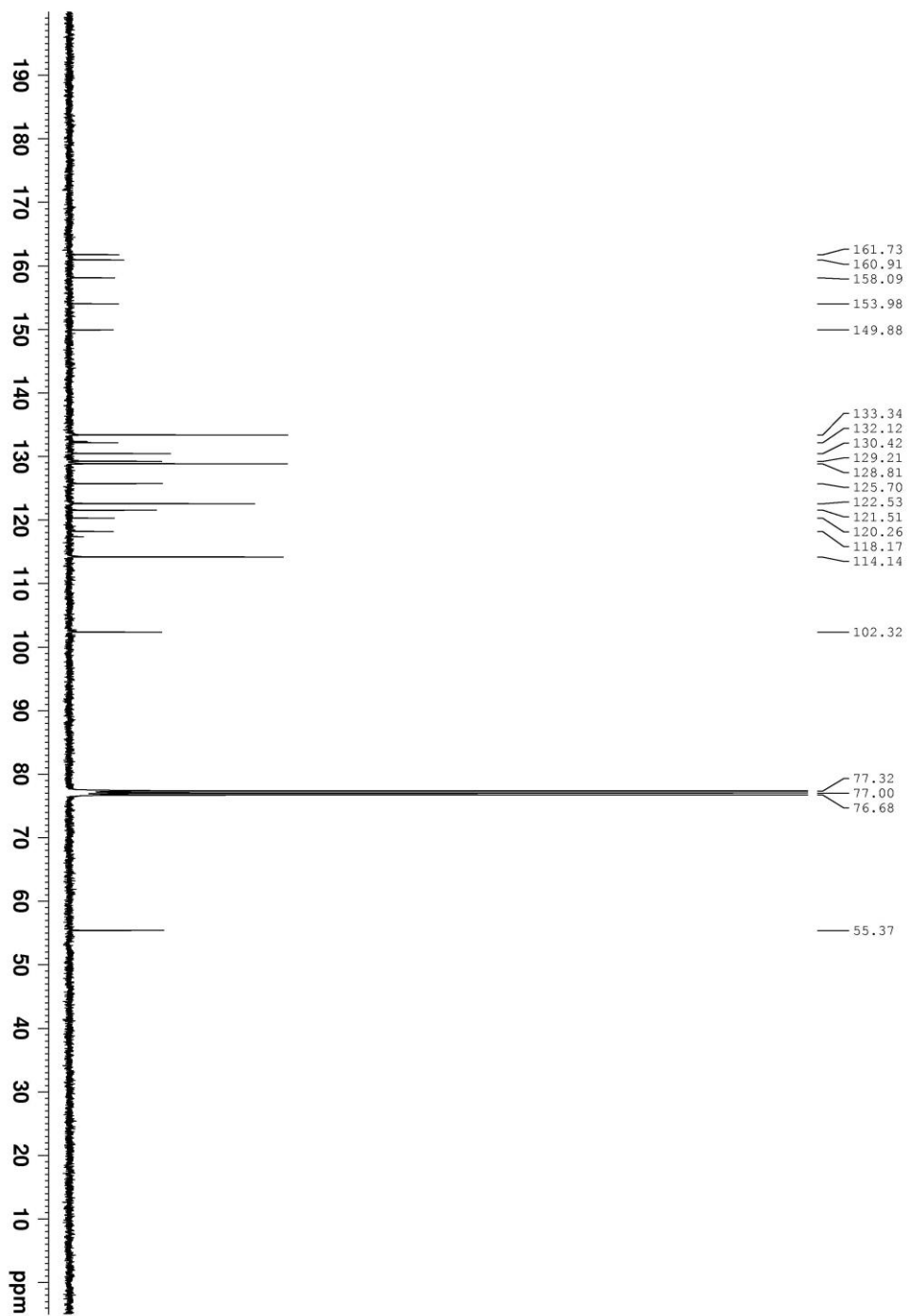

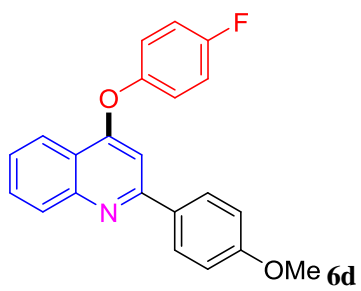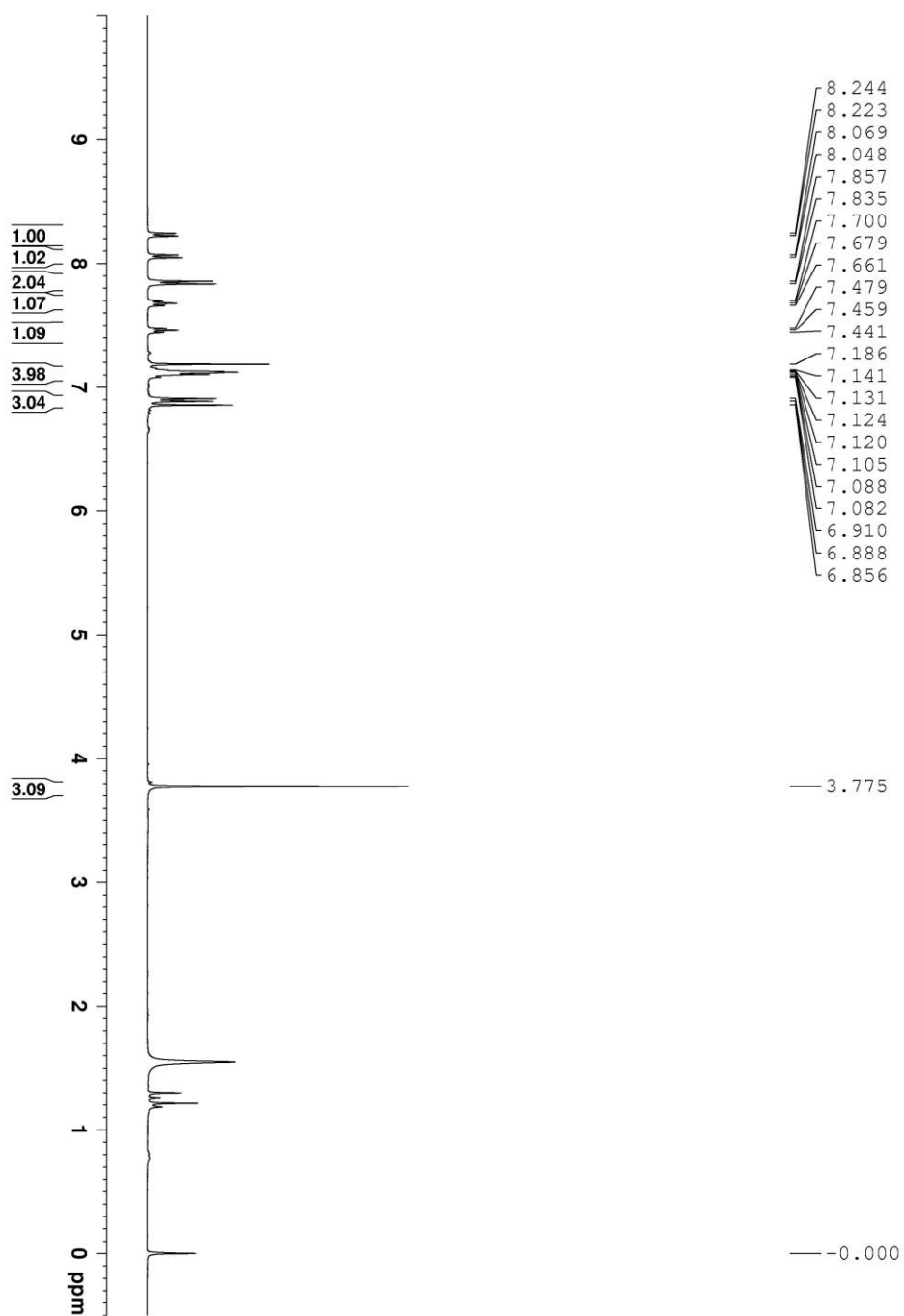

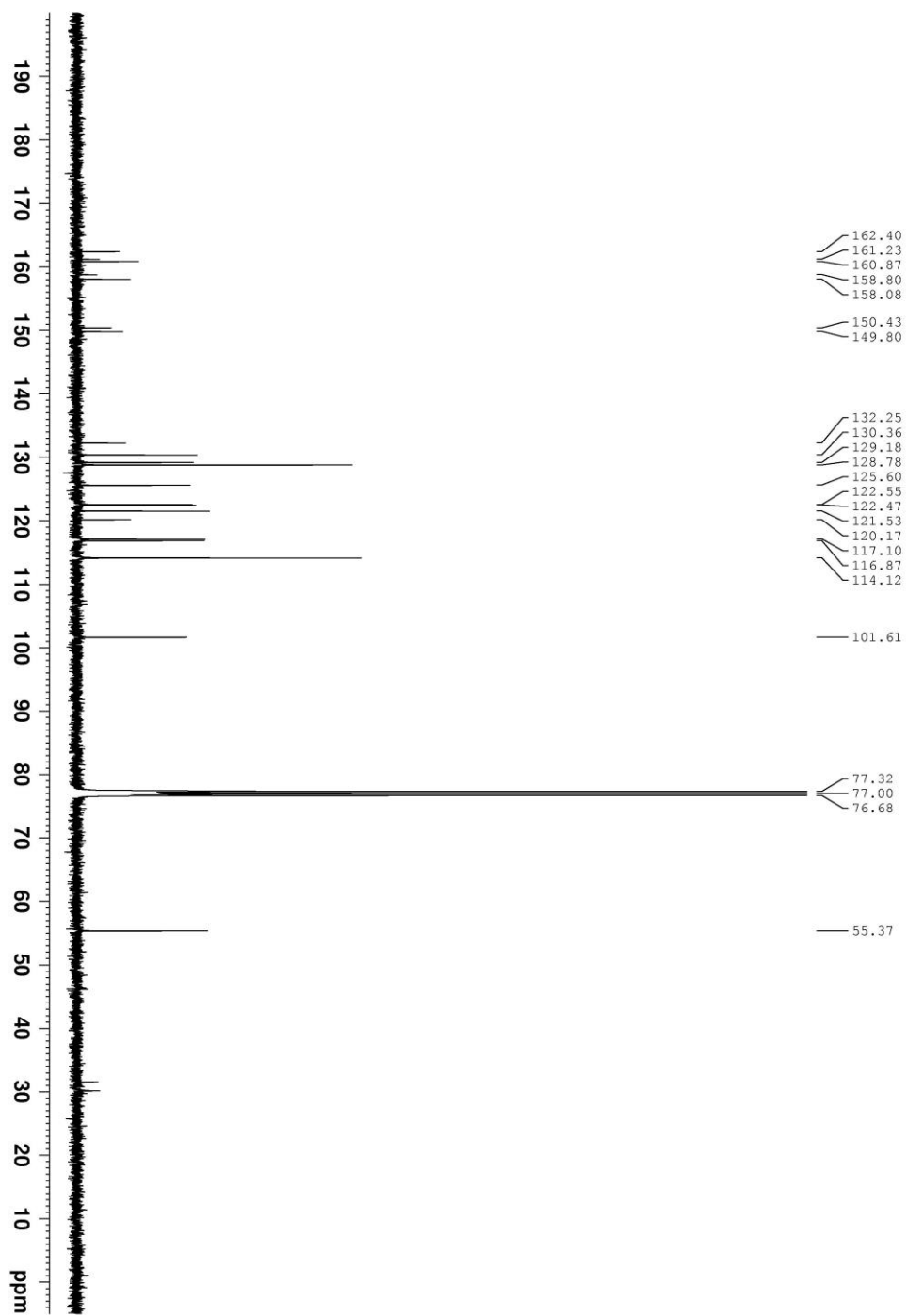

Supplement: Supplementary file 1 [file molecules-24-03999-s001.pdf]
